# Evolution of the avian β-defensin and cathelicidin genes

**DOI:** 10.1186/s12862-015-0465-3

**Published:** 2015-09-15

**Authors:** Yuanyuan Cheng, Michael Dennis Prickett, Weronika Gutowska, Richard Kuo, Katherine Belov, David W. Burt

**Affiliations:** RMC Gunn Building B19, Faculty of Veterinary Science, University of Sydney, Camperdown, 2006 NSW Australia; Dipartimento di Scienze della Vita-Edif. C11, Università di Trieste, Via Licio Giorgieri 1, 34127 Trieste, Italy; The Roslin Institute and Royal (Dick) School of Veterinary Studies, University of Edinburgh, Easter Bush, Midlothian, EH25 9RG UK

## Abstract

**Background:**

β-defensins and cathelicidins are two families of cationic antimicrobial peptides (AMPs) with a broad range of antimicrobial activities that are key components of the innate immune system. Due to their important roles in host defense against rapidly evolving pathogens, the two gene families provide an ideal system for studying adaptive gene evolution. In this study we performed phylogenetic and selection analyses on β-defensins and cathelicidins from 53 avian species representing 32 orders to examine the evolutionary dynamics of these peptides in birds.

**Results and conclusions:**

Avian β-defensins are found in a gene cluster consisting of 13 subfamiles. Nine of these are conserved as one to one orthologs in all birds, while the others (AvBD1, AvBD3, AvBD7 and AvBD14) are more subject to gene duplication or pseudogenisation events in specific avian lineages. Avian cathelicidins are found in a gene cluster consisting of three subfamilies with species-specific duplications and gene loss. Evidence suggested that the propiece and mature peptide domains of avian cathelicidins are possibly co-evolving in such a way that the cationicity of the mature peptide is partially neutralised by the negative charge of the propiece prior to peptide secretion (further evidence obtained by repeating the analyses on primate cathelicidins). Negative selection (overall mean d_N_ < d_S_) was detected in most of the gene domains examined, conserving certain amino acid residues that may be functionally crucial for the avian β-defensins and cathelicidins, while episodic positive selection was also involved in driving the diversification of specific codon sites of certain AMPs in avian evolutionary history. These findings have greatly improved our understanding of the molecular evolution of avian AMPs and will be useful to understand their role in the avian innate immune response. Additionally, the large dataset of β-defensin and cathelicidin peptides may also provide a valuable resource for translational research and development of novel antimicrobial agents in the future.

**Electronic supplementary material:**

The online version of this article (doi:10.1186/s12862-015-0465-3) contains supplementary material, which is available to authorized users.

## Background

Defensins and cathelicidins are two families of cationic small peptides that have broad-spectrum antimicrobial activities against a wide range of bacterial, fungal or viral pathogens. These peptides are produced in a large variety of invertebrate and vertebrate organisms, representing an ancient form of host defense against microbes. In addition to their antimicrobial function, defensins and cathelicidins have also been found to exhibit diverse immunomodulatory activities, rendering them important components of both innate and adaptive immune systems [1–3]. With a key role in host defense against rapidly evolving pathogens, defensin and cathelicidin gene families provide an ideal system for studying adaptive molecular evolution [4]. Previous studies in mammalian lineages have revealed positive selection driving rapid divergence of these host defense peptides [4, 5]. Recent whole-genome sequence analysis of 48 bird species [6] has enabled us to perform a comprehensive comparative analysis on the avian lineages of defensins and cathelicidins, which will not only greatly improve our understanding of the evolutionary diversification of these ancient peptides over the past 100 million years through the avian radiation, but also provide a valuable resource for developing novel antibiotics to treat microbial infections in birds and other vertebrates [7, 8].

Among the three defensin subfamilies (α, β and θ) identified in vertebrates, only β-defensins have been found in birds [9, 10]. Avian β-defensins (AvBDs) have been previously described in the chicken (*Gallus gallus*), zebra finch (*Taeniopygia guttata*), mallard (*Anas platyrhynchos*) and crested ibis (*Nipponia nippon*) with a cluster of 14, 22, 18 and 14 closely linked β-defensin genes found in each genome, respectively [9–13]. AvBD genes usually consist of 3–4 exons [10]. The first exon contains the 5′-untranslated region and the others contain the open reading frame encoding three peptide domains, including a hydrophobic, leucine-rich signal peptide, a short propiece (absent in *AvBD12*) and the mature peptide. The mature β-defensin forms a characteristic β sheet-rich fold, which contains three disulfide bridges (Cys^1^-Cys^5^, Cys^2^-Cys^4^ and Cys^3^-Cys^6^) within a conserved six-cysteine framework. AvBDs have been shown to be expressed in a number of tissues with varying levels of expression [14, 15] and play a role in the innate host defense against viruses, Gram-positive and Gram-negative bacteria, and parasites in the digestive system [16–21] and reproductive system [22–26]. Evidence has also indicated that they may have similar regulatory functions as mammalian β-defensins in various immune processes, such as chemotaxis, wound-healing, and inducing or suppressing inflammatory responses [reviewed in 1]. Within-species genetic diversity and trans-species polymorphisms of AvBDs have been observed in passerine bird species [27, 28].

Cathelicidins are characterised by having a conserved cathelin domain and a highly variable mature peptide domain. Four avian cathelicidin genes have been identified in the chicken, designated *CATHL1*, *CATHL2*, *CATHL3* and *CATHB1* [2, 29–31]. These genes share similar structures with mammalian cathelicidins, each comprising of four exons, encoding a prepropiece consisting of a signal peptide, the cathelin-like domain (propiece) and the mature peptide. One unusual feature found in the propiece of chicken *CATHB1*, is the presence of nine octamer repeats (PGLDGSXS) N-terminal to the cathelin domain [30], which is not seen in other cathelicidin genes. Mature avian cathelicidins are activated before secretion with the propiece cleaved off by serine proteases [32] and form an alpha-helix predominant structure with a kink induced by a glycine or proline close to the centre [33–35]. All four chicken cathelicidins show high levels of expression in immune organs and gastrointestinal, respiratory and urogenital tracts, with *CATHL1*, *2* and *3* most highly expressed in the bone marrow and lung, and *CATHB1* in the bursa of Fabricius [36]. In addition to broad antimicrobial activity against bacteria and fungi, avian cathelicidins also play a range of immunoregulatory roles, such as blocking lipopolysaccharide-induced cytokine expression [37] or inducing specific chemokine production [31].

In this paper, we studied the evolution of β-defensins and cathelicidins in 53 avian species (Table 1) and discussed the following issues: conservation and lineage-specific duplication/deletion of genes, conserved genomic organisation of β-defensin and cathelicidin gene clusters, coevolution between pro- and mature peptides in cathelicidins, and amino acid sites and evolutionary branches under selection.

**Table 1:** List of 53 examined bird species

Scientific name	Common name	Abbreviation	Order	Family	Genus	GenBank Assembly ID	AvBD1 paralogues	AvBD3 paralogues	Pseudogenes	Genes not found	Cluster status
*Acanthisitta chloris*	Rifleman	ACACH	Passeriformes	Acanthisittidae	Acanthisitta	GCA_000695815	2	5	AvBD11, AvBD14		fragmented
*Amazona vittata*	Puerto Rican parrot	AMAVI	Psittaciformes	Psittacidae	Amazona	GCA_000332375	2	1	AvBD7, AvBD14		fragmented
*Anas platyrhynchos*	American Pekin duck	ANAPL	Anseriformes	Anatidae	Anas	GCA_000355885	1	6			fragmented
*Apaloderma vittatum*	Bar-tailed trogon	APAVI	Trogoniformes	Trogonidae	Apaloderma	GCA_000703405	1	2		AvBD14	fragmented
*Aptenodytes forsteri*	Emperor penguin	APTFO	Sphenisciformes	Spheniscidae	Aptenodytes	GCA_000699145	1	1			intact
*Balearica regulorum gibbericeps*	Grey crowned crane	BALRE	Gruiformes	Gruidae	Balearica	GCA_000709895	1	2			fragmented
*Buceros rhinoceros silvestris*	Rhinoceros hornbill	BUCRH	Bucerotiformes	Bucerotidae	Buceros	GCA_000710305	1	0		AvBD3, AvBD12	fragmented
*Calypte anna*	Anna’s hummingbird	CALAN	Trochiliformes	Trochilidae	Calypte	GCA_000699085	1	1	AvBD7, AvBD14	AvBD11	fragmented
*Caprimulgus carolinensis*	Chuck-will’s-widow	CAPCA	Caprimulgiformes	Caprimulgidae	Antrostomus	GCA_000700745	2	1			fragmented
*Cariama cristata*	Red-legged seriema	CARCR	Gruiformes	Cariamidae	Cariama	GCA_000690535	2	1			fragmented
*Cathartes aura*	Turkey vulture	CATAU	Cathartiformes	Cathartidae	Cathartes	GCA_000699945	1	0		AvBD7	fragmented
*Chaetura pelagica*	Chimney swift	CHAPE	Apodiformes	Apodidae	Chaetura	GCA_000747805	1	3	AvBD7, AvBD11		fragmented
*Charadrius vociferus*	Killdeer	CHAVO	Charadriiformes	Charadriidae	Charadrius	GCA_000708025	3	2			fragmented
*Chlamydotis undulata*	Houbara bustard	CHLUN	Gruiformes	Otididae	Chlamydotis	GCA_000695195	2	1		AvBD14	fragmented
*Colinus virginianus*	Northern bobwhite	COLVI	Galliformes	Odontophoridae	Colinus	GCA_000599465	1	1			fragmented^a^
*Colius striatus*	Speckled mousebird	COLST	Coliiformes	Coliidae	Colius	GCA_000690715	1	1		AvBD13	fragmented^a^
*Columba livia*	Rock dove	COLLI	Columbiformes	Columbidae	Columba	GCA_000337935	1	2	AvBD14		fragmented^a^
*Corvus brachyrhynchos*	American crow	CORBR	Passeriformes	Corvidae	Corvus	GCA_000691975	2	6	AvBD14		fragmented
*Cuculus canorus*	Common cuckoo	CUCCA	Cuculiformes	Cuculidae	Cuculus	GCA_000709325	2	2			fragmented^a^
*Egretta garzetta*	Little egret	EGRGA	Ciconiiformes	Ardeidae	Egretta	GCA_000687185	2	1		AvBD14	fragmented
*Eurypyga helias*	Sunbittern	EURHE	Gruiformes	Eurypygidae	Eurypyga	GCA_000690775	1	1	AvBD7	AvBD14	fragmented
*Falco cherrug*	Saker falcon	FALCH	Falconiformes	Falconidae	Falco	GCA_000337975	3	1	AvBD14		fragmented^a^
*Falco peregrinus*	Peregrine falcon	FALPE	Falconiformes	Falconidae	Falco	GCA_000337955	2	1	AvBD14	AvBD3.3	fragmented
*Ficedula albicollis*	Collared flycatcher	FICAL	Passeriformes	Muscicapidae	Ficedula	GCA_000247815	2	6	AvBD14		fragmented
*Fulmarus glacialis*	Northern fulmar	FULGL	Procellariiformes	Procellariidae	Fulmarus	GCA_000690835	2	1	AvBD1.1	Exon2 AvBD13	fragmented
*Gallus gallus*	Chicken	GALGA	Galliformes	Phasianidae	Gallus	GCA_000002315	1	1			intact
*Gavia stellata*	Red-throated loon	GAVST	Gaviiformes	Gaviidae	Gavia	GCA_000690875	1	1	AvBD14		fragmented
*Geospiza fortis*	Medium ground finch	GEOFO	Passeriformes	Fringillidae	Geospiza	GCA_000277835	3	9	AvBD14		intact
*Haliaeetus albicilla*	White-tailed eagle	HALAL	Accipitriformes	Accipitridae	Haliaeetus	GCA_000691405	1	1		AvBD13	fragmented
*Leptosomus discolor*	Cuckoo roller	LEPDI	Coraciiformes	Leptosomidae	Leptosomus	GCA_000691785	1	1		AvBD2	fragmented
*Manacus vitellinus*	Golden-collared manakin	MANVI	Passeriformes	Pipridae	Manacus	GCA_000692015	1	7	AvBD14		fragmented
*Meleagris gallopavo*	Turkey	MELGA	Galliformes	Phasianidae	Meleagris	GCA_000146605	1	1			intact
*Melopsittacus undulatus*	Budgerigar	MELUN	Psittaciformes	Psittacidae	Melopsittacus	GCA_000238935	2	1	AvBD7, AvBD14		intact
*Merops nubicus*	Carmine bee-eater	MERNU	Coraciiformes	Meropidae	Merops	GCA_000691845	1	1			fragmented
*Mesitornis unicolor*	Brown roatelo	MESUN	Gruiformes	Mesitornithidae	Mesitornis	GCA_000695765	2	3		AvBD14	fragmented
*Nestor notabilis*	Kea	NESNO	Psittaciformes	Psittacidae	Nestor	GCA_000696875	1	1	AvBD7	AvBD14	fragmented
*Nipponia nippon*	Crested ibis	NIPNI	Ciconiiformes	Threskiornithidae	Nipponia	GCA_000708225	2	1			intact
*Opisthocomus hoazin*	Hoatzin	OPIHO	Opisthocomiformes	Opisthocomidae	Opisthocomus	GCA_000692075	1	1	AvBD9		fragmented^a^
*Pelecanus crispus*	Dalmatian pelican	PELCR	Pelecaniformes	Pelecanidae	Pelecanus	GCA_000687375	1	1		Exon 2 AvBD13	fragmented^a^
*Phaethon lepturus*	White-tailed tropicbird	PHALE	Phaethontiformes	Phaethontidae	Phaethon	GCA_000687285	2	2			fragmented
*Phalacrocorax carbo*	Great cormorant	PHACA	Pelecaniformes	Phalacrocoracidae	Phalacrocorax	GCA_000708925	1	1		AvBD4, AvBD11, AvBD14	fragmented
*Phoenicopterus ruber*	American flamingo	PHORU	Phoenicopteriformes	Phoenicopteridae	Phoenicopterus	GCA_000687265	2	2		AvBD14	fragmented
*Picoides pubescens*	Downy woodpecker	PICPU	Piciformes	Picidae	Picoides	GCA_000699005	1	1	AvBD7		fragmented
*Podiceps cristatus*	Great crested grebe	PODCR	Podicipediformes	Podicipedidae	Podiceps	GCA_000699545	2	1		AvBD14	fragmented
*Pseudopodoces humilis*	Tibetan ground-tit	PSEHU	Passeriformes	Paridae	Pseudopodoces	GCA_000331425	2	7	AvBD14		intact
*Pterocles gutturalis*	Yellow-throated sandgrouse	PTEGU	Ciconiiformes	Pteroclidae	Pterocles	GCA_000699245	2	1			fragmented
*Pygoscelis adeliae*	Adelie penguin	PYGAD	Sphenisciformes	Spheniscidae	Pygoscelis	GCA_000699105	1	1			intact
*Struthio camelus*	African ostrich	STRCA	Struthioniformes	Struthionidae	Struthio	GCA_000698965	2	2			fragmented
*Taeniopygia guttata*	Zebra finch	TAEGU	Passeriformes	Estrildidae	Taeniopygia	GCA_000151805	3	9	AvBD14		intact
*Tauraco erythrolophus*	Red-crested turaco	TAUER	Musophagiformes	Musophagidae	Tauraco	GCA_000709365	2	2	AvBD7	AvBD14	fragmented
*Tinamus major*	Great tinamou	TINMA	Tinamiformes	Tinamidae	Tinamus	GCA_000705375	1	1			fragmented
*Tyto alba*	Barn owl	TYTAL	Strigiformes	Tytonidae	Tyto	GCA_000687205	1	1		AvBD5, AvBD13, AvBD14	fragmented
*Zonotrichia albicollis*	White-throated sparrow	ZONAL	Passeriformes	Fringillidae	Zonotrichia	GCA_000385455	2	14	AvBD14		fragmented

^a^ indicates an intact region between AvBD2 and AvBD5 in the genomic assembly, thus the actual number and position of AvBD1(s) and AvBD3(s) can be determined

## Results and discussion

### Avian β-defensins and cathelicidins

**Fig. 1:**
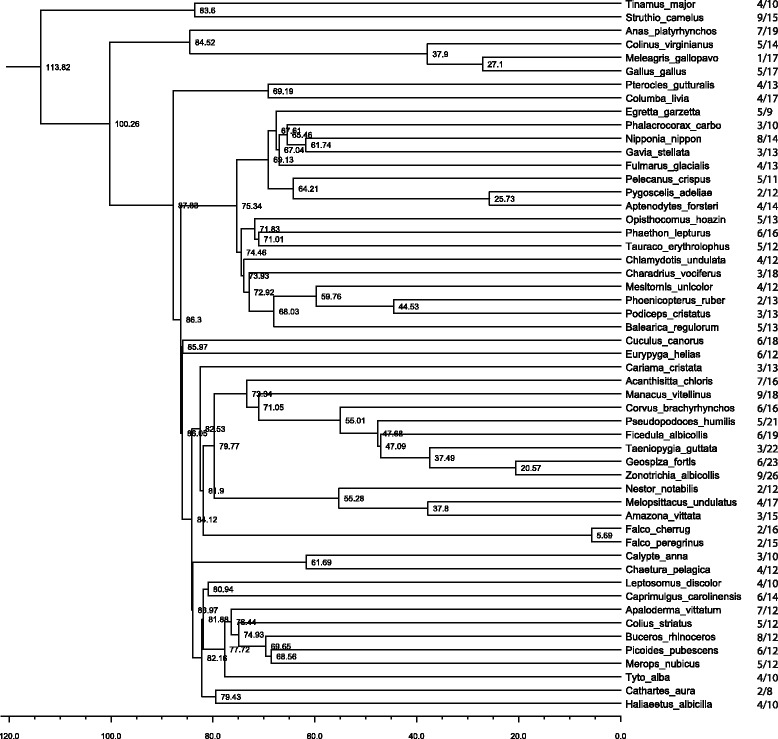
Phylogenetic tree showing evolutionary relationships between 53 examined bird species. The tree was generated via webserver http://birdtree.org/ [38]. Branch length indicates evolutionary time; numbers besides the nodes are estimated divergence time (million years before present). The number of genes detected to have undergone episodic diversifying selection over total number of annotated genes in each species is shown to the right of the species name

A total of 758 genes, including 714 β-defensins and 44 cathelicidins, were annotated in 53 avian genomes (scaffold number and gene coordinates provided in Additional file 1; nucleotide sequences in Additional file 2). The selected species represent 32 different avian orders spanning 52 genera (Table 1), last sharing a common ancestor around 114 million years ago (Fig. 1) [38].

**Fig. 2:**
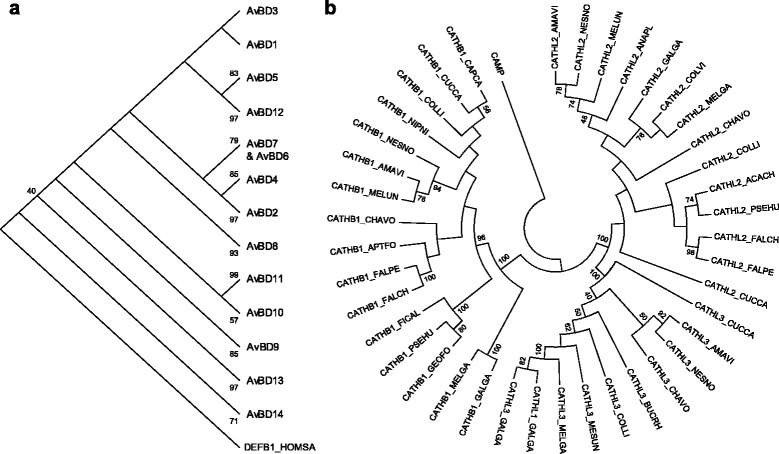
Phylogenetic relationships of avian (**a**) β-defensin and (**b**) cathelicidin subfamilies. Bootstrap values higher than 40 % are shown next to the branches

Annotated genes were designated based on their orthologous relationships with chicken β-defensins (*AvBD1-AvBD14*) and cathelicidins (*CATHL1*, *CATHL2*, *CATHL3* and *CATHB1*). Phylogenetic analysis revealed 13 distinct AvBD groups, namely AvBD1, AvBD2, AvBD3, AvBD4, AvBD5, AvBD6 and AvBD7, AvBD8, AvBD9, AvBD10, AvBD11, AvBD12, AvBD13, and AvBD14 (Fig. 2a), while cathelicidin genes cluster into three clades, CATHL1 and CATHL3, CATHL2, and CATHB1 (Fig. 2b).

**Fig. 3:**
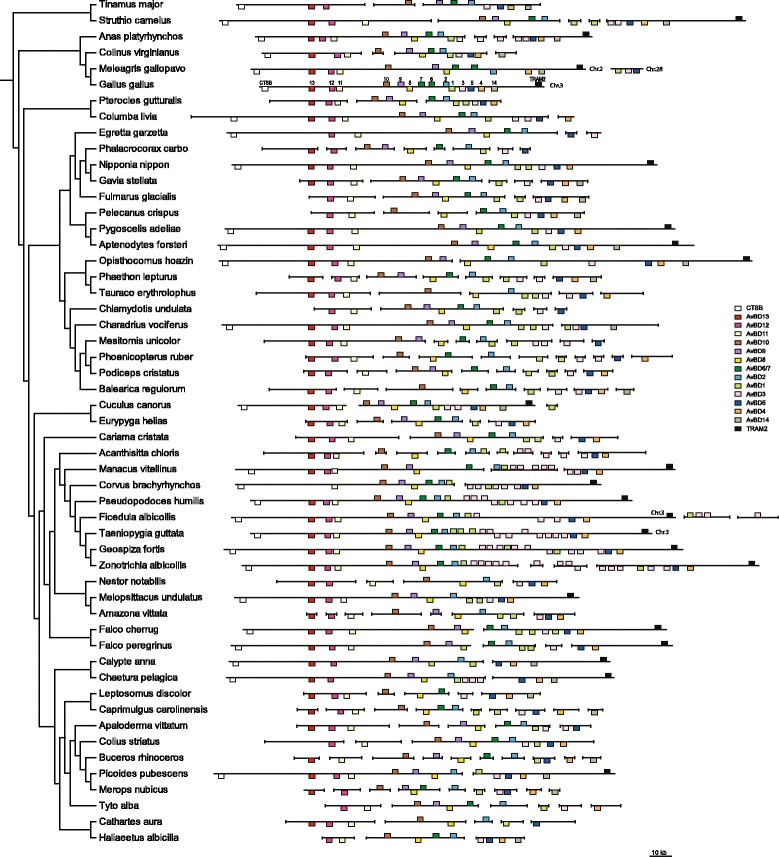
Schematic diagram of avian β-defensin gene clusters

Characteristics of AvBD gene subfamilies are summarised in Table 2 (alignments shown in Additional file 3). Ten genes (AvBD2, 4, 5, 7, 8, 9, 10, 11, 12 and 13) are conserved as one-to-one orthologues in most surveyed species, suggesting a high level of conservation of these genes for over 100 million years (Fig. 3). *AvBD1* and *AvBD3* are subject to lineage-specific expansion, with up to three *AvBD1* paralogues found in the killdeer (*Charadrius vociferus*), saker falcon (*Falco cherrug*), medium ground finch (*Geospiza fortis*) and zebra finch; extensive *AvBD3* duplications occurred in Passeriformes with up to 14 paralogues found in the white-throated sparrow (*Zonotrichia albicollis*). *AvBD6* is a duplication of *AvBD7*, which has arisen within Galliformes after the divergence of family Odontophoridae and before Phasianidae, as it is present in chicken and turkey but absent in the northern bobwhite (*Colinus virginianus*). *AvBD7* has degenerated into a pseudogene in the sunbittern (*Eurpyga helias*) and is missing in all three psittacines. *AvBD14* was found in 23 species and has been degraded into a pseudogene in the orders Falconiformes, Passeriformes and Psittaciformes, and species *Columbia livia*, *Gavia stellata*, and *Calypte anna*.

**Table 2:** Characteristics of avian β-defensin subfamilies

Gene^a^	Type	Defensin motif^b^	Coding exons^c^
AvBD13	one to one ortholog	(9) C (6) C (3) C (9) C (6) CC	2 (3 in Galliformes)
AvBD12	one to one ortholog	(4–6) C (6) C (4) C (9) C (6) CC (10–11)	2
AvBD11	one to one ortholog	(9) C (6) C (5) C (9) C (6) CC (9) C (6) C (6) C (7) C (6) CC	3 (defensin motif duplicated)
AvBD10	one to one ortholog	(11) C (6–7) C (4) C (9) C (6) CC	3
AvBD9	one to one ortholog	(10) C (6) C (4) C (9) C (6) CC	3
AvBD8	one to one ortholog	(11) C (6) C (4) C (10) C (5) CC	3
AvBD7	Duplicated as AvBD6 in Galliformes	(11) C (6) C (4) C (9) C (5) CC	3
AvBD2	one to one ortholog	(11) C (4) C (4) C (9) C (5) CC	3 (3rd exon: 14 residues in Passeriformes)
AvBD1	Up to 3 paralogs in a single species	(11) C (6) C (4) C (9) C (5) CC	3
AvBD3	Up to 14 paralogs in a single species	(5–7) C (6) C (4) C (9) C (5) CC	3 (3rd exon: 12–14 residues in Galliformes)
AvBD5	one to one ortholog	(11) C (6) C (4) C (9) C (5) CC	3
AvBD4	one to one ortholog	(9–14) C (6) C (4) C (9) C (5) CC	2–3
AvBD14	One to one ortholog	(6) C (6) C (4) C (9) C (6) CC	2

^a^ Genes are listed in the order from the flanking gene cathepsin B to the flanking gene TRAM2

^b^ The number of residues between cysteines are noted in parenthesis

^c^ Third exons only code for 2–5 residues unless otherwise noted

The spacing between cysteines within the defensin motif is conserved within subfamilies with the overall consensus being C (4-7) C (3-6) C (7-10) C (5-6) CC, numbers representing the number of residues between cysteines (Table 2). The majority of subfamilies have 9–11 residues preceding the first cysteine of the defensin motif. Exceptions include the group of passeriforme AvBD3s immediately preceding AvBD5 which have 2 residues prior to the first cysteine in addition to having only 2 exons. AvBD1 and AVBD3 have the same spacing of cysteines but differ in the number of residues prior to the first cysteine.

**Fig. 4:**
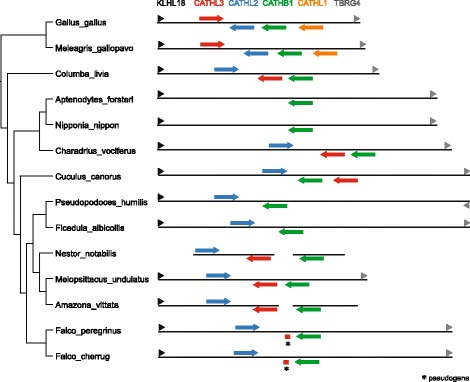
Schematic diagram of avian cathelicidin gene clusters

Cathelicidins were only found in 21 surveyed species due to low assembly quality of the genomic regions. Similar to the 10 relatively conserved AvBDs, *CATHL2*, *CATHL3* and *CATHB1* have been conserved across a variety of avian orders (Fig. 4). Evidence suggested that *CATHL3* has been reduced to a pseudogene in Falconiformes and lost in Passeriformes, yet duplicated to give rise to *CATHL1* in Galliformes. Annotation results also suggested that *CATHL2* and *CATHL3* may have been lost in Sphenisciformes and Ciconiiformes, represented by the emperor penguin (*Aptenodytes forsteri*) and crested ibis (*Nipponia nippon*), respectively (Fig. 4), though this could be an artefact caused by high degree of assembly gaps in the genomic region. The octamer repeats feature found in chicken *CATHB1* appeared to be unique to Galliformes (or Phasianidae), as it has only been seen in the chicken and turkey.

### Conserved synteny

Avian β-defensins cluster in a syntenic region flanked by cathepsin B (*CTSB*) and translocation associated membrane protein 2 (*TRAM2*) genes (Fig. 3). The gene order is highly conserved as CTSB, AvBD13, 12, 11, 10, 9, 8, 7, 2, 1, 3, 5, 4, 14, and TRAM2, with duplicated AvBD1 and 3 interspersed in the region between AvBD2 and 5. The strand orientations of AvBDs are also strictly maintained, with the exception of several inverted AvBD1 and 3 gene duplicates in Passeriformes. However, it should be noted that most of the genome assemblies surveyed were of draft quality with defensins found on up to 11 different scaffolds/contigs, thus there may be unrevealed lineage-specific gene rearrangements.

Similar to AvBDs, avian cathelicidins also form a conserved gene cluster, which is flanked by kelch-like family member 18 (*KLHL18*) and transforming growth factor beta regulator 4 (*TBRG4*) genes (Fig. 4). The majority of species share a conserved gene order of *KLHL18*, *CATHL2*, *CATHL3*, *CATHB1* and *TBRG4*. Exceptions include an inversion of the region containing *CATHL3* and *CATHL2* in Galliformes, and *CATHL3* and *CATHB1* being arranged in the reverse order in the common cuckoo (*Cuculus canorus*).

Such clustering of homologous genes in a tightly linked fashion is rather common for immune genes. Other well-known examples include the major histocompatibility complex (MHC), immunoglobulins (Ig), Fc receptors (FcR) and killer-cell Ig-like receptors (KIR). These immune gene families are believed to be regularly refreshed via in *cis* duplication, resulting in related genes lying next to each other in linked array in the genome [39]. It has been suggested that immune genes clustering together may be biologically significant in that it may facilitate the coordinated expression of functionally related loci, and therefore has been selectively maintained [39, 40].

### Net charge of mature peptides of β-defensins and cathelicidins

**Fig. 5:**
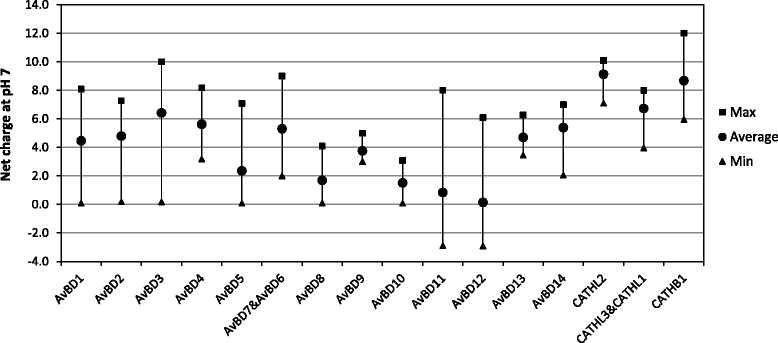
Comparison of net charges of putative avian β-defensin and cathelicidin mature peptides

Disruption of microbial membranes is a major mechanism underlying antimicrobial activity of defensins and cathelicidins [41] and it has been demonstrated that the net charge of the mature peptide directly influences its antimicrobial potency [42]. The net charge of the mature peptide ranges from −2.9 to +10.0 in avian β-defensins and +4.0 to +12.0 in cathelicidins (Fig. 5). On average, cathelicidins have higher charges than the defensins. Six defensin subfamilies (AvBD5, 8, 9, 10, 11 and 12) have an average net charge lower than +4.0, with AvBD12 showing the lowest average charge (+0.1). AvBD3 and all cathelicidin subfamilies have an average charge higher than +6.0. The low net charge of certain AvBDs indicates that they may have lower activities in terms of direct killing microbes. By contrast, the highly cationic peptides, such as AvBD3 in the emperor penguin, Adélie penguin (*Pygoscelis adeliae*) and yellow-throated sandgrouse (*Pterocles gutturalis*) with a net charge of +10.0, and CATHB1 in the kea (*Nestor notabilis*) with a net charge of +12.0, may provide valuable templates for developing new antimicrobial agents. The mature peptide sequences of all avian β-defensins and cathelicidins are provided in Additional file 4 and net charges shown in Additional file 5.

### Avian cathelicidins and the “charge balance hypothesis”

**Fig. 6:**
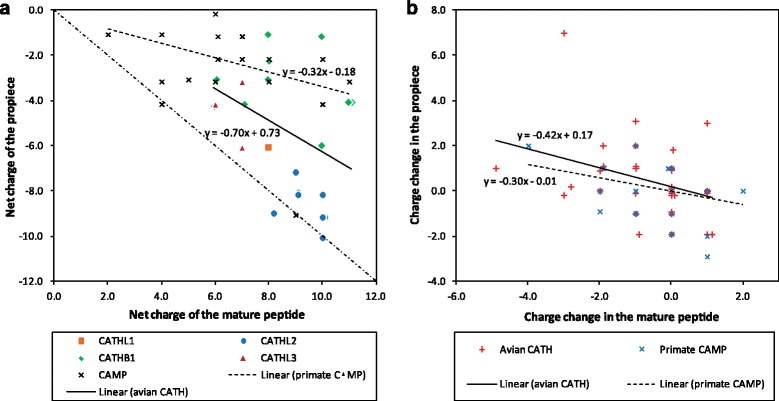
Relationship between net charges of the propiece and mature peptide in avian and primate cathelicidins. **a** Separate linear regression lines were drawn for avian (*y* = −0.70x + 0.73; *r* = −0.38; *p* = 0.03) and primate *CAMP* (*y* = −0.32x − 0.18; *r* = −0.38; *p* = 0.02) genes. **b** Changes in the net charge of the propiece and mature peptide, as inferred to have occurred during evolution based on ancestral sequence reconstruction. Separate linear regression lines were drawn for avian (*y* = −0.42x + 0.17; *r* = −0.35; *p* = 0.01) and primate (*y* = −0.30x − 0.01; *r* = −0.36; *p* = 0.01) data points

In mammalian α-defensins, Michaelson et al. [43] proposed that the anionic propiece plays a role in preventing autocytotoxicity by neutralising the cationicity of the mature peptide. The authors demonstrated a linear relationship between the net negative charge of the propiece and the positive charge of the mature peptide of seven α-defensins (two human and five rabbit genes). Hughes and Yeager [4, 44] provided further supportive evidence of this relationship (*r* = −0.742; *p* < 0.001) using 28 α-defensins from five mammalian species (mouse, rat, guinea pig, rabbit and human). This “charge balance hypothesis” is unlikely to apply to the β-defensins due to the short length of the propiece (0–7 amino acids in AvBDs). However, we explored the hypothesis in avian cathelicidins and revealed a similar, though weaker (*r* = −0.38; *p* = 0.03) association between the electrostatic charges of the propiece and mature peptides (Fig. 6a). All annotated avian cathelicidins with an intact coding sequence were used in the analysis (*n* = 24). While the mature form of all cathelicidins is highly cationic (8.51 ± 0.61), the propiece has an anionic character (−5.24 ± 1.13). The inactive form of CATHL2 before secretion has the lowest mean net charge (0.81 ± 0.62) among avian cathelicidins (overall mean = 3.27 ± 1.06), suggesting a better neutralising effect of the propiece in this subfamily. It should be noted that changes in cytoplasmic pH also affect the electrostatic charges of peptides, with higher pH resulting in lower charges (e.g. at pH 7.5, overall mean charge of presecretory avian cathelicidins = 2.79 ± 1.05). Similar level of correlation (*r* = −0.38; *p* = 0.02) was also found in 28 primate cathelicidin genes (Fig. 6a; accession numbers of primate genes listed in Additional file 6).

Further analysis was performed on reconstructed ancestral sequences to infer changes that may have occurred in avian and primate cathelicidins over evolutionary time (Fig. 6b, Additional file 7) [44]. A significant negative correlation was observed between electrostatic property changes in the propiece and mature peptide in both groups (aves: *r* = −0.35, *p* = 0.01; primate: *r* = −0.36, *p* = 0.01). When substitutions increasing peptide charge occurred in the mature cathelicidin, charge in the propiece tended to either decrease or remain unchanged, whereas when the mature peptide became less cationic, the propiece tended to become less anionic. This is highly similar to what Hughes and Yeager observed in mammalian defensins [44].

Moreover, evidence of intra-molecular amino acid residue co-evolution was detected between two pairs of sites with electrostatic properties in the propiece and mature peptide domains of CATHB1 (highlighted in Additional file 3). Residue 122L/G/E/K and 226G/K/R, and 160R/Q/P and 222E/D/G/N showed 96.9 and 80.2 % probability of having been co-evolving, respectively (residue positions are based on chicken CATHB1).

These observations indicate that, similar to mammalian α-defensins, the propiece and mature peptide of avian and primate cathelicidins may have co-evolved in such a way that amino acid substitutions in both regions are selected and accumulated to balance the charge. Further experimental evidence will be needed to validate this hypothesis and elucidate the role of cathelicidin propiece in preventing autocytotoxicity.

### Amino acid sites under selection

**Fig. 7:**
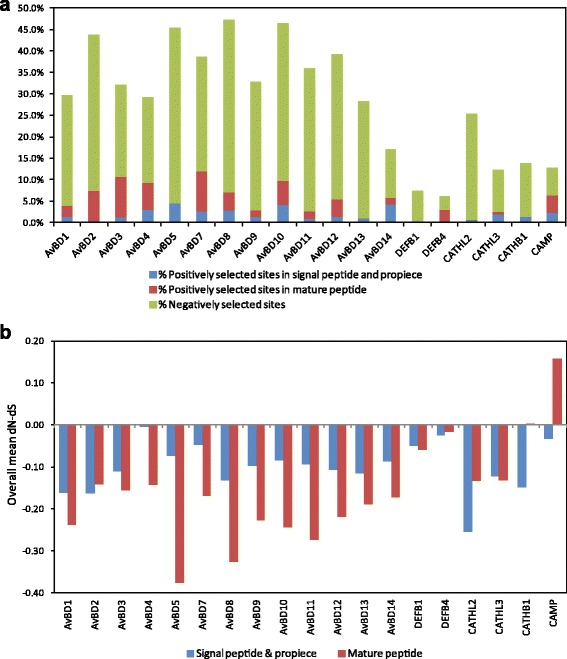
Natural selection in avian β-defensins, cathelicidins, and three primate genes (*DEFB1*, *DEFB4* and *CAMP*). **a** Proportion of codon sites under positive and negative selection in each gene; **b** Comparison between mean rates of synonymous and nonsynonymous substitutions (d_N_ − d_S_) in the signal and propiece region and mature peptide domain

Negative selection was detected in a large proportion of amino acid sites in the examined avian β-defensin (11.4–40.9 %) and cathelicidin (9.7–24.7 %) genes (Fig. 7a, Additional file 3). In AvBD1, 2, 3, 8, 9, 11, 12 and 13, the overall mean rate of nonsynonymous nucleotide substitutions (d_N_) was significantly (at 0.05 nominal level) lower than that of synonymous substitutions (d_S_) in both mature peptide and the signal domains, suggesting an overall effect of negative selection on these genes (Fig. 7b, Table 3). In the other five AvBDs significant negative selection was detected only in the mature peptide, whereas the opposite pattern was observed in the avian cathelicidin genes with only the signal and propiece domain showing significantly higher d_S_ than d_N_.

**Table 3:** Mean rates of synonymous (d_S_ ± S.E.) and nonsynonymous (d_N_ ± S.E.) nucleotide substitutions and test for overall selection in β defensin and cathelicidin genes

Gene	Signal peptide & propiece					Mature peptide				
	d_S_	d_N_	d_N_ − d_S_	Stat^b^	P^c^	d_S_	d_N_	d_N_ − d_S_	Stat^b^	P^c^
AvBD1	0.338 ± 0.061	0.175 ± 0.035	−0.162 ± 0.062	2.623	**0.005**	0.585 ± 0.073	0.347 ± 0.068	−0.237 ± 0.094	2.494	**0.007**
AvBD2	0.223 ± 0.033	0.060 ± 0.015	−0.163 ± 0.034	4.450	**0.000**	0.288 ± 0.032	0.147 ± 0.030	−0.141 ± 0.046	3.102	**0.001**
AvBD3	0.250 ± 0.052	0.139 ± 0.034	−0.111 ± 0.045	2.495	**0.007**	0.690 ± 0.048	0.534 ± 0.088	−0.155 ± 0.092	1.699	**0.046**
AvBD4	0.210 ± 0.039	0.205 ± 0.036	−0.005 ± 0.040	0.117	0.453	0.319 ± 0.061	0.176 ± 0.042	−0.143 ± 0.068	2.183	**0.016**
AvBD5	0.216 ± 0.045	0.143 ± 0.029	−0.073 ± 0.053	1.401	0.082	0.473 ± 0.055	0.098 ± 0.022	−0.376 ± 0.060	6.476	**0.000**
AvBD7	0.194 ± 0.049	0.147 ± 0.037	−0.047 ± 0.066	0.708	0.240	0.396 ± 0.057	0.227 ± 0.046	−0.169 ± 0.072	2.377	**0.010**
AvBD8	0.226 ± 0.045	0.095 ± 0.021	−0.131 ± 0.052	2.529	**0.006**	0.446 ± 0.043	0.121 ± 0.024	−0.325 ± 0.049	6.715	**0.000**
AvBD9	0.152 ± 0.038	0.053 ± 0.017	−0.098 ± 0.040	2.552	**0.006**	0.317 ± 0.061	0.090 ± 0.018	−0.227 ± 0.056	4.025	**0.000**
AvBD10	0.191 ± 0.044	0.106 ± 0.024	−0.085 ± 0.059	1.461	0.073	0.361 ± 0.051	0.117 ± 0.026	−0.244 ± 0.054	4.612	**0.000**
AvBD11	0.164 ± 0.054	0.069 ± 0.015	−0.095 ± 0.059	1.670	**0.049**	0.362 ± 0.037	0.088 ± 0.013	−0.274 ± 0.040	6.450	**0.000**
AvBD12	0.281 ± 0.048	0.171 ± 0.043	−0.108 ± 0.056	1.895	**0.030**	0.334 ± 0.053	0.115 ± 0.022	−0.219 ± 0.056	3.752	**0.000**
AvBD13	0.222 ± 0.050	0.108 ± 0.023	−0.114 ± 0.057	2.135	**0.017**	0.249 ± 0.044	0.059 ± 0.013	−0.190 ± 0.042	4.547	**0.000**
AvBD14	0.351 ± 0.071	0.263 ± 0.044	−0.087 ± 0.079	1.099	0.137	0.309 ± 0.054	0.137 ± 0.025	−0.172 ± 0.055	3.029	**0.002**
DEFB1^a^	0.072 ± 0.039	0.023 ± 0.008	−0.049 ± 0.040	1.261	0.105	0.097 ± 0.041	0.040 ± 0.020	−0.058 ± 0.039	1.467	0.072
DEFB4^a^	0.054 ± 0.034	0.029 ± 0.016	−0.025 ± 0.041	0.622	0.268	0.158 ± 0.049	0.143 ± 0.030	−0.016 ± 0.057	0.283	0.389
CATHL2	0.417 ± 0.051	0.162 ± 0.023	−0.255 ± 0.053	4.756	**0.000**	0.401 ± 0.082	0.267 ± 0.054	−0.134 ± 0.095	1.416	0.080
CATHL3	0.310 ± 0.047	0.187 ± 0.026	−0.123 ± 0.052	2.448	**0.008**	0.379 ± 0.095	0.247 ± 0.051	−0.132 ± 0.095	1.372	0.086
CATHB1	0.448 ± 0.039	0.300 ± 0.024	−0.148 ± 0.043	3.362	**0.001**	0.508 ± 0.074	0.508 ± 0.057	0.001 ± 0.090	0.006^d^	0.497^d^
CAMP^a^	0.095 ± 0.015	0.063 ± 0.008	−0.032 ± 0.017	1.852	**0.033**	0.066 ± 0.018	0.216 ± 0.039	0.157 ± 0.041	3.906^d^	**0.000** ^d^

^a^ Primate genes

^b^ Z-test statistic for purifying selection (d_S_ > d_N_)

^c^ Values of *P* < 0.05 are considered significant (in bold)

^d^ Tested for positive selection (d_S_ < d_N_)

**Fig. 8:**
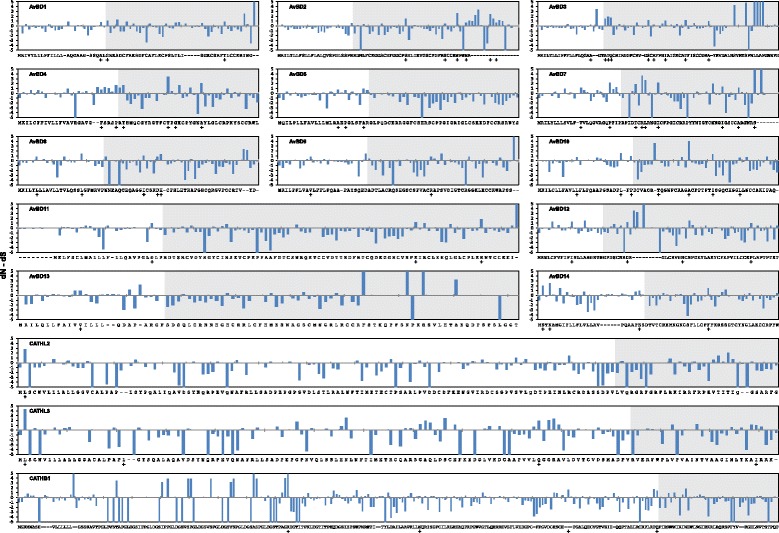
Positively selected sites in avian β-defensins and cathelicidins. Site-wise rates of synonymous and nonsynonymous substitutions (d_N_ − d_S_) calculated using FEL are shown (cutoff at 5 for large |d_N_ − d_S_|). Chicken genes are shown as reference sequence; *dashes* indicate gaps introduced during alignment with orthologues in other species; mature peptide domains are indicated by *grey* graph background. Sites under positive selection are indicated by *plus signs*

Despite the strong background of negative selection, evidence indicating specific amino acid sites subject to episodic diversifying selection was found in most studied avian genes (Fig. 8), though the effect of such selection was very weak in AvBD9, AvBD11 and AvBD13 and the three cathelicidins, with less than 4 % of total sites inferred to be positively selected. To reduce chances of false positive detections, only codon sites that were detected by multiple selection test methods were considered significant (see Methods section) [45, 46]. In the examined avian AMPs, most positively selected sites were found inside the mature peptide domain, except for AvBD5, AvBD13 and AvBD14 and all cathelicidins, in which more were found in the signal and propiece regions (Figs. 7a and 8). Within mature defensins, diversifying selection appears to mainly (68.3 % cases) affect those residues that are close to (within two residues) the conserved cysteines. For comparison, same analyses were performed on two primate β-defensin subfamilies, DEFB1 (*n* = 24) and DEFB4 (*n* = 10), and the primate cathelicidin CAMP (*n* = 28) (gene accession numbers provided in Additional file 6). Consistent with previous reports, our results suggested that positive selection has involved in driving the evolution of primate DEFB4 (coding for β-defensin 2) [47], whereas DEFB1 is highly conserved with no evidence of diversifying selection in primate lineages [48]. Taken together both positive and negative selection, AvBDs appear to have evolved under higher selective pressures and restraint, while the two examined mammalian β-defensins have evolved more neutrally in primates (Fig. 7a). Contrary to what was observed in avian cathelicidins, in primate CAMP a majority (58.3 %) of positively selected residues are located inside the mature peptide, resulting in a significantly higher overall d_N_ than d_S_ in the gene domain (Fig. 7).

Due to the important role of electrostatic charge on antimicrobial potency of the mature peptide, positively charged amino acid residues are expected to have evolved under positive selection. However, in the examined AvBDs and cathelicidins, a number of such sites showed strong negative selection or neutral results. One possible explanation is that these cationic residues may have been selectively accumulated prior to the divergence of the studied bird lineages (~114 million years ago), and then have been conserved by negative selection through the long evolutionary history due to their significant role on peptide function. Further studies to include more distant taxa (such as reptiles) will help elucidate the evolutionary dynamics of the peptides.

### Lineages subject to episodic diversifying selection

Evolutionary branches that were indicated to have experienced diversifying selection episodes at each gene are highlighted in Additional file 8. On average, 20.9 and 26.6 % of nodes over the course of evolution of AvBDs and cathelicidins, respectively, were detected with episodes of positive selection, with the highest percentage observed in AvBD7 (41.4 %) and lowest in AvBD13 (7.1 %). The later stages of evolution within avian lineages seem to have involved relatively stronger diversifying selection as compared to the more ancient branches, which is consistent with the observation that the examined gene families are generally well conserved across all avian orders and families. This result contradicts to what was previously detected in mammalian, particularly primate β-defensins, which involved more positive selection episodes in more ancient branches due to duplication and diversification of β-defensins in the early stages of mammalian evolution [5].

Several evolutionary nodes were detected to have undergone diversifying selection at multiple genes (Additional file 8). For example, AvBD5, 7, 10, 12 and 14 of Galliformes were inferred to be under diversifying selection between 84.5 and 37.9 million years before present (evolutionary time estimated based on branch length in Fig. 1), prior to the divergence between family Phasianidae (chicken and turkey) and Odontophoridae (northern bobwhite *Colinus virginianus*). Within the order Psittaciformes, AvBD3, 4 and 8 were estimated to have been subject to episodic positive selection between 79.8 and 55.3 million years ago before the divergence of the Puerto Rican parrot (*Amazona vittata*), budgerigar (*Melopsittacus undulatus*) and kea (*Nestor notabilis*), all belonging to the Psittacidae family. Similarly, positive selection appeared to have been involved in driving the diversification of AvBD1, 3, 4, 5, 8, 10 and 12 during the early radiations of Passeriformes. For each species, the number of genes indicated to have been affected by episodic diversifying selection is shown in Fig. 1.

## Conclusion

In this study, we investigated the evolution of β-defensins and cathelicidins in 53 bird species. Both gene families form a generally conserved gene cluster in avian genomes with certain genes being more prone to duplication (AvBD1, AvBD3 and AvBD7) or pseudogenisation (AvBD14) events. Intense negative selection was detected in a majority of examined gene domains, likely accounting for the conservation of certain amino acid residues that are essential for the functioning of β-defensins and cathelicidins in birds. Evidence indicated that episodic positive selection also played a role in driving the diversification of specific residues of certain antimicrobial peptides in avian evolutionary history, contributing to high variability of gene sequences and electrostatic property of the peptides. Our results also revealed that selection may have acted on cathelicidins to maintain a balanced charge between the anionic propiece and cationic mature peptide over evolutionary time. This work not only has greatly improved our understanding of the molecular evolution of these host defense peptides, but also provides a valuable resource for potential translational research and development of novel antimicrobial agents.

## Methods

### Database search and gene nomenclature

Fifty-three bird genomes were searched for β-defensin and cathelicidin genes (GenBank Assembly IDs provided in Table 1). For each genome, four steps were taken to identify genes of interest: 1) An initial search was performed on a predicted protein/CDS database with BLAST programs using chicken genes as query sequences. Hits with *E*-value <0.1 were extracted from the database, aligned to chicken sequences with ClustalW [49], and manually examined to exclude false positives. 2) Then for each gene, a profile hidden Markov model (HMM) [50] was built from a peptide sequence alignment that includes all orthologues found in the previous step. The profile HMMs were searched against the predicted protein databases with HMMER3.1 programs [51] on a GALAXY platform. Hits with both *E*-values (full sequence and best 1 domain) <1 were extracted, aligned with previously found sequences, and manually checked to confirm real homologues. 3) Sequences identified in the previous steps were then used to BLAST search the whole genome to find any genes, gene fragments, or pseudogenes that are not included in the protein/CDS database. 4) All genomic scaffolds and contigs containing β-defensin or cathelicidin genes were extracted to study the genomic organisation of these genes. In addition, sequences containing *CTSB*, *TRAM2*, *KLHL18* or *TBRG4*, the flanking genes of the β-defensin or cathelicidin gene clusters, were also extracted. Scaffolds and contigs were manually curated using Artemis [52].

Annotated sequences were named by tagging the gene name with a five-letter abbreviation as a suffix that distinguishes the species. For example, AvBD1_ACACH refers to gene *AvBD1* of the rifleman (*Acanthisittachloris*). Duplications of AvBD1 and AvBD3 were numbered 1.n and 3.n from AvBD2. Some AvBD1 and AvBD3 duplicates were found on isolated scaffolds and numbering of these defensins may not represent the actual position in the intact cluster. Previously used identification references for zebra finch duplicates [9] are included in the identification.

### Mature peptide prediction and net charge estimation

Sequence features, such as signal peptide, propiece and mature peptide, within annotated genes were speculated based on functional domains in chicken defensins [10] and cathelicidins [30, 31, 33, 34]. The net charge of mature peptide was estimated as ${\displaystyle \sum_i}{N}_i\frac{1}{1+{10}^{pH-pK{a}_i}}-{\displaystyle \sum_j}{N}_j\frac{1}{1+{10}^{pK{a}_j-pH}}$ with *N*_*i*_ and *pKa*_*i*_ being the number and pKa values of histidine (H), lysine (K) and arginine (R) residues and the N-terminus, and *N*_*j*_ and *pKa*_*j*_ the number and pKa of aspartic acid (D), glutamic acid (E), cysteine (C) and tyrosine (Y) residues and the C-terminus [53]. Lehninger’s set of pKa values were used [54] and intramolecular disulfide bond formation was taken into account in calculation.

### Evolutionary analyses

Overall phylogenetic analyses between all avian β-defensins and cathelicidins (Fig. 2) were conducted in MEGA5 with a Maximum Likelihood method [55, 56] using four discrete categories for the Gamma distribution to model evolutionary rate differences among sites and 100 bootstrap replicates to infer the level of confidence on the phylogeny (support values lower than 40 % are not shown in the consensus tree) [57].

A range of evolutionary analyses were performed on each β-defensin and cathelicidin gene via the Datamonkey webserver [58], including: 1) Negative selection sites were detected using Fixed Effect Likelihood (FEL) [59] and Fast Unconstrained Bayesian Approximation for Inferring Selection (FUBAR) [60]. 2) Individual sites under positive selection were detected using three test methods, including Mixed Effects Model of Evolution (MEME) [61], FEL, and FUBAR. Codon sites found to be significant for positive or negative selection by more than two methods (MEME *p* < 0.05, FEL *p* < 0.1, and FUBAR posterior probability >0.9) were included in the analyses [45, 46]. 3) Individual branches with episodic diversifying selection were inferred by combining results from two analyses–MEME (emprical bayes factor >20) and branch-site REL (*p* < 0.05) [62]. 4) Intramolecular co-evolution of amino acid sites in cathelicidins were detected using the Spidermonkey/Bayesian Graphical Model [63]. Only sites that are involved in electrostatic properties and have more than three branches with nonsynonymous substitutions were included in the analysis for covariation. Assessment of overall mean rates of nonsynonymous (d_N_) and synonymous (d_S_) nucleotide substitutions in the signal and propiece region and the mature peptide domain, and the significance test of overall selection were calculated in MEGA5 [55] using the Kumar model [64] and 1000 bootstrap replicates to estimate standard errors.

Ancestral cathelicidin sequences were inferred using the Maximum Likelihood method [64] under the Whelan And Goldman model [65] in MEGA5. Rates among sites were treated as a Gamma distribution using five Gamma categories.

### Ethics

No human, human data, or animal was used in this study.

## Availability of supporting data

GenBank assembly IDs of all 53 examined avian genomes are provided in Table 1. Scaffold and coordinates information of annotated genes are detailed in Additional file 1. Nucleotide sequences of all annotated genes are available in Additional file 2. Full-length amino acid sequence alignments and putative mature peptide sequence of each gene are provided in Additional files 3 and 4, respectively. GenBank accession numbers of primate genes used in analyses are listed in Additional file 6.

## Additional files

Additional file 1:
**List of annotated β-defensin and cathelicidin genes in 53 avian species with scaffold and coordinates information.** (XLSX 47 kb) Additional file 2:
**Nucleotide sequences of annotated avian β-defensin and cathelicidin genes (in fasta format).** (FAS 204 kb) Additional file 3:
**Sequence alignments of avian β-denfensins and cathelicidins.** The ruler is based on residue positions of chicken sequences. Dots represent identical residues in comparison with the chicken sequence in the top; dashes are gaps or missing data. Functionally significant conserved residues are boxed. Plus and minus signs on top of the alignment indicate sites under positive and negative selection, respectively. Letters ‘a’ and ‘b’ above the CATHB1 alignment indicate two pairs of amino acid residues with evidence of intra-molecular co-evolution. A sequence logo was generated at the bottom of the alignment to show the frequency of certain amino acid substitution at a position, indicated by the height of the letter [66]. The labelled alignments and sequence logo were produced with TEXshade [67]. (PDF 1538 kb) Additional file 4:
**Putative mature peptide sequences from annotated avian β-defensin and cathelicidin genes (in fasta format).** (FAS 42 kb) Additional file 5:
**Net charge of putative avian β-defensin and cathelicidin mature peptides.** (XLSX 30 kb) Additional file 6:
**List of primate genes used in analyses with Genbank accession numbers.** (XLSX 11 kb) Additional file 7:
**Reconstructed avian and primate ancestral cathelicidin sequences and inferred charge changes between nodes.** (PDF 299 kb) Additional file 8:
**Phylogenetic trees of avian β-defensin and cathelicidin genes.** Branches with evidence suggesting episodic diversifying selection are highlighted in red. (PDF 1444 kb)

## Abbreviations

AMP: Antimicrobial peptide
AvBD: Avian β-defensin; Abbreviations for species names are listed in Table 1
