# Supplementary material for: Evolution of the avian β-defensin and cathelicidin genes
Source: BMC Evol Biol. 2015 Sep 15;15:188. doi: 10.1186/s12862-015-0465-3 (PMC4571063; doi:10.1186/s12862-015-0465-3)

Logo legend

- X acidic
- X aliphatic
- X aliphatic (small)
- X amide
- X aromatic
- X basic
- X hydroxyl
- X imino
- X sulfur

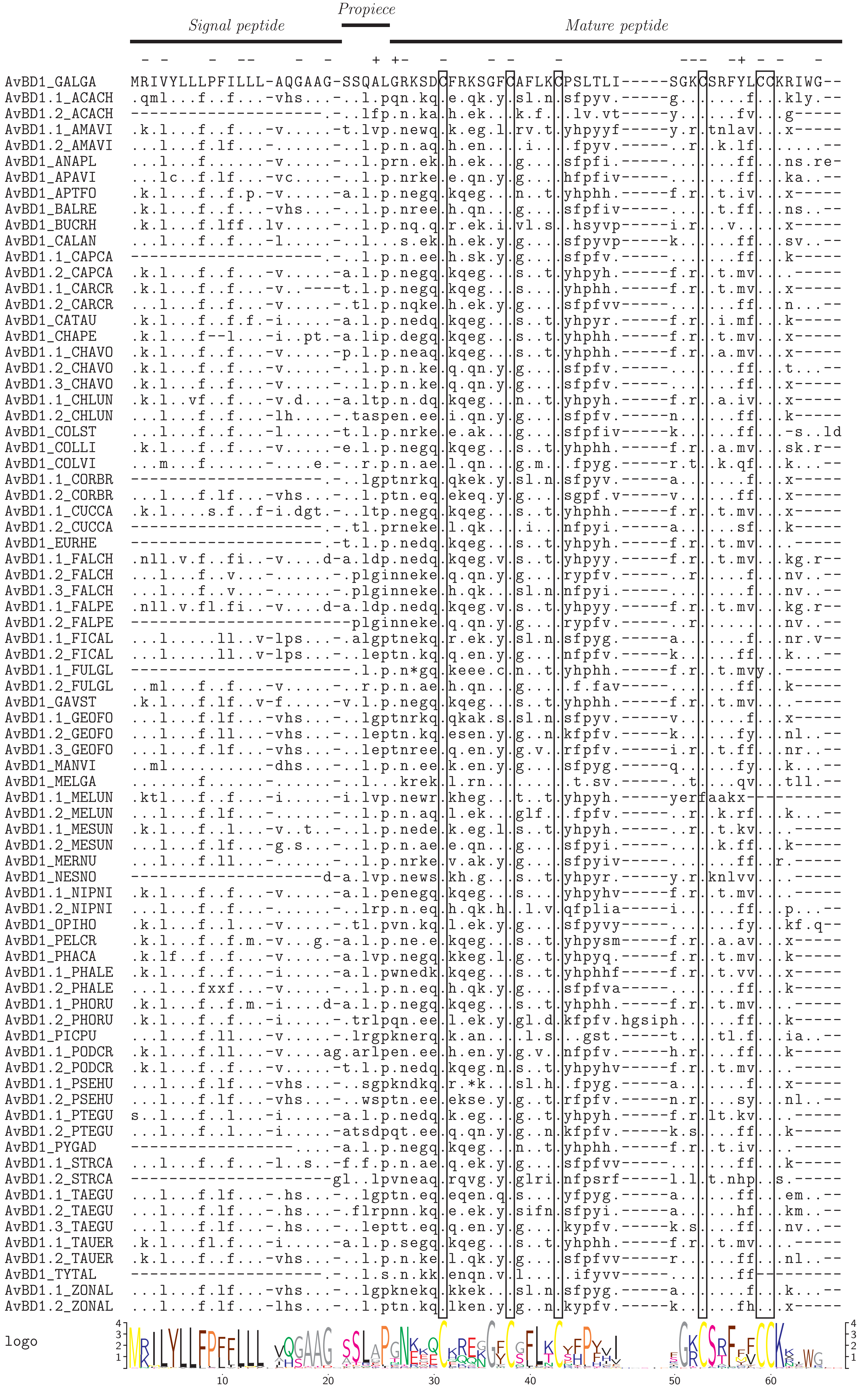
Propiece
Signal peptide
Mature peptide
AvBD1_GALGA MRIVYLLLPFILLL-AQGAAG-SSQALGRKSDCFRKSGFCAFLKCPSLTLI-----SGKCSRFYLCCKRIWG--
AvBD1.1_ACACH
AvBD1.2_ACACH
AvBD1.1_AMAVI
AvBD1.2_AMAVI
AvBD1_ANAPL
AvBD1_APAVI
AvBD1_APTFO
AvBD1_BALRE
AvBD1_BUCRH
AvBD1_CALAN
AvBD1.1_CAPCA
AvBD1.2_CAPCA
AvBD1.1_CARCR
AvBD1.2_CARCR
AvBD1_CATAU
AvBD1_CHAPE
AvBD1.1_CHAVO
AvBD1.2_CHAVO
AvBD1.3_CHAVO
AvBD1.1_CHLUN
AvBD1.2_CHLUN
AvBD1_COLST
AvBD1_COLLI
AvBD1_COLVI
AvBD1.1_CORBR
AvBD1.2_CORBR
AvBD1.1_CUCCA
AvBD1.2_CUCCA
AvBD1_EURHE
AvBD1.1_FALCH
AvBD1.2_FALCH
AvBD1.3_FALCH
AvBD1.1_FALPE
AvBD1.2_FALPE
AvBD1.1_FICAL
AvBD1.2_FICAL
AvBD1.1_FULGL
AvBD1.2_FULGL
AvBD1_GAVST
AvBD1.1_GEOFO
AvBD1.2_GEOFO
AvBD1.3_GEOFO
AvBD1_MANVI
AvBD1_MELGA
AvBD1.1_MELUN
AvBD1.2_MELUN
AvBD1.1_MESUN
AvBD1.2_MESUN
AvBD1_MERNU
AvBD1_NESNO
AvBD1.1_NIPNI
AvBD1.2_NIPNI
AvBD1_OPIHO
AvBD1_PELCR
AvBD1_PHACA
AvBD1.1_PHALE
AvBD1.2_PHALE
AvBD1.1_PHORU
AvBD1.2_PHORU
AvBD1_PICPU
AvBD1.1_PODCR
AvBD1.2_PODCR
AvBD1.1_PSEHU
AvBD1.2_PSEHU
AvBD1.1_PTEGU
AvBD1.2_PTEGU
AvBD1_PYGAD
AvBD1.1_STRCA
AvBD1.2_STRCA
AvBD1.1_TAEGU
AvBD1.2_TAEGU
AvBD1.3_TAEGU
AvBD1.1_TAUER
AvBD1.2_TAUER
AvBD1_TYTAL
AvBD1.1_ZONAL
AvBD1.2_ZONAL
logo
10
20
30
40
50
60

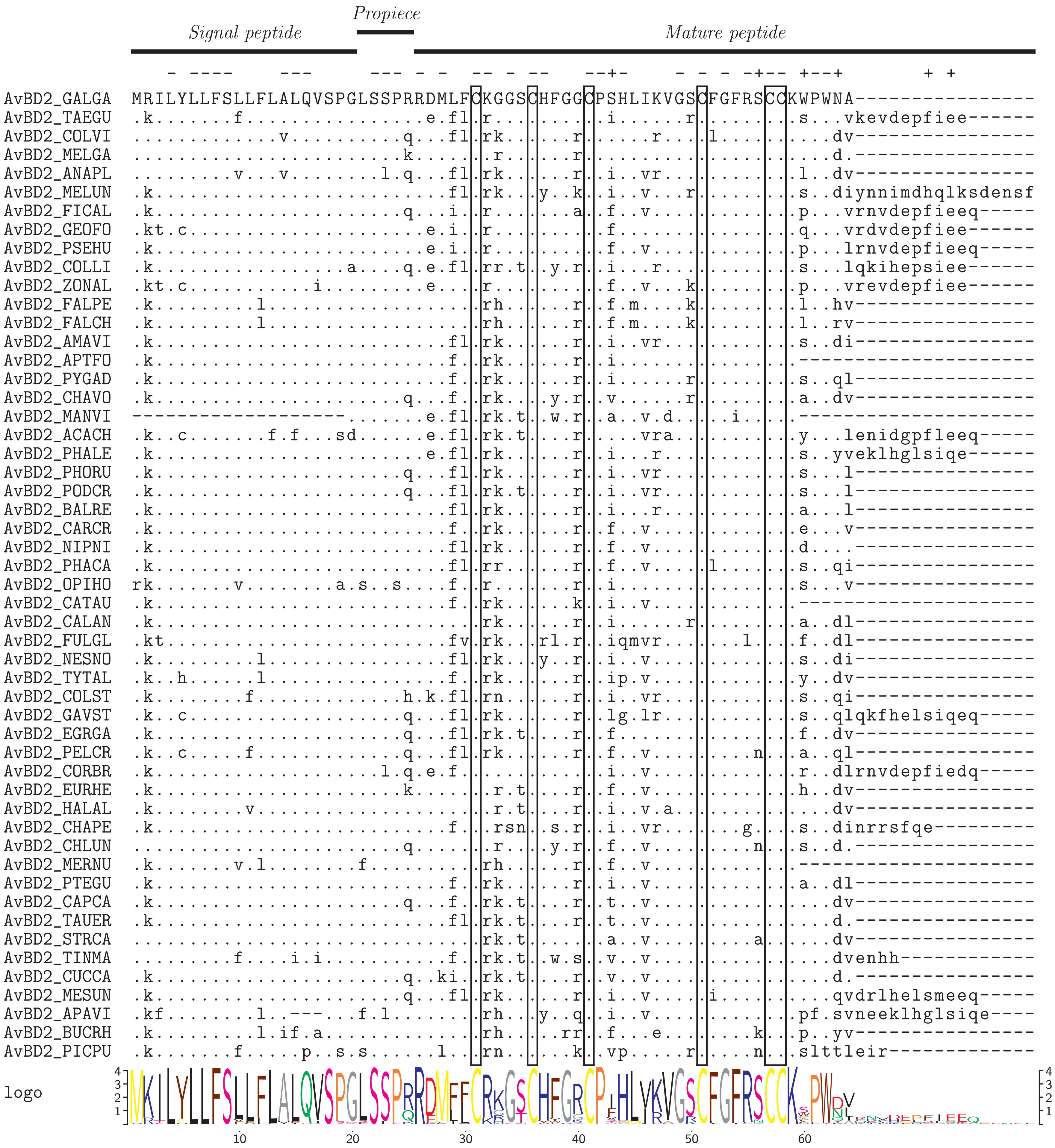
Propiece
Signal peptide
Mature peptide
AvBD2_GALGA MRILYLLFSLLFLALQVSPGLSSPRRDMLFCKGGSCHFGGCPSHLIKVGSCFGFRSCCKWPWNA
AvBD2_TAEGU
AvBD2_COLVI
AvBD2_MELGA
AvBD2_ANAPL
AvBD2_MELUN
AvBD2_FICAL
AvBD2_GEOFO
AvBD2_PSEHU
AvBD2_COLLI
AvBD2_ZONAL
AvBD2_FALPE
AvBD2_FALCH
AvBD2_AMAVI
AvBD2_APTFO
AvBD2_PYGAD
AvBD2_CHAVO
AvBD2_MANVI
AvBD2_ACACH
AvBD2_PHALE
AvBD2_PHORU
AvBD2_PODCR
AvBD2_BALRE
AvBD2_CARCR
AvBD2_NIPNI
AvBD2_PHACA
AvBD2_OPIHO
AvBD2_CATAU
AvBD2_CALAN
AvBD2_FULGL
AvBD2_NESNO
AvBD2_TYTAL
AvBD2_COLST
AvBD2_GAVST
AvBD2_EGRGA
AvBD2_PELCR
AvBD2_CORBR
AvBD2_EURHE
AvBD2_HALAL
AvBD2_CHAPE
AvBD2_CHLUN
AvBD2_MERNU
AvBD2_PTEGU
AvBD2_CAPCA
AvBD2_TAUER
AvBD2_STRCA
AvBD2_TINMA
AvBD2_CUCCA
AvBD2_MESUN
AvBD2_APAVI
AvBD2_BUCRH
AvBD2_PICPU
logo
10
20
30
40
50
60

Signal peptide — *Propiece* — *Mature peptide*

```
                   -  --  - ---  - +  - +++--   - -   +-+   +  - - +   --   +
AvBD3_GALGA     MKILYLLIPFFLLFLQGAA--GTATQCRIRGGFCRV-GSCRFPHIAIGKCATFISCCGRA-YEVDALNSVRTSPWLLAPGNNPH
AvBD3.1_ACACH   .......f.ll..lv....evrntis..r...y.tl-.g..y.trp..r.t.atv..kst-wg---------------------
AvBD3.2_ACACH   .r..c..f.....va..g.--.ktdk.ir.....vl-.g....yryv.s.talsh..klt-wg---------------------
AvBD3.3_ACACH   ---------------------.nkke.hrak...af-lk.pvlfrie...sr.ff..kn-------------------------
AvBD3.4_ACACH   ------------------------ag..qk....ty-.n..y.srp..r.tlysv..k.-------------------------
AvBD3.5_ACACH   .......f.ll..lv...seernkie..r...y.nf-.g.py.rrl..i.tassv..kx-------------------------
AvBD3_AMAVI     .......f.ll..lv....al.keek.irkk...vl-.g....ykyv.t.s..sh..kfi-wg---------------------
AvBD3.1_ANAPL   ....f..f...........--.nsvl......r.h.-...h..erh..r.sg.qa..i.t-wg---------------------
AvBD3.2_ANAPL   ....f..f..v........--.shqa..r.....tf-.r....trpv.r.s.lvp..rst-wg---------------------
AvBD3.3_ANAPL   ---------------------xsrqa..q...i.tf-ar....ttp..r.s.avp..kx-------------------------
AvBD3.4_ANAPL   ....f..f...........--.ndir.t.e..r..f-.g.q.aekq....yrivp..sx-------------------------
AvBD3.5_ANAPL   ....f..f..v........--.stlr..r...i..f-.n.l..erh..r.ss.qp..s.i-fg---------------------
AvBD3.6_ANAPL   ....f..ls........d.v-.nswl.vr...n..f-.r.q.aerq..r.sa.qp....t-wg---------------------
AvBD3.1_APAVI   ---------------------.sekr.kk....yvl-ag.fsgtti....s..ya..kkt-wg---------------------
AvBD3.2_APAVI   .......la.s..lv....--.nrqi.qrk..y.tw-...ky.ttpv.r.srlqv..ksi-wg---------------------
AvBD3_APTFO     .......f.....li.rsf--.---l..l.r...ar-.r....a.p..r.sr.vq..r.v-wg---------------------
AvBD3.1_BALRE   .......f.....lv....--.neg..kqe....sf-lt.yh..hif.r.s...v..kk-------------------------
AvBD3.2_BALRE   .......f.....lv...t--.nvl..nr...i.ty-.r..y.ski..r.sy.qp..rsi-wg---------------------
AvBD3_CAPCA     .......f.ll..li....--risil..k.r...tf-.r..y.atp..r.sr.qq..rsv-wg---------------------
AvBD3_CARCR     ---------------------.nsir..lkr.y.vs-.r..y.arp..r.sv.qq..rsv-wg---------------------
AvBD3.1_CHAPE   .......f.....li.s..--.nplr.lq...q.tf-vr..y.tr.v.r.s.vqp..isi-wg---------------------
AvBD3.2_CHAPE   ---------------------.ssfr..q...h.a.-.h..ssertl.r.s..rp..i.-------------------------
AvBD3.3_CHAPE   ---------------------.sssr..qs..r.fl-rp.hy.ek...r.sv.ri..k.-------------------------
AvBD3.1_CHAVO   .......f.....lv....--.stnr..rq....fl-.g....yrfv.v.s..sh..kki-wg---------------------
AvBD3.2_CHAVO   .......fs....mi..s.--.nsfg.tq...y.tl-.r..praasl.r.si.ya..rs.-wg---------------------
AvBD3_CHLUN     .......f.....lv....gs.ssdl..r.....tf-....y.ttp....s..rq..ksl-wg---------------------
AvBD3.1_COLLI   .......f.ll..lv.s..--.stdg.ir.k...vl-.g....ykyv.l.tk.sh..kfi-wg---------------------
AvBD3.2_COLLI   .......f.....li....--.nea...q.....ti-.r.hy.tt...r.ssyrh..r.w--v---------------------
AvBD3_COLST     .......f.....l..dd.--.neg..kqe....sf-lt.yh.dhif.r.s..mv..k--------------------------
AvBD3_COLVI     ..................e--.nfi...r...v..s-.g..irqrt.....a..p..rx-------------------------
AvBD3.1_CORBR   .r.....f.l...lv....--.-----ir.....a.-.g....ykfa.v.s.lsh..kp-------------------------
AvBD3.2_CORBR   .r.....f.l...lv....--.-----ir.....a.-.g....ykfa.v.s.lsh..kp-------------------------
AvBD3.3_CORBR   ....f..f.li..lv....--.ikna.qll..y.af-wk..p.trr..n.sa.ti..k.v-rg---------------------
AvBD3.4_CORBR   ....f..f.li..lv....--.sel...h.r.y.sf-.r.ip.arp..r.taqti..kg-------------------------
AvBD3.5_CORBR   ....f..f.li..lv....--.i-fr..q.r.y.sf-qr..r.dwpl.r.taqtv..kg-------------------------
AvBD3.6_CORBR   ....f..f.li..lv....a-.-----.q...y.ty-.r.q..arp..r.tlysv..k.-------------------------
AvBD3.1_CUCCA   .r.....f.l...l.....--.kldr..r.......a-tg.prgrpp..r..nglr..rx-------------------------
AvBD3.2_CUCCA   .r.m...lv.s..lf....--.kldr..r.......a-tg.prgrpp..r..nglr..rx-------------------------
AvBD3_EGRGA     .......f.....lv....--.nvl..i......sl-.g.qi.gtt..r.sg.ql..k.-------------------------
AvBD3_EURHE     .r.....f.....lv....--.nfls..g.rqy.af-.r..y.a.pv.r.sasrq..tnv-wr---------------------
AvBD3_FALCH     .r.....f..v..la....--.srsp..rn..h.vl-.g.l..lktm.s.si.r...klv-wg---------------------
AvBD3_FALPE     tr.....f..v..la....--.srsp..rn..h.vl-.g.l..lktm.s.si.r...klv-wg---------------------
AvBD3.1_FICAL   ....f..f.li..lv....--.nssa..r.....tf-.r..y.ttpv.s.salkv..kx-------------------------
AvBD3.2_FICAL   ....f..f.li..lv....--.ikna.qll..y.ty-wk..pstrp....sa.ti..k.v-rg---------------------
AvBD3.3_FICAL   ....f..f.li..lv....--.sssr..r.....sf-dg.ss.s.p..r.srvsv..kx-------------------------
AvBD3.4_FICAL   .r..f..f.ll..lv....a-.khdk.ir...i.vl-.g....yryv.v.s.lsh..kpv-wg---------------------
AvBD3.5_FICAL   ---------------------.gile..r.r.y.sf-.r.ip.a.p..r.tgqti..rgr-wv---------------------
AvBD3.6_FICAL   ....f..f.li..lv...sa-.-----.q...y.ty-.r.n..arp..r.tlysv..k.-------------------------
AvBD3_FULGL     .......f.....lv....--.ssf..tq.....tf-wr.pysttr..r.sgsrl..t.i-wg---------------------
AvBD3_GAVST     .r.....f.l...lv....--.nvdr..*qk...vl-.g....ykfv.v.s..sh..kli-wg---------------------
AvBD3.1_GEOFO   ....f..l.l...lgl...--.n.ee..lkr...lr-dy.ptgthra.t..pg.l..r.g-eslwv------------------
AvBD3.2_GEOFO   ....f..f.li..lv....--.diit..r...s.tf-.r..y.tr.v.r.ssvqv..isl-wv---------------------
AvBD3.3_GEOFO   ....f..f.li..lv....--.nena..r...s.tf-....y.ttp..r.sr.kv..rsl-wv---------------------
AvBD3.4_GEOFO   .r..f..f.l...lvp...a-.khak.irq....a.-.g....ykyv.v.s.lsh..kli-wg---------------------
AvBD3.5_GEOFO   ....f..f.li..lvr...--.n.g...rl.....f-lg.ss.akp..r.sansl..kin-wv---------------------
AvBD3.6_GEOFO   ....f..f.li..lv....--.srvv.lklw...sr-r..pigtrvl.r..gefl..kx-------------------------
AvBD3.7_GEOFO   ....f..f.li..lv..st--.s.lr.kqqr.y.sf-.r.vp.arp..r.tgqti..rgl-wg---------------------
AvBD3.8_GEOFO   ....f..f.ll..lvr...--.sri..ke.....ss-vr..p.lrt..r.sdmav..r.m-wc---------------------
AvBD3.9_GEOFO   ....f..f.li..lv...sa-.-----.r...y.ty-.r.n..arp..r.tlysv..k.-------------------------
AvBD3_HALAL     .......f.....li.s..--.npfr..rq..h.tl-.r.hy.stpv.i.srshi..rt-------------------------
AvBD3_LEPDI     ..t....f.....lv....--.neg..kke....sf-lt.yh..hvf.r.s...v..kk-------------------------
AvBD3.1_MANVI   .......f.ll..lv....--.skfr.k.n..d..f-.k.pygtkp..r.s..rv..kst-wg---------------------
AvBD3.2_MANVI   .......f.ll..lv....etqsefk.lls..d..f-ai.aygtkp..r.s..ka..kx-------------------------
AvBD3.3_MANVI   .......f.ll..lv....awdntlk.kst....af-vk.psgtr.a...tssty..kx-------------------------
AvBD3.4_MANVI   .rl....f..l..lv....a-.ktdk.ik.....vf-.g....ykyv.t.tlvsh..kli-wg---------------------
AvBD3.5_MANVI   ---------------------.svhl..hqr.i.vf-.r.plgtrpv.r.taqtl..r.-------------------------
AvBD3.6_MANVI   ---------------------.npdk.irqr.i.tf-.g..p.tvp..q.sedtf..k.-------------------------
AvBD3.7_MANVI   .......f.ll..lv....a-.-----.rq..y.ty-.r.n..arp..r.slysv..k.-------------------------
AVBD3_MELGA     .......f........s..--..pi.........f-...........a......p.....-.g.....f...l..khlc-----
AvBD3_MELUN     .r.....f.ll..lv....al.keek.lrkk.v.if-.g....ykyv.s.s..rh..kli-wg---------------------
AvBD3_MERNU     .......fav...lv....--.stlg.sq...y.tf-.r..y.ars..r.sv.r...r.-------------------------
AvBD3.1_MESUN   .......f.....li....--.stlg.tq.......f-.r.qp.t.sl.r.si.rh..k.i-wv---------------------
AvBD3.2_MESUN   .......f.....li....--.s..g..l...y.sl-ar..p.avp..r.sr.ad..kki-wv---------------------
AvBD3.3_MESUN   ....sf.fail.vish...----vmr.gy..t..fp-.t.plgnayl.v.rsgh...kw-------------------------
```

logo

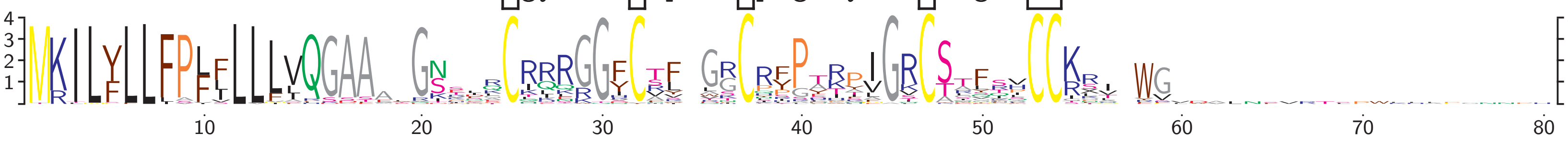

Propiece

Signal peptide — *Mature peptide*

```
                   - --  - ---  - +  - +++--   - -    +-+   +  - - +   --  +
AvBD3_GALGA     MKILYLLIPFFLLFLQGAA--GTATQCRIRGGFCRV-GSCRFPHIAIGKCATFISCCGRA-YEVDALNSVRTSPWLLAPGNNPH
AvBD3_NESNO     .......f.ll..lv....--.keq..irkk...vl-.g....ykyv.t.s..sh..kfi-wg---------------------
AvBD3_NIPNI     .......f.....lv....--.spfr.iq...y.sf-.d..y.ars..r.sr.qq..kni-wg---------------------
AvBD3_OPIHO     .r.....f.....lv..s.--.ntl..klqr...tl-wp..h.y.s..i.srlhh..r.-------------------------
AvBD3_PELCR     .......f.....li..t.--.npll.vq.r...ty-qr..y.svp..r.sh.qy..ksv-wg---------------------
AvBD3_PHACA     .r..f..fsl...lvl...--entd..ik.k...vf-.g....ykyv.s.tr.sh..km-------------------------
AvBD3.1_PHALE   .r..p..call..m...t.--.spqd.er.....sh-r..ppgigr..l.seqdf..r.-------------------------
AvBD3.2_PHALE   .......f.....li..p---.si-r..q...y.vl-.n..y.ttp..r.sr.hr..r.-------------------------
AvBD3.3_PHALE   .r.....fl....la....--.ntdr..rq....ll-.g....ykfm.i.s..rh..kkriwg---------------------
AvBD3.1_PHORU   .......f....qli....--.n.dr..rqr...vl-.g....ykfv.v.s..kh..ktv-wg---------------------
AvbD3.2_PHORU   -------------------------l..r.....tf-.....y.ats..r.sr.r...k.-------------------------
AvBD3_PICPU     .......f..li.lf....--.sslg.tq.....af-rr.pssavt..r.si.rk..rsn-wg---------------------
AvBD3_PODCR     r......f.....li....--.nlf...q.....sf-.r..y.stpv.r.srlrp..k.-------------------------
AvBD3.1_PSEHU   ....f..f.li..la....--.ne.s.welh...tr-nt.pegtsfv.r.spglv..k.g-kslwv------------------
AvBD3.2_PSEHU   ....f..f.li..la....--.s..s.wkqr...tr-ri.prgtsfv.r.spgla..kx-------------------------
AvBD3.3_PSEHU   ....f..f.li..la....--.sssa..r.....tf-.g..y.ttpv.r.savsv..ksl-wg---------------------
AvBD3.4_PSEHU   ....f..f.l...lips..a-.khek.ir.....a.-.g....ykfv.v.s.lsh..kpv-wg---------------------
AvBD3.5_PSEHU   ....f..f.li..la....--.sele.kr.r.y.yf-.k.vl.arq..r.tgqtv..rgl-wg---------------------
AvBD3.6_PSEHU   ....f..fhli..la...s--.sii...e.r.i.sf-vr..p.lrs....talav..rx-------------------------
AvBD3.7_PSEHU   ....f..f.li..la....a-.------q...y.ty-.r.n..arp..r.tlysv..k.-------------------------
AvBD3_PTEGU     .r.....f.....li..te--.svrg.ar...v.vf-.r....rrp..r.sglra..ksv-wg---------------------
AvBD3_PYGAD     .......f.....li.s..--.gfgl..v.....t--rr....arpf.r.sr.lq..r.v-wg---------------------
AvBD3.1_STRCA   ---------------------.s.ss..r.....vf-.g....tkn....s..vh..k.-------------------------
AvBD3.2_STRCA   ---------------------.s.ss..r.....tf-.v.i..y.sn...saisl..k.-------------------------
AvBD3.1_TAEGU   .r..f..f.l...lvhs..--.khdk.vr.....a.-.g....ykyv.v.salsh..kpi-wg---------------------
AvBD3.2_TAEGU   ....f..f.ll..lv....--.s.vt.wrkq...aw-r..prgarvv.i.sprml..kx-------------------------
AvBD3.3_TAEGU   ....f..f.li..lv....--.npll..q.....tf-.g....stp..r.savqt..ksv-wg---------------------
AvBD3.4_TAEGU   ....f..f.li..lv....--.npll.kr...y.tl-.i..hgatpv.r.tpyrv..lgg-wv---------------------
AvBD3.5_TAEGU   ....f..f.li..lv....--.npd...rlr...fr-r..prgsapv.r..rg.a..kiv-wg---------------------
AvBD3.6_TAEGU   ....f..f.li..lv....--.ssar..r.....sf-dg.ss.skp.....savsv..ksr-wv---------------------
AvBD3.7_TAEGU   ....f..f.li..lv....--.npll..q.....tf-.g....stp..r.savqt..ksh-wv---------------------
AvBD3.8_TAEGU   ....f..f.ll..lv....--.s.vt.wrkq...aw-r..prgthvv.i.spgml..k.l-kp---------------------
AvBD3.9_TAEGU   ....f..f.ll..lv...sa-.------q...y.ty-.r.n..arp..r.tlysv..r.-------------------------
AvBD3.1_TAUER   .......f.....la.s..--.ntdr.ir.....vl-.i....ykfa.v.savsh..kti-wg---------------------
AvBD3.2_TAUER   .......f.....li....--ast.g..r.r.v.vf-.a....lrp..r.srlq...ksf-wg---------------------
AvBD3_TINMA     .r.....f.....l.....g-..srs..qk....kf-qn.y..d.q..t.si.rk..ksl-rg---------------------
AvBD3_TYTAL     .......f.....li.s..--.sssg..r.....ki-...ty.ttp..r.sr.rt..k.-------------------------
AvBD3.1_ZONAL   ..f.f..l.li..lv...e--.n.da..r.r...yr-r..frgtrlv.r.saslv..rx-------------------------
AvBD3.2_ZONAL   ....f..f.lif.li....--.n.gr..ll.....f-lg.ss.akp..r.sansl..kin-wv---------------------
AvBD3.3_ZONAL   ....fp.f.li..lf...s--.niit..rk....ti-.r..y.tr.v.r.savep..isw-g----------------------
AvBD3.4_ZONAL   ---------------------.nedk..lkr...lr-hy.ptgthra.t..pglv..rx-------------------------
AvBD3.5_ZONAL   ....f..f.li..lv...t--.nena..r...v.tff....dlikpvqr.p..kv..kx-------------------------
AVBD3.6_ZONAL   ....f..f.li..lv....--.diit..rk..s.sf-lp..y.tr.v.r.ssvqv..ix-------------------------
AvBD3.7_ZONAL   ....f..l.li..lvh...--.ssiv.k?h....sr-r..ppgthha.r.svslf..rx-------------------------
AvBD3.8_ZONAL   .r..f..f.l...lv....--.khdk.ir.....a.-.g....ykyv.v.s.lsh..kiv-wg---------------------
AvBD3.9_ZONAL   ---------------------.n.vl..rir...sw-r..prgtslv.r.spslv..rx-------------------------
AvBD3.10_ZONAL  ....f..f.li..lv....--.n.lv..rq....sr-r..pygtrfv.r..geyv..rx-------------------------
AvBD3.11_ZONAL  ..f.f..f.li..lv....--.n.lg.wr.....sw-h..s.strfv.i..gesf..kx-------------------------
AvBD3.12_ZONAL  ....f..f.li..lv....--.selr.kqqr.y.sf-.r.vp.arp..r.tgqti..rgl-wg---------------------
AvBD3.13_ZONAL  ....f..fsll..lvr...--.sti..nq.....ss-vr.hp.lrt..r.sdmsv..kx-------------------------
AvBD3.14_ZONAL  ....f..f.li..lvr.g.sa.-----.k.....ty-.r.n..arp..r.tlysv..k.-------------------------
```

logo

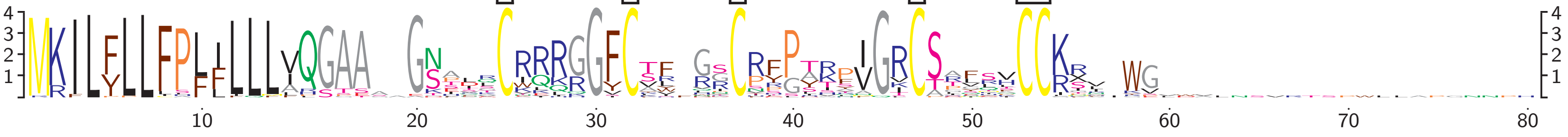

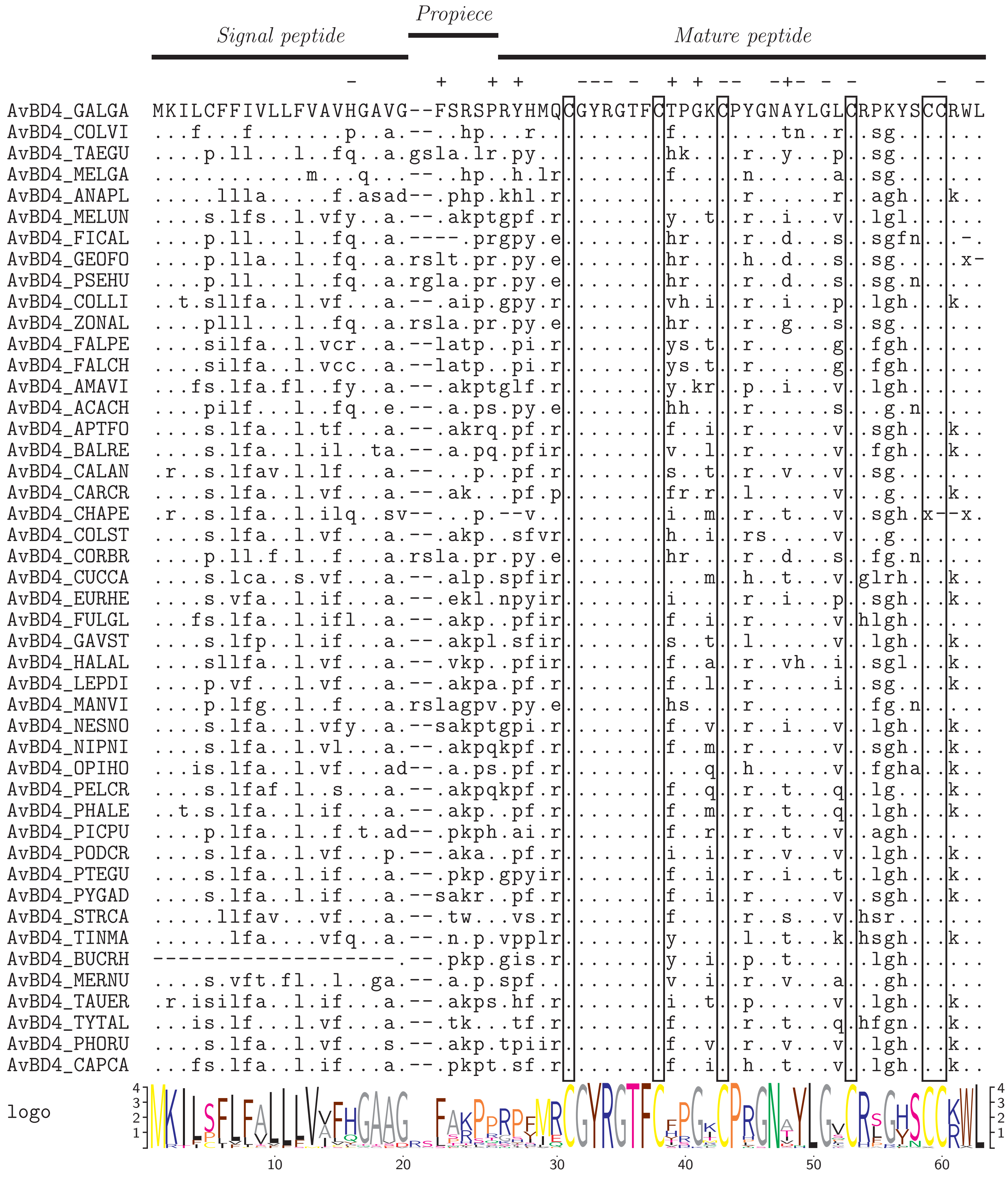
Propiece
Signal peptide
Mature peptide
AvBD4_GALGA MKILCFFIVLLFVAVHGAVG--FSRSPRYHMQCGYRGTFCTPGKCPYGNAYLGLCRPKYSCCRWL
AvBD4_COLVI
AvBD4_TAEGU
AvBD4_MELGA
AvBD4_ANAPL
AvBD4_MELUN
AvBD4_FICAL
AvBD4_GEOFO
AvBD4_PSEHU
AvBD4_COLLI
AvBD4_ZONAL
AvBD4_FALPE
AvBD4_FALCH
AvBD4_AMAVI
AvBD4_ACACH
AvBD4_APTFO
AvBD4_BALRE
AvBD4_CALAN
AvBD4_CARCR
AvBD4_CHAPE
AvBD4_COLST
AvBD4_CORBR
AvBD4_CUCCA
AvBD4_EURHE
AvBD4_FULGL
AvBD4_GAVST
AvBD4_HALAL
AvBD4_LEPDI
AvBD4_MANVI
AvBD4_NESNO
AvBD4_NIPNI
AvBD4_OPIHO
AvBD4_PELCR
AvBD4_PHALE
AvBD4_PICPU
AvBD4_PODCR
AvBD4_PTEGU
AvBD4_PYGAD
AvBD4_STRCA
AvBD4_TINMA
AvBD4_BUCRH
AvBD4_MERNU
AvBD4_TAUER
AvBD4_TYTAL
AvBD4_PHORU
AvBD4_CAPCA
logo
10
20
30
40
50
60

```
                                        Propiece
                  Signal peptide                        Mature peptide

               -  -    -    + +-  -+      -  --- --- ------ - -  -  - --- - - 
AvBD5_GALGA MQILPLLFAVLLLMLRAEPGLSFARGLPQDCERRGGFCSHRSCPPGIGRIGLCSKEDFCCRSRWYS
AvBD5_TAEGU .r...v.c.l.....qvvt..apv.asa..........................eqe....m...p
AVBD5_MELGA ...............qg.....l.q.s....................s......ekv.........
AvBD5_COLVI .r..............g.....l...s........................i..e.v........p
AvBD5_ANAPL .r.....l.......q.aa...p..s.................................r...p
AvBD5_MELUN .r.....c.l...v.qgaa.ktlsw.s.............g........v....ehe........p
AvBD5_FICAL .r...v.c.l.....qgvt...pv.asa.e.........q.........v....eqe....m...p
AvBD5_GEOFO .r...v.c.l.....qgvt...pvgasa.e........................ere....m...p
AvBD5_PSEHU .r...v.c.l......gvt...pv.asa..........................eqe....m...p
AvBD5_COLLI .r.....c.l..v..qgaa...l...s....q.......rg........v.i..ere........p
AvBD5_ZONAL .r...v.c.l...v.qgvt...pvgasa.e..........h.............ere....m...p
AvBD5_FALPE .r.....c.lff...qgaa..plt..s......q..............s...i..eq.........p
AvBD5_FALCH .r.....c.lff...qgaa..plt..s......h..............s...i..eq.........p
AvBD5_AMAVI .r.....c.l.....qgaa.ktls..s.............g........v....ehe........p
AvBD5_APTFO .r.....c.l.....qgaa...l.................g........v.i..eq.....-----
AvBD5_PYGAD .r.....c.l.....qgaa...l.................g........v.i..eq.....r...p
AvBD5_NIPNI .r.....c.f.....qgaa...l.................g........v.i..eqv....r...p
AvBD5_CATAU .r.....c.l..mlqq--a...l...s............rg..........i..eq.....r...p
AvBD5_CHAVO .r.....c.l.....qgaa...rt..s.......................v.i..eq.........p
AvBD5_PODCR .rv....c.l...ifqgaa...l...s...........----x......v.i..eq.........p
AvBD5_PHORU .r.....c.l.....qgaa...l...s.............g........v.i..eq..........
AvBD5_GAVST .r...v.c.l.....qgaa...lt..s............lg........v.i..ek.....r...p
AvBD5_HALAL .r.....c.l.....qgaa...l...s.............g......s.v.i..eq.....r...p
AvBD5_FULGL .r.....c.l.....qgaa...l...s.......r.....g..........i..eq.....r...p
AvBD5_BALRE .r.....c.l.....qgaa...l...s........................i..eq.....i...p
AvBD5_EURHE .......c.l....iqgat...l...s......h.......k...l..s...i..eq.....i...p
AvBD5_APAVI .r.....c.l.....qglv.p.l...t.............k......s.v.i..eq.........p
AvBD5_BUCRH .r.....c.l.....qgaa...lp.................k......s...i..eq.........p
AvBD5_PHALE .r.....c.l.....qgta...l...s............................eq.....r...p
AvBD5_PICPU .r.....c.-.....qgaa...l.p....h..........k.............eq.........p
AvBD5_CHLUN .r...v.c.l.....q--e....p..m.............g..........i..eh.....i...p
AvBD5_MESUN .....a.c.l.....qgat...lg.....a..........g........v.i..eq.........p
AvBD5_LEPDI .r.....c.l.....qgaa...lt..s.............g........v.v..er.........p
AvBD5_TAUER .rm....c.l.....qgaa...l...s.e..........lg......s.v.i..ek.....r...p
AvBD5_PELCR .r.....c.l..v..qkaa...l...s.............g......s.v.i..eq.........p
AvBD5_OPIHO .l....fc.l...--qgaa...ltw.s.............g..........i..eqel...r...p
AvBD5_CARCR .......c.l.....qgaa...l...s............pg........v.i..eq.....r...p
AvBD5_PTEGU .r...v.c.l....vqgaa...l...t............rg........v.i..ere....r...p
AvBD5_NESNO .r.....c.l.f...qgaa.ktlsl.s.............g...l....v....ehe........p
AvBD5_CAPCA .r.....c......fqgaa...lt..s....k........g..........i..aq..........
AvBD5_COLST .r.....s.l...v.qgaa...l...s............p..............eh.........p
AvBD5_CORBR .rl..v.c.l.....qgvr...pg.asa......................v....eqe....m...p
AvBD5_GEOFO .r...v.c.l.....qgvt...pvgasa.e........................ere....m...p
AvBD5_MANVI .r...v.cvl.....lgvt...l...sh.e.........yq........v....eqe....m...p
AvBD5_ACACH .r..sv.c.l......gvt...l.q.sr.e....................v....eq.....v...p
AvBD5_CUCCA .r...v.c.l..v..qgaa...lt..t.............g........v.v..eq.....t...p
AvBD5_EGRGA -------------------...l...s....q.......eg......n.v.i..eka........p
AvBD5_PHACA -------------------x..lpp......v.......qg...l..s.v.i..eq.l...x----
AvBD5_STRCA .rv....c.-.....hgag.f.ls..t............rg...l..t.v.i..lq.....r...p
AvBD5_TINMA .r.....c.-...i.hgaa.f.ls..t.e..q.......ng...l..v......lr.....m...p
AvBD5_CHAPE .r.....c.l...i.qela.v.l...s.gt.k......yfk...l.ft...i..mhmm........
AvBD5_CALAN .r..l..c.l...v.q.la...l...s..e........p.........v.i..eh.....x----
AvBD5_MERNU .r.....c.l...ifqgaa...l...s....q.......pg...l....v.t..qh.....r...p
```

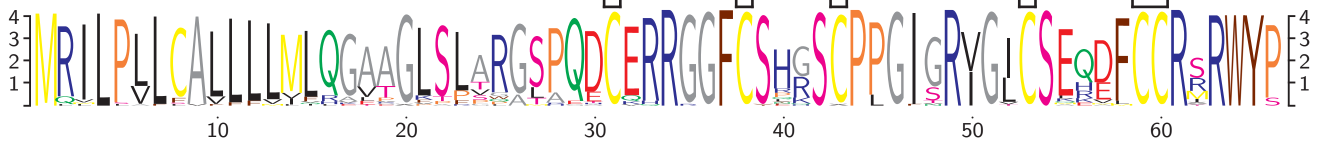

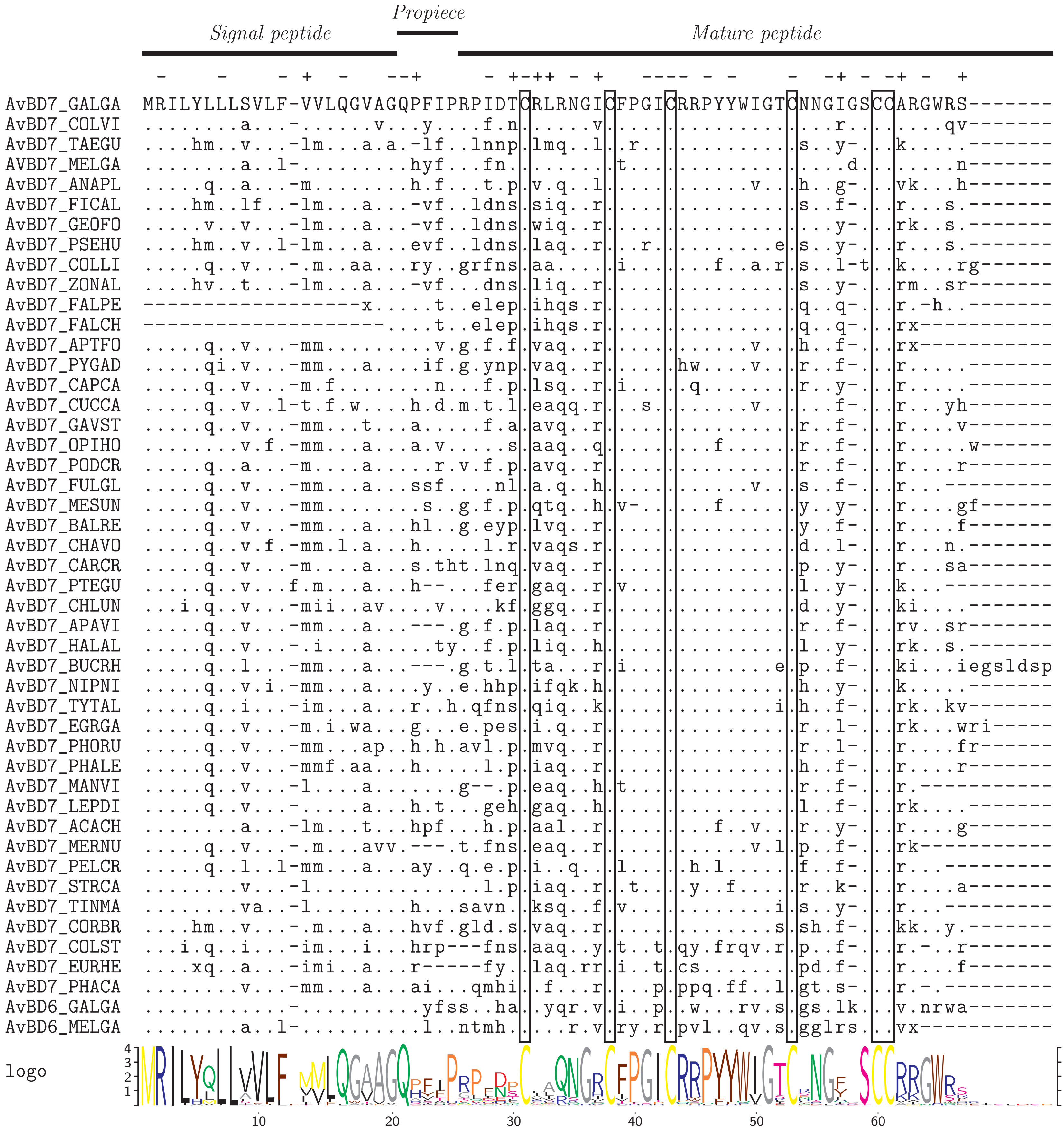
Propiece
Signal peptide
Mature peptide
AvBD7_GALGA
AvBD7_COLVI
AvBD7_TAEGU
AVBD7_MELGA
AvBD7_ANAPL
AvBD7_FICAL
AvBD7_GEOFO
AvBD7_PSEHU
AvBD7_COLLI
AvBD7_ZONAL
AvBD7_FALPE
AvBD7_FALCH
AvBD7_APTFO
AvBD7_PYGAD
AvBD7_CAPCA
AvBD7_CUCCA
AvBD7_GAVST
AvBD7_OPIHO
AvBD7_PODCR
AvBD7_FULGL
AvBD7_MESUN
AvBD7_BALRE
AvBD7_CHAVO
AvBD7_CARCR
AvBD7_PTEGU
AvBD7_CHLUN
AvBD7_APAVI
AvBD7_HALAL
AvBD7_BUCRH
AvBD7_NIPNI
AvBD7_TYTAL
AvBD7_EGRGA
AvBD7_PHORU
AvBD7_PHALE
AvBD7_MANVI
AvBD7_LEPDI
AvBD7_ACACH
AvBD7_MERNU
AvBD7_PELCR
AvBD7_STRCA
AvBD7_TINMA
AvBD7_CORBR
AvBD7_COLST
AvBD7_EURHE
AvBD7_PHACA
AvBD6_GALGA
AvBD6_MELGA
logo
10
20
30
40
50
60

```
                          Propiece
              Signal peptide          Mature peptide

               -  +    --      +   - - -  ---  --+- -++ -    -- -- -  -------   --
AvBD8_GALGA  MKILYLLLAVLLTVLQSSLGFMRVPNNEAQCEQAGGICSKDH-CFHLHTRAFGHCQRGVPCCRTV--YD-
AvBD8_TAEGU  ...fsfff....lm..gis..l.a..t.v..r....v..s.r-.ppp...p..r..q.i.......--..-
AvBD8_MELGA  ......f.....i.....v.................................-..........r..qa.......--.n-
AvBD8_COLVI  ......f.....i.....s................s.....-..p.......r...............------
AvBD8_ANAPL  ...i..ff....l..........a...av..k....t..t..-.pppn.....r.kq.i.......--..-
AvBD8_MELUN  ....a.ff..f.lm..gt.....a...dv..k....t..t.l-.plpnm.p..c.............a.--..-
AvBD8_FICAL  ...fs.ff.i..-m..gts..l.a.ht.v..r....v..sh.-.ppp...p.....q.i......s.--..-
AvBD8_GEOFO  ...fsfff....li..gts..l.a.ht.v..r....v..shr-.ppp...p.....q.i.......--..-
AvBD8_PSEHU  ...fafff....lm..gts..l.a..t.g..r.......r..-.pqp.s.p..r..q.i.......--..-
AvBD8_COLLI  ....f.ffp...l...gt.....a...ne..k....t.fa..-.plpn..p..r..qa........--..-
AvBD8_ZONAL  ...fsfff....lm..gts..l.a.st.v..r....v..spr-.psp...p..s..q.i.......--..-
AvBD8_FALPE  ...fs.f.....lm..g.s....a...hv..k....t..a..-.plpn..f..r..q.........--..-
AvBD8_FALCH  ...fs.f.....lm..g.s....a...hv..k....t..a..-.plpn..f..r..q.........--..-
AvBD8_AMAVI  ....s.ff.lfflm..gt.....a.h.dv..k....t..t.l-.plpnm.p..r..q.......a.--..-
AvBD8_APTFO  ...fs.ff....lm..gt.....a...dv..k....t..t..-.plpn..s..r.............-----
AvBD8_PYGAD  ...fs.ff....lm..gts....a...dv..k....t..t..-.plpn..s..r.............--..-
AvBD8_CATAU  -------------------....a.s.dv..k....t..tg.-.plp.m.s..r..q..........-----
AvBD8_MERNU  -------------------....a.s.dv..k....a.ftg.-.pppn..s..r..q..........-----
AvBD8_TYTAL  .r.fs.ff..v.lma.gt.....a.s.dv..k....t..t..-.plpsm.s..r..q.........--..-
AvBD8_PELCR  ...fs.ff.l..lm..gt.....a...dv..k.s..t..p..-.plptm.s..r..q.........--..q
AvBD8_BALRE  ...fs.ff....lm..gt.....a...dv..k....t..t..-.plpn..s..r..q..........-----
AvBD8_EGRGA  ...fs..f....lm..gt.....a...dv..k....t..t.r-.plpnm.s.....q.........--..-
AvBD8_FULGL  ...f..ff....lm..gtv....a...dv..k....t..t..-.plpnm.s..r..q.........--..-
AvBD8_PHACA  ...fs.ff....lm..gt.....a.g.dv..k....t..t..-.plpn..s.....q.........--..-
AvBD8_NIPNI  ...fs.ff....l...ga.....a...dv..k....t..te.-.nlpn..s..r..q.........--..-
AvBD8_PHALE  ...fs.ff....-...gt.........dv..k....t..tkst.plpn..s..r..q..........-----
AvBD8_EURHE  ...fa.ff....lm..gt.....a..tdv..k....t..s..-.plpnm.s..r..q.........--..-
AvBD8_CARCR  ...fs.f.va..l...gt.....a...dv..k....t..t..-.pl.nm.s.....q.........--..-
AvBD8_GAVST  ...fs.ff....l...gt.....a...dv..k....t..tk.-.plpnm.s..r..q.........--..-
AvBD8_PHORU  ...fsfff....l...gt.....a...dv..k....t..tt.-.plpn..s..r..q.........--..-
AvBD8_HALAL  ...fs.ffs...l...gt.....a.s.dv..k....t..t..-.plpnm.s....tq.........ai..-
AvBD8_CAPCA  ...fs.ff....l...gts....a...dv..k....t..t..-.plpn..s..r..q.........ei..-
AvBD8_TAUER  ...fs.ff...pl...gt.....a.s.dv..k....t..a..-.plpnm.s..r..q..........-----
AvBD8_OPIHO  ...fs.ffv...lmi.gts....a...dl..k....t..t..-.plpn..s..r..e.........--..-
AvBD8_MESUN  ...fp.ff.i..l...gt.....a...av..k....t..t.l-.plpn..s..r..q.......a.--..-
AvBD8_CHAVO  ...fs.ffv...la..gt.....a...dm..k....t..p..-.plpnm.s..r..q.........--..-
AvBD8_CHLUN  .t.fs.ffv...l...dts....a...dv..k....t..t..-.plhn..s..r.............--..-
AvBD8_CUCCA  ...fs.fi....l...gt.....a.y..v.......t..tn.-.plpg..p..r..q.........--..-
AvBD8_PODCR  ...fs.ff....l...gts....a...dv..k....t..ts.-.plpn..s..r.............--..-
AvBD8_MANVI  -------------------....a....v..k....v..t..-.pppn..s..r..............-----
AvBD8_ACACH  ...fs.fc....lm..ats....a...hv..k....v.at..-.pvpn..s..r..q.........--..-
AvBD8_STRCA  ...s.fff....l....t.....a.d.dv..k....s..m.r-.plr-.....r..k.i.......--..-
AvBD8_APAVI  ...fs.ffv...iap.gts....a.s.dv..k.......t.s-.plpn..s..r..q.........--..-
AvBD8_LEPDI  ...fsicf....l...gt.....a.s.dv...l...t..tg.-.pppn..p..r..q.........an..-
AvBD8_CHAPE  ...fs.ff....l...gt.....a....v..k....t..t..-.plpk.sp..r..q.........--..-
AvBD8_PTEGU  ...fs.cf.l..sm..g......a...dv..k.......t.r-.plpna.s..r..q.........--..-
AvBD8_NESNO  ...fs.ff..f.lm..gt.....a...nv..k....t..t.l-.plpsm.p..r..q..f...a.--..-
AvBD8_PICPU  ...fs.fi....l...gt.....a.s.qv..k....t..s..-.plpd..s..r..q.......a.--..-
AvBD8_BUCRH  ...fs.ffv...l...gts....a.g..v..t....t..t.r-.plpn..s..r..q.........--..-
AvBD8_COLST  ...fsfffv...l...gts....a.s.a...k....t..in.-.plpkm.s..r..q.........--..-
AvBD8_CORBR  ...fs.ff....lm..gts..l.a.st.v..r....v..s.r-.ppp...p.....q.i.......--..-
AvBD8_CALAN  ...fsiff....l...gt.....a...ne..k.......ttp-.plpt..p..t..q.........--..-
```

logo

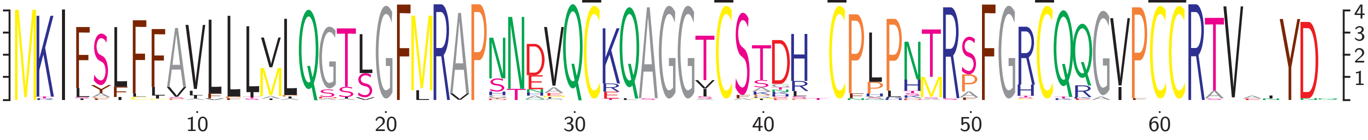

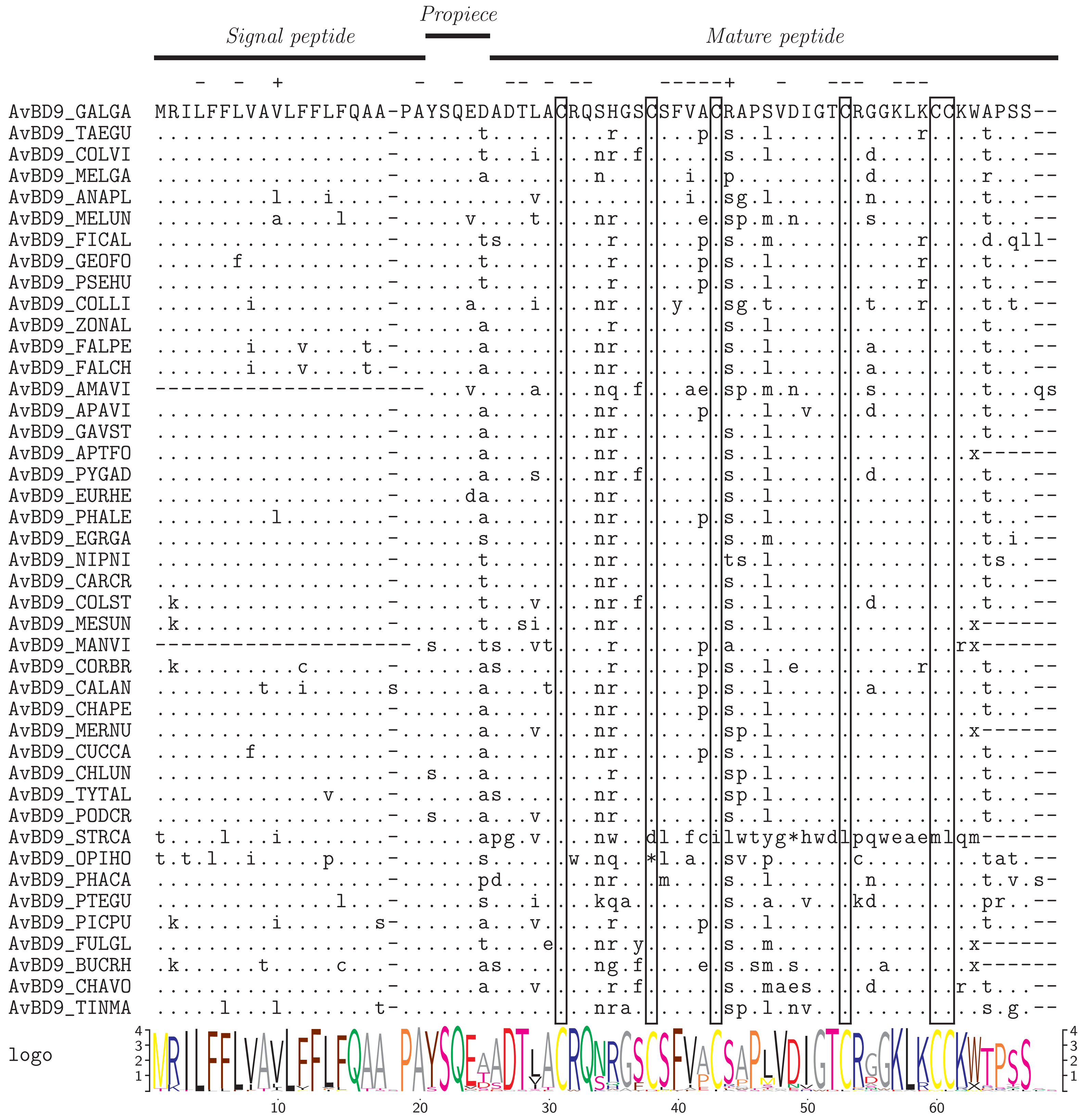
Propiece
Signal peptide
Mature peptide
- - + - - -- - -- -----+ - --- ---
AvBD9_GALGA MRILFFLVAVLFFLFQAA-PAYSQEDADTLACRQSHGSCSFVACRAPSVDIGTCRGGKLKCCKWAPSS--
AvBD9_TAEGU ....-......t.........r......p.s..l...........r....t...--
AvBD9_COLVI ....-......t...i....nr.f......s..l.......d........t...--
AvBD9_MELGA ....-......a........n......i..p..........d........r...--
AvBD9_ANAPL ....l...i....-..........v...........i..sg.l.......n........t...--
AvBD9_MELUN ....a....l...-.....v....t....nr......e.sp.m.n.....s........t...--
AvBD9_FICAL ....-......ts.........r......p.s..m...........r....d.qll-
AvBD9_GEOFO ......f...........-......t.........r......p.s..l...........r....t...--
AvBD9_PSEHU ....-......t.........r......p.s..l...........r....t...--
AvBD9_COLLI ....i..........-.....a....i....nr....y...sg.t.......t...r....t.t.--
AvBD9_ZONAL ....-......a.........r........s..l.................t...--
AvBD9_FALPE ....i...v....t.-......a........nr........s..l.......a........t...--
AvBD9_FALCH ....i...v....t.-......a........nr........s..l.......a........t...--
AvBD9_AMAVI ---------------------...v....a....nq.f..ae.sp.m.n.....s........t...qs
AvBD9_APAVI ....-......a........nr......p....l..v....d........t...--
AvBD9_GAVST ....-......a........nr........s..l.................t...--
AvBD9_APTFO ....-......a........nr........s..l................x------
AvBD9_PYGAD ....-......a...s....nr.f......s..l.......d........t...--
AvBD9_EURHE ....-.....da........nr........s..l.................t...--
AvBD9_PHALE ....l........-......a........nr......p.s..l.................t...--
AvBD9_EGRGA ....-......s........nr........s..m.................t.i.--
AvBD9_NIPNI ....-......t........nr........ts.l.................ts..--
AvBD9_CARCR ....-......t........nr........s..l.................t...--
AvBD9_COLST .k................-......t...v....nr.f......s..l.......d........t...--
AvBD9_MESUN .k................-......t..si....nr........s..l................x------
AvBD9_MANVI --------------------.s...ts..vt....r......p.a.................rx------
AvBD9_CORBR .k.........c......-......as........r......p.s..l.e.........r....t...--
AvBD9_CALAN ....t..i......s......a....t...nr......p.s..l.......a........t...--
AvBD9_CHAPE ....-......a........nr......p.s..l.................t...--
AvBD9_MERNU ....-......a...v....nr........sp.l................x------
AvBD9_CUCCA ....f..........-......a........nr......p.s..l.................t...--
AvBD9_CHLUN ....-..s...a.........r........sp.l.................t...--
AvBD9_TYTAL ....v....-......as.......nr........sp.l.................t...--
AvBD9_PODCR ....-..s...a...v....nr........s..l.................t...--
AvBD9_STRCA t....l...i........-......apg.v....nw..dl.fcilwtyg*hwdlpqweaemlqm------
AvBD9_OPIHO t.t.l..i.....p....-......s......w.nq..*l.a..sv.p......c.........tat.--
AvBD9_PHACA ....-......pd.......nr...m....s..l.......n........t.v.s-
AvBD9_PTEGU ....l...-......s...i....kqa.......s..a..v...kd........pr..--
AvBD9_PICPU .k.......i.......s-......a...v.....r......p.s..l.................t...--
AvBD9_FULGL ....-......t....e...nr.y......s..m................x------
AvBD9_BUCRH .k......t.....c...-......as.......ng.f....e.s.sm.s......a.......x------
AvBD9_CHAVO ....-......a...v.....r.f......s..maes....d......r.t...--
AvBD9_TINMA .....l...l.......t-...............nra.......sp.l.nv.............s.g.--
logo
4
3
2
1
10
20
30
40
50
60

```
                                    Propiece
                Signal peptide                          Mature peptide

                 -  --   +        ----+  + -- -  + --  - -+ ---- +--- - -+  ---
AvBD10_GALGA  MKILCLLFAVLLFLFQAAPGSADPL-FPDTVACR-TQGNFCRAGACPPTFTISGQCHGGLLNCCAKIPAQ-
AvBD10_TAEGU  ....y.....f.l....ts......-.a...e..-s..r...........aat.t.........s..l.e-
AvBD10_MELGA  ....y....................-........-n..................................-
AvBD10_COLVI  ....y............ts..t...-.....e..-r..................p..........x-----
AvBD10_ANAPL  .r..y......fl..........ap-.a..a...-s..............aa..s......k..s..l.e-
AvBD10_MELUN  .r..yv....v.l....ss......-....te..-n........s......v..s......r..srtv.e-
AvBD10_FICAL  ....y.....f.l....ts......-.a...e..-a..k...........aat.t.........s..l.e-
AvBD10_GEOFO  ....y.....f.l....ts......-.a...e..na..k.....p......at.t....t....s..s.e-
AvBD10_PSEHU  ..v.y.....f.l....ts......-ya...e..-s..k...........aat.t....i....s.vl.e-
AvBD10_COLLI  .r..y....-vfl...........i-....ae..-s.......a.......v..s......v..s..lvk-
AvBD10_ZONAL  ....y.....f.l....ts......-.a..t...sa..s...........aat.t.....m...sr.s.e-
AvBD10_FALPE  .r..yv.....fl.l..vs.....i-.a..ae..-s...............a..s......k..s..l.e-
AvBD10_FALCH  .r..yv.....fl.l..vs.....i-.a..ae..-s...............a..s......k..s..l.e-
AvBD10_AMAVI  .r.vyv....v.l....ss......-....te.k-n......v.t......v..s......r..s..l.e-
AvBD10_MANVI  ....y.....f.li...ts......-....ae..-g..k.....q......a..t..n.v....sx-----
AvBD10_ACACH  .r..ym....f.....vtsv....p-aa..l...-gk.k..hf.d..a...a-------------------
AvBD10_CORBR  ....y.....f.l....ts......-.a...e..-s..k.....v.....aat.t.........s.vl.e-
AvBD10_CHAPE  .r..y.....v.l............-.v..me.k-s........p......a..s....amk..s..s.e-
AvBD10_APAVI  ..f.y....fvfl............-ha..ae.k-s......f.......avt.t.qa.t....s..t.k-
AvBD10_CUCCA  .r..y....ivfl............-.....e..-s......v.s......v..s..s...k..s..s.e-
AvBD10_PTEGU  .r..y....ivfl.........e..-.a..ae..-a........p.....aa..t......r..s..lpe-
AvBD10_CHLUN  .r..y.....vfl....t......i-.a..ae..-s........p.........s......k..s..lpe-
AvBD10_PICPU  .r..y.....vfl....s.......-.a..ae..-s........s......a..s......k..s..t.e-
AvBD10_TYTAL  .r..y.....ifl............-.a..ae..-s...............t..s.....mk..s.tw.e-
AvBD10_APTFO  .r..y......fl...........i-.a..ae..-s..............aa..s......k..sx-----
AvBD10_PYGAD  .r..y......fl............-.a..ae..-s..............aa..s......k..s..l.e-
AvBD10_NIPNI  ....y...................i-.a..ae..-s..............aa..s......k..s..l.k-
AvBD10_PELCR  .r..........l...........i-.a..te..-s..............aa..t......k..s..l.e-
AvBD10_FULGL  .r..y.......l............-.a..ae..-s........m.....aa..s......k..s..l.e-
AvBD10_PHACA  .r..y.......l...........i-.e..aq..-s..............aa..s......k..s..l.e-
AvBD10_CATAU  .r..y......fl....g......f-.a..ae..-s...............a..s......k..sx-----
AvBD10_HALAL  .r..y......fl...........i-.a..ae..-s......v.......aa..s......k..s..l.e-
AvBD10_PHORU  ....y......fl...........i-.a..a...-s..............aa..s......k..s..l.e-
AvBD10_BALRE  .r..y.....vfl...........t-.v..a...-s........p......v..s......k..s..l.e-
AvBD10_PODCR  ....y......fl...........i-.a..a...-se.....v........t..s......k..s..l.k-
AvBD10_CHAVO  .r..y.....vfl...........i-.a..te..-s...............a..s......k..s..l.k-
AvBD10_GAVST  .r..y....ivfl...........i-.a..ae..-s........s.....aa..s......k..s..l.k-
AvBD10_EURHE  .r..y.....vfl..........qi-.a..ad..-s........t......v..s......k..sx-----
AvBD10_PHALE  .r..y.....vfl...........i-.a..a...-s........p.....av..s......k..s..l.e-
AvBD10_BUCRH  .rf.y..v..fvl...........i-....ae..-n........i.....aa..s......k..s.lies-
AvBD10_CARCR  .r..y......fl...........i-.....e..-s........p.....aa..s......k..s..l.e-
AvBD10_CAPCA  .r..y....ivfl............-....ae..-s........t.....av..s......k..s.vv.e-
AvBD10_MERNU  .r..y.....vfl............-.a..ae..-s..............sv..s......k...x-----
AvBD10_TAUER  .r..y.....vfl............-.a..ae..-.........t.....ev..s......k..s..lpv-
AvBD10_COLST  .r..y.....ifl............-.a..ad.k-s......v.i.........s......k..s..lpe-
AvBD10_LEPDI  .r..y.....vfl...........i-.a..ae..-s......v.s......v..s..a...k..s..l.e-
AvBD10_NESNO  .r..yv..t.a.i....ts.....i-....ae..-n........p......v..s......k..sr.v.e-
AvBD10_MESUN  ....y.....v.l....ts.....i-....ae..-s........t.....aa..s......k..s..l.k-
AvBD10_OPIHO  .r..y.....v.l...........v-ya..ae..-s.......vf......v..s......k....lkiec
AvBD10_CALAN  .r..y..l..vfl...........fp.a..ae..-s......v.s...s..v..p......k..s.leets
AvBD10_EGRGA  .r..y......vl............-.a..ae..-s........e.....aa..s......k..s..l.k-
AvBD10_STRCA  .r..y......fl.......n..lt-.a..ae.k-s........p.........s......k..s..ip.-
AvBD10_TINMA  .r..y......fl....t..yg.vt-.a...e.k-s......lts.....sp..s......k..kx-----
```

logo

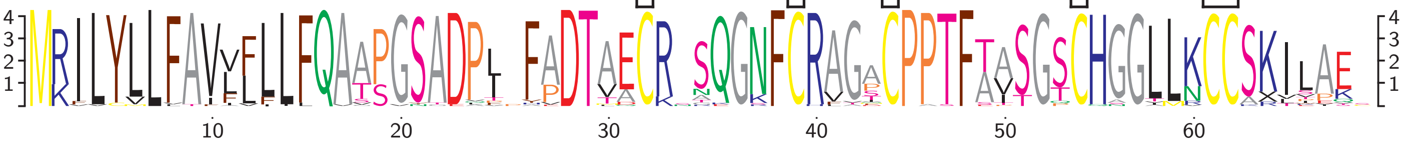

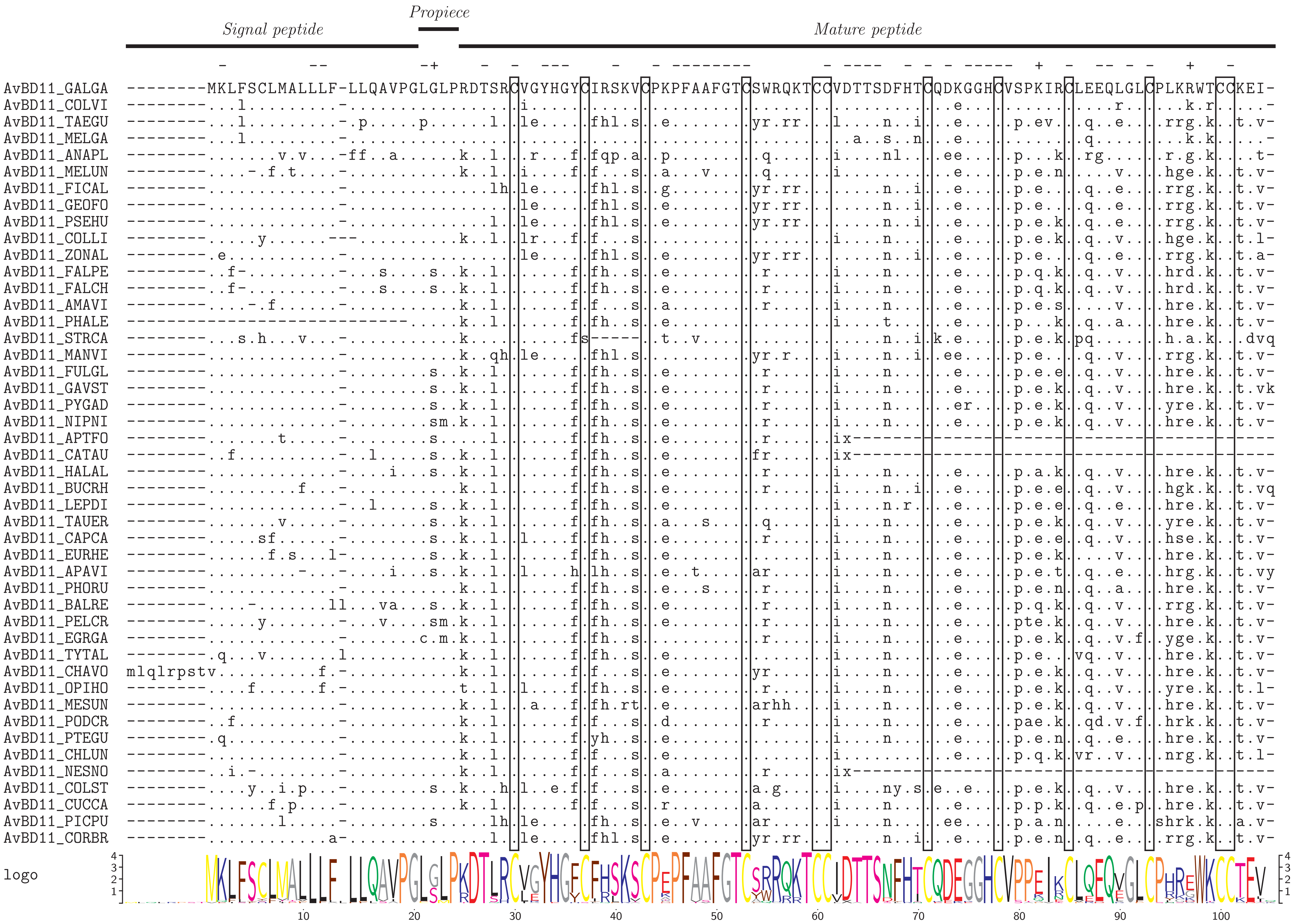

Signal peptide
Propiece
Mature peptide
AvBD11_GALGA --------MKLFSCLMALLLF-LLQAVPGLGLPRDTSRCVGYHGYCIRSKVCPKPFAAFGTCSWRQKTCCVDTTSDFHTCQDKGGHCVSPKIRCLEEQLGLCPLKRWTCCKEI-
AvBD11_COLVI
AvBD11_TAEGU
AvBD11_MELGA
AvBD11_ANAPL
AvBD11_MELUN
AvBD11_FICAL
AvBD11_GEOFO
AvBD11_PSEHU
AvBD11_COLLI
AvBD11_ZONAL
AvBD11_FALPE
AvBD11_FALCH
AvBD11_AMAVI
AvBD11_PHALE
AvBD11_STRCA
AvBD11_MANVI
AvBD11_FULGL
AvBD11_GAVST
AvBD11_PYGAD
AvBD11_NIPNI
AvBD11_APTFO
AvBD11_CATAU
AvBD11_HALAL
AvBD11_BUCRH
AvBD11_LEPDI
AvBD11_TAUER
AvBD11_CAPCA
AvBD11_EURHE
AvBD11_APAVI
AvBD11_PHORU
AvBD11_BALRE
AvBD11_PELCR
AvBD11_EGRGA
AvBD11_TYTAL
AvBD11_CHAVO
AvBD11_OPIHO
AvBD11_MESUN
AvBD11_PODCR
AvBD11_PTEGU
AvBD11_CHLUN
AvBD11_NESNO
AvBD11_COLST
AvBD11_CUCCA
AvBD11_PICPU
AvBD11_CORBR
logo
10
20
30
40
50
60
70
80
90
100

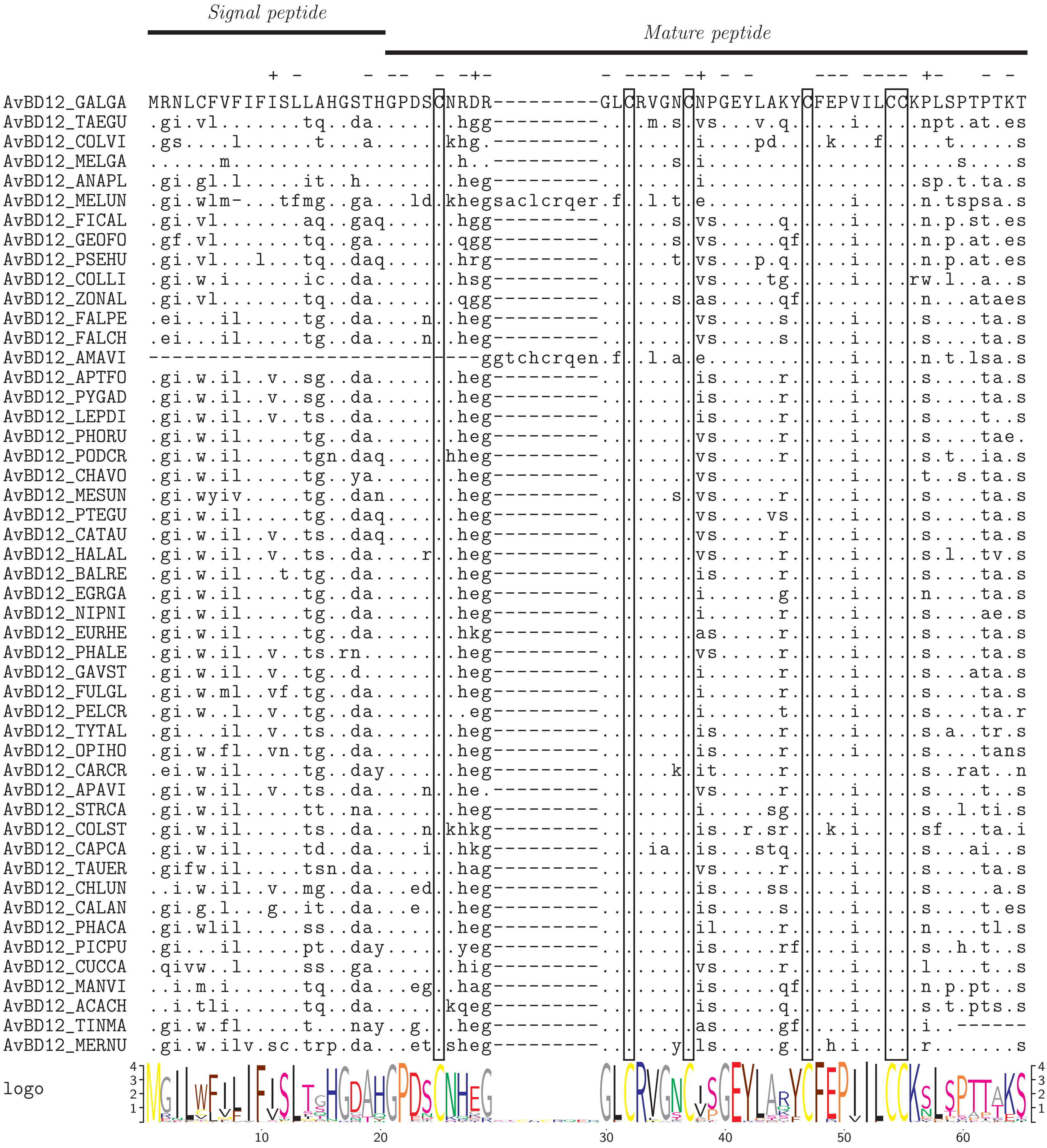

Signal peptide
Mature peptide
AvBD12_GALGA
AvBD12_TAEGU
AvBD12_COLVI
AvBD12_MELGA
AvBD12_ANAPL
AvBD12_MELUN
AvBD12_FICAL
AvBD12_GEOFO
AvBD12_PSEHU
AvBD12_COLLI
AvBD12_ZONAL
AvBD12_FALPE
AvBD12_FALCH
AvBD12_AMAVI
AvBD12_APTFO
AvBD12_PYGAD
AvBD12_LEPDI
AvBD12_PHORU
AvBD12_PODCR
AvBD12_CHAVO
AvBD12_MESUN
AvBD12_PTEGU
AvBD12_CATAU
AvBD12_HALAL
AvBD12_BALRE
AvBD12_EGRGA
AvBD12_NIPNI
AvBD12_EURHE
AvBD12_PHALE
AvBD12_GAVST
AvBD12_FULGL
AvBD12_PELCR
AvBD12_TYTAL
AvBD12_OPIHO
AvBD12_CARCR
AvBD12_APAVI
AvBD12_STRCA
AvBD12_COLST
AvBD12_CAPCA
AvBD12_TAUER
AvBD12_CHLUN
AvBD12_CALAN
AvBD12_PHACA
AvBD12_PICPU
AvBD12_CUCCA
AvBD12_MANVI
AvBD12_ACACH
AvBD12_TINMA
AvBD12_MERNU
MRNLCFVFIFISLLAHGSTHGPDSCNRDR----------GLCRVGNCNPGEYLAKYCFEPVILCCKPLSPTPTKT
logo
10
20
30
40
50
60

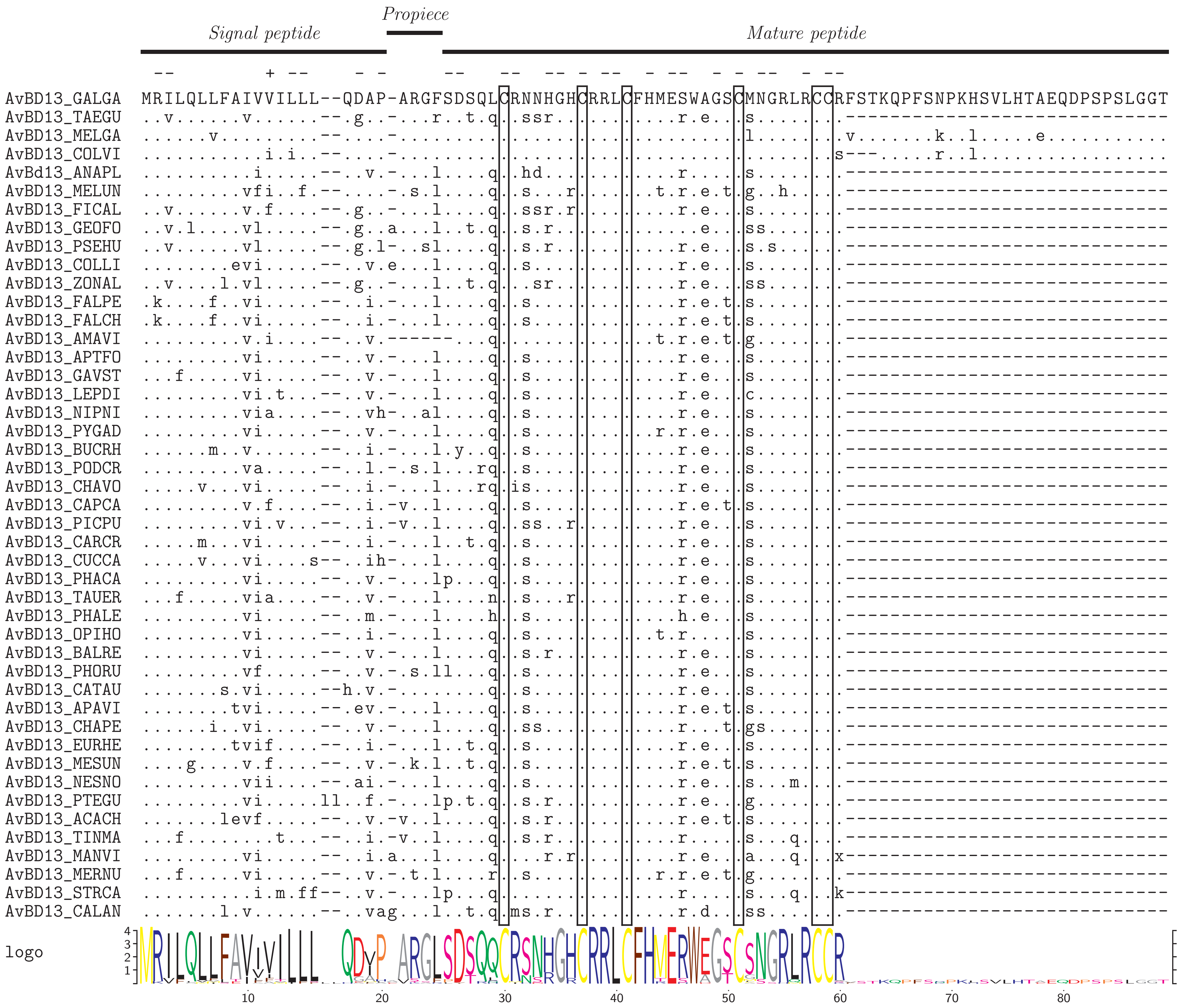

Signal peptide
Propiece
Mature peptide
AvBD13_GALGA MRILQLLFAIVVILLL--QDAP-ARGFSDSQLCRNNHGHCRRLCFHMESWAGSCMNGRLRCCRFSTKQPFSNPKHSVLHTAEQDPSPSLGGT
AvBD13_TAEGU
AvBD13_MELGA
AvBD13_COLVI
AvBd13_ANAPL
AvBD13_MELUN
AvBD13_FICAL
AvBD13_GEOFO
AvBD13_PSEHU
AvBD13_COLLI
AvBD13_ZONAL
AvBD13_FALPE
AvBD13_FALCH
AvBD13_AMAVI
AvBD13_APTFO
AvBD13_GAVST
AvBD13_LEPDI
AvBD13_NIPNI
AvBD13_PYGAD
AvBD13_BUCRH
AvBD13_PODCR
AvBD13_CHAVO
AvBD13_CAPCA
AvBD13_PICPU
AvBD13_CARCR
AvBD13_CUCCA
AvBD13_PHACA
AvBD13_TAUER
AvBD13_PHALE
AvBD13_OPIHO
AvBD13_BALRE
AvBD13_PHORU
AvBD13_CATAU
AvBD13_APAVI
AvBD13_CHAPE
AvBD13_EURHE
AvBD13_MESUN
AvBD13_NESNO
AvBD13_PTEGU
AvBD13_ACACH
AvBD13_TINMA
AvBD13_MANVI
AvBD13_MERNU
AvBD13_STRCA
AvBD13_CALAN
logo
10 20 30 40 50 60 70 80

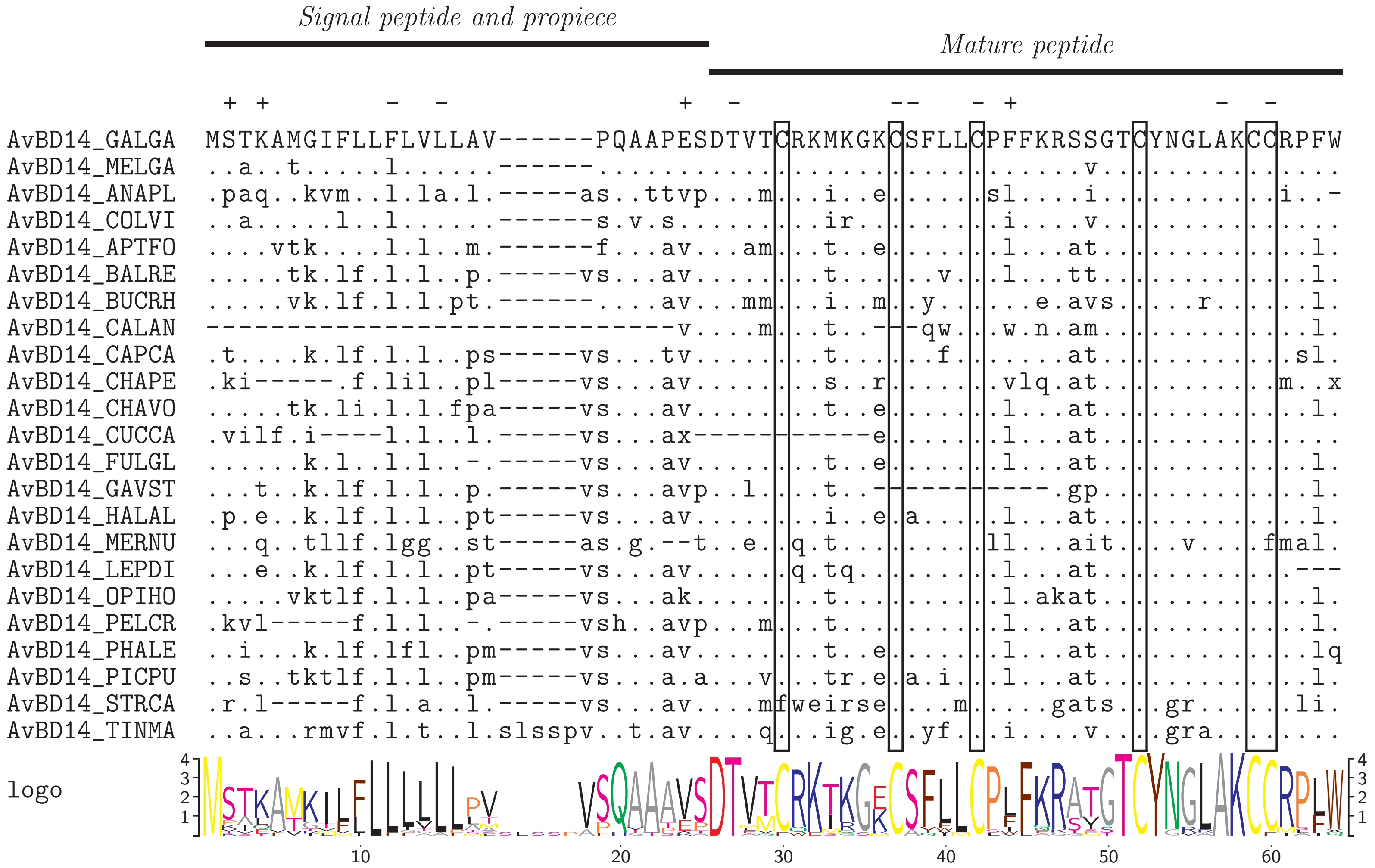

Signal peptide and propiece
Mature peptide
AvBD14_GALGA MSTKAMGIFLLFLVLLAV------PQAAPESDTVTCRKMKGKCSFLLCPFFKRSSGTCYNGLAKCCRPFW
AvBD14_MELGA
AvBD14_ANAPL
AvBD14_COLVI
AvBD14_APTFO
AvBD14_BALRE
AvBD14_BUCRH
AvBD14_CALAN
AvBD14_CAPCA
AvBD14_CHAPE
AvBD14_CHAVO
AvBD14_CUCCA
AvBD14_FULGL
AvBD14_GAVST
AvBD14_HALAL
AvBD14_MERNU
AvBD14_LEPDI
AvBD14_OPIHO
AvBD14_PELCR
AvBD14_PHALE
AvBD14_PICPU
AvBD14_STRCA
AvBD14_TINMA
logo
10
20
30
40
50
60

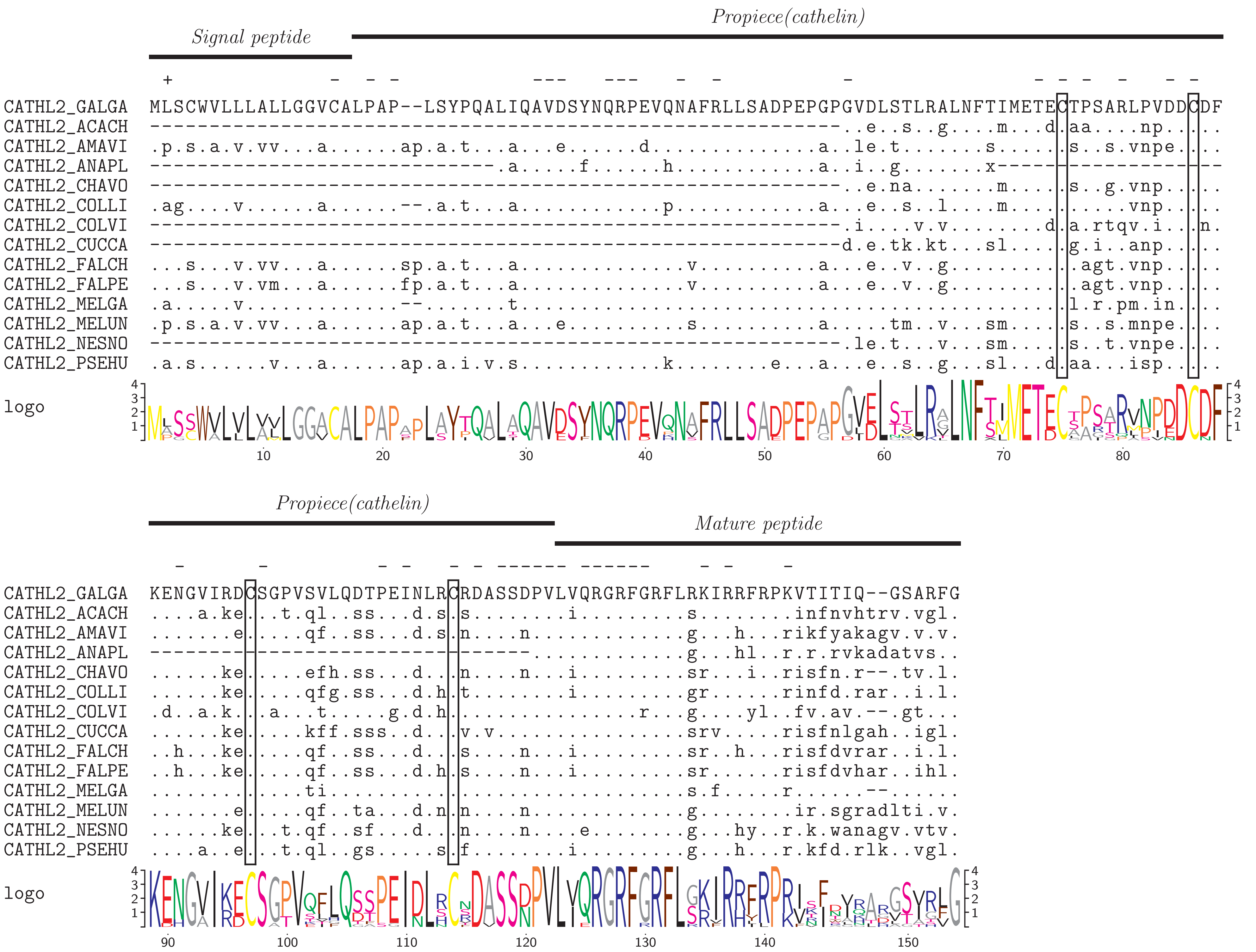

Signal peptide
Propiece(cathelin)
CATHL2_GALGA MLSCWVLLLALLGGVCALPAP--LSYPQALIQAVDSYNQRPEVQNAFRLLSADPEPGPGVDLSTLRALNFTIMETECTPSARLPVDDCDF
CATHL2_ACACH
CATHL2_AMAVI
CATHL2_ANAPL
CATHL2_CHAVO
CATHL2_COLLI
CATHL2_COLVI
CATHL2_CUCCA
CATHL2_FALCH
CATHL2_FALPE
CATHL2_MELGA
CATHL2_MELUN
CATHL2_NESNO
CATHL2_PSEHU
logo
Propiece(cathelin)
Mature peptide
CATHL2_GALGA KENGVIRDCSGPVSVLQDTPEINLRCRDASSDPVLVQRGRFGRFLRKIRRFRPKVTITIQ--GSARFG
CATHL2_ACACH
CATHL2_AMAVI
CATHL2_ANAPL
CATHL2_CHAVO
CATHL2_COLLI
CATHL2_COLVI
CATHL2_CUCCA
CATHL2_FALCH
CATHL2_FALPE
CATHL2_MELGA
CATHL2_MELUN
CATHL2_NESNO
CATHL2_PSEHU
logo

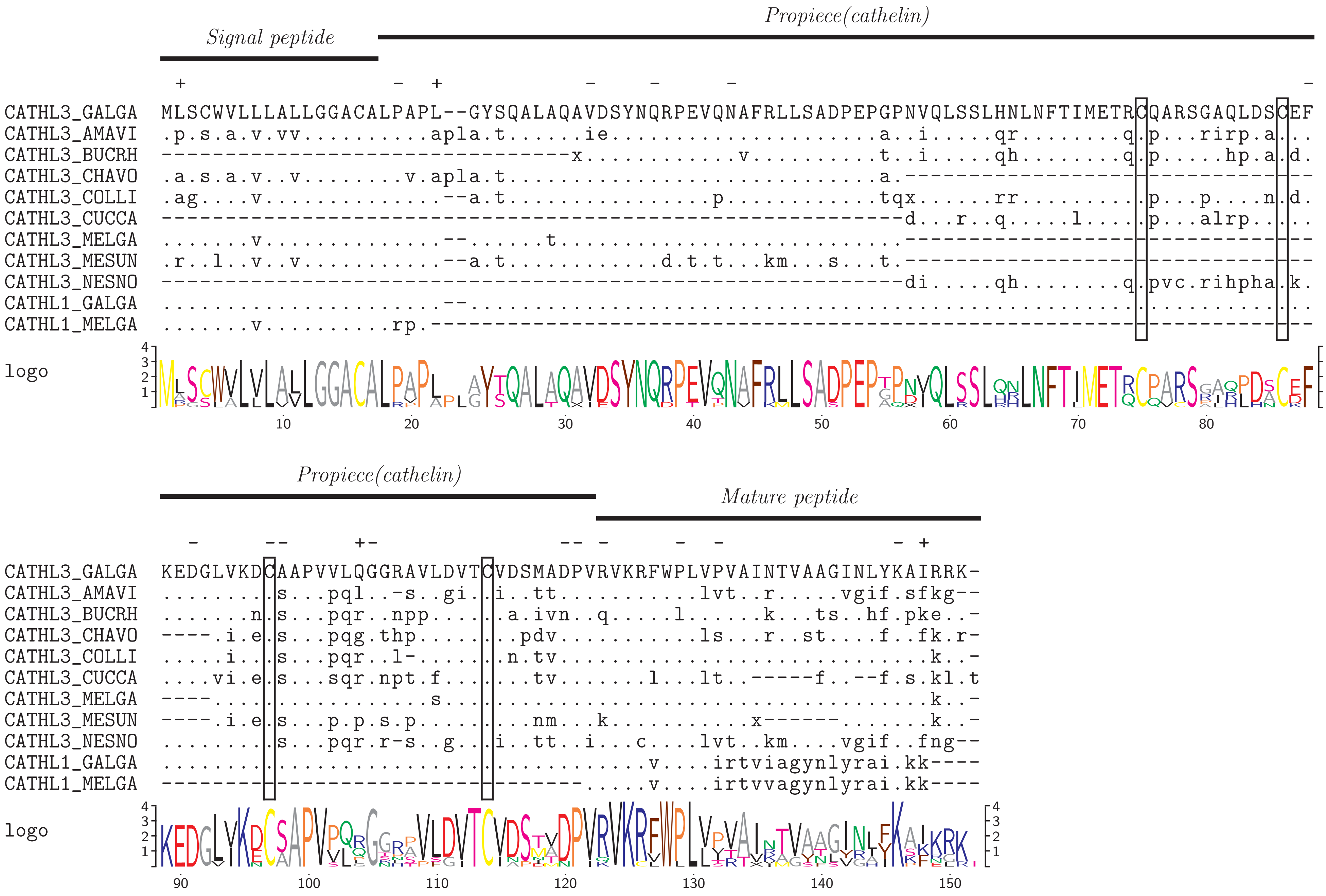

Signal peptide
Propiece(cathelin)
CATHL3_GALGA MLSCWVLLLALLGGACALPAPL--GYSQALAQAVDSYNQRPEVQNAFRLLSADPEPGPNVQLSSLHNLNFTIMETRCQARSGAQLDSCEF
CATHL3_AMAVI
CATHL3_BUCRH
CATHL3_CHAVO
CATHL3_COLLI
CATHL3_CUCCA
CATHL3_MELGA
CATHL3_MESUN
CATHL3_NESNO
CATHL1_GALGA
CATHL1_MELGA
logo
10 20 30 40 50 60 70 80
Propiece(cathelin)
Mature peptide
CATHL3_GALGA KEDGLVKDCAAPVVLQGGRAVLDVTCVDSMADPVRVKRFWPLVPVAINTVAAGINLYKAIRRK-
CATHL3_AMAVI
CATHL3_BUCRH
CATHL3_CHAVO
CATHL3_COLLI
CATHL3_CUCCA
CATHL3_MELGA
CATHL3_MESUN
CATHL3_NESNO
CATHL1_GALGA
CATHL1_MELGA
logo
90 100 110 120 130 140 150

```
                                                                          Octamer repeats
                     Signal peptide
                                         -
CATHB1_GALGA  MGRMWASE----VLLLLLL--GSSRAVTPGLDVSTAPGLDGSIPPGLDGSIPPGLDGSVSPGLDGSVSPGLDGSVSPGLDGSASPGLDGSTP
CATHB1_MELGA  .s...p..----.......ll...............av..............v.s......ps......p......ip......ip......it
CATHB1_CHAVO  .rpwq.vs----p......gl.lak.t.----------------------------------------------------------------
CATHB1_COLLI  ..lcr.vs----p.....vla.---.t.---------------------------------------------------------------.
CATHB1_CUCCA  .rpcp.vl----l....v---.l...t.----------------------------------------------------------------
CATHB1_FALCH  .rpcqvv-----p.....---.la..tm---------------------------------------------------------..pe...-
CATHB1_FALPE  .rpcr.v-----p.....---.la..tm---------------------------------------------------------..pe...-
CATHB1_FICAL  ..prr.------pq.....al.la-----------------------------------------------------------------gas
CATHB1_GEOFO  --------------------------------------------------------------------------------------------
CATHB1_MELUN  .rpcqvl-----p.....---.la..t.----------------------------------------------------------------
CATHB1_NESNO  --------------------------------------------------------------------------------------------
CATHB1_CAPCA  .wpcpvvppppllp.....---.la..t.---------------------------------------------------------------
CATHB1_NIPNI  -----rlr---ll.gragatmpepags.----------------------------------------------------------------
CATHB1_PSEHU  ..pcr.ls----p.....---.lag.s.----------------------------------------------------------------
CATHB1_AMAVI  .rlcqvl----p......---.la..t.----------------------------------------------------------------
CATHB1_APTFO  -------------p.....---.lag.t.---------------------------------------------------------------
logo
```

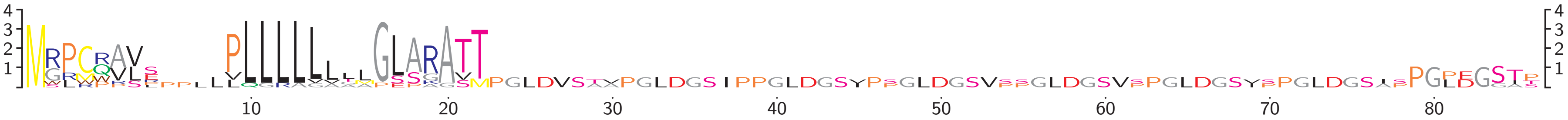


```
                                              Cathelin domain
   + -  -      -                  -a- - --    +  -   -   -     --      b    -   - -----
CATHB1_GALGA  AGRDGTITPKLEGTITPKQDGSISPSWPWRWPI--TYLDAILAAVRLLNQRISGPCILRLREAQPRPGWVGTLQRRREVSFLVEDGPC-PPG
CATHB1_MELGA  pkl..s.....d.p.......y..........s---..............................t....a...q.......k.......v...a..-...
CATHB1_CHAVO  ---------.asd.s-..gla...p.---gllav--s.g..vs.t.e...a.avs.yv.......dq.....s.d.rn.q.l..t..ett.ra...
CATHB1_COLLI  gtp..s-.lgtd.s-..gva..v...pwgc.av--..e.vva...e...akaiv.nv....qqlr..x--aed..q.q.l..t..et-.rs..
CATHB1_CUCCA  ptp..s-..a.v.s-.reen.....ag.pl.av--s.e.i.s.vlk...t.avs.yv...vh.q...lrd.r..q.l..s..ets.pa..
CATHB1_FALCH  v.le.s-.rrp..s-.lgl....l..a.gl.av--s.e..vs...e...t.pvs.yv....dt.......aad..hqq.l..t..ets.ra.-
CATHB1_FALPE  v.le.s-.rrp..s-.lgl....l..a.gl.av--s.e..vs...e...t.pvs.yv....dt..w...avd..hqq.l..t..ets.ra.-
CATHB1_FICAL  tpgp.gs.aagd.-----re.t-...s.g--al--s.g.vva...a...a.avs.yv..............psd..g.q.l..tl.ett.rt..
CATHB1_GEOFO  -------------------------------------------.gg.papgaas.yv..............psd..gqq.l..tl.ett.ra..
CATHB1_MELUN  ---------.gpd.s-..gsa...pv.v.gl.tv--s.e..v.....e...t.vvt.yv...q........p.d..hpq.l..t..ets.ra..
CATHB1_NESNO  -----------------------------------------------------------------------xp.d....q.l..t..ets.ra..
CATHB1_CAPCA  ---------.vpd.s-..gltr..p..p.g.glv--s.k.v.s...d...s.avs.yv.....v......ptd.rq.q.l..t..ets.pa..
CATHB1_NIPNI  ---------.gsd.s-..gla..vp..p.--.vv--s.e..vs...e...m.avs.yv...w.v.hq...pwd..n...l..a..ets.ra..
CATHB1_PSEHU  ---------.eph.s-.ag.n.ttlgwegtapsssls.gavva...e...a.avs.yv..............psd..s.q.l..tl.ett.ra..
CATHB1_AMAVI  ---------.gpd.s-.lgsae..pv.l.gl.tv--s.e..vs...e...t.vvtlyv..............p.d...pq.l..t..ets.ra..
CATHB1_APTFO  ---------.gpd.s-.lglte.vp..ptgl.av--s.e..vs...er..a.avs.yv..............p.d..hpq.l..i..etr.ra..
logo
```

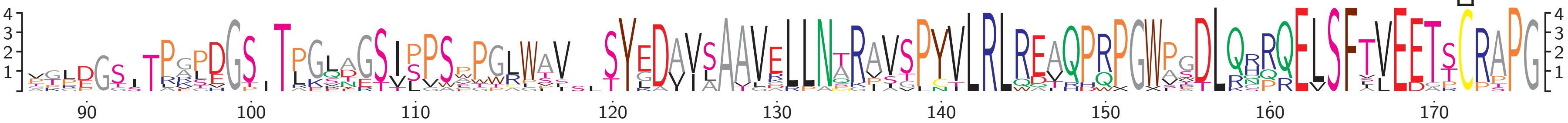


```
                 Cathelin domain                          Mature peptide
         +   -    -      -- - - -  +         b   a        -- -  -    -
CATHB1_GALGA  VDCRSCE---PGALQHCVGTVSIE-QQPTAELRCRPLRPQPIRNWWIRIWEWLNGIRKRLRQRSPFYV--RGHLNVTSTPQP
CATHB1_MELGA  ......---l....r.......m.-.........g...v.......t..r..wd...............--..r..i......
CATHB1_CHAVO  tataa.ksrwl..vsw.r.y.fl.q...vv..s.era.stlg.iqts.lkdffak.ke.f.gffqcgkiwirdklnltk...
CATHB1_COLLI  tvtta.kscwf.t.rw.q.w.fl.q...vv..s.qwvp.mvg.irpsplrdl.ar..dq..nii.cgniwirdklnlqp.k.
CATHB1_CUCCA  .atdt.ktfwf..vsq.r.h.fl.e....v..s.ekvpitfg.frpstfkdffer.ke...sffrcgkiwirdklnlq.sk.
CATHB1_FALCH  lagtt.qgprar--------------------------------fg.lqts.lagffar.ke.f.gffqcsriwirdklnlkq..a
CATHB1_FALPE  lavtt.qgrw.r.vaw.r.s.fl.lr......s.ervprtfg.lqts.lagffar.ke.f.gffqcsriwirdklnlkq..a
CATHB1_FICAL  tangt.rsrwl.vvtw.q.s.fl.g....v..s.ekapatlg.i.ksk.kdffgkvkl.fqsffqcgriwirdrlnika.k.
CATHB1_GEOFO  matsn.ksrwl..vtw.q.s.fl.g....v..----paafgqv.ksk.kdffgkvkq.fhgffqcgriwirdrlnlka.k.
CATHB1_MELUN  mgtaa.ksqwf..vsw.h.y..l.q...mv..s.ekmpvalgpirksg.knlfgr.ke.fkgffqcsniwirdklnlkk.rs
CATHB1_NESNO  mataa.ksrwf..isw.q.yafl.q...mv..s.ekmpitlgtirks.vkdl.gr.ke.fkgffqrskiwirdklnlkk.ks
CATHB1_CAPCA  tatta.ksrwl..vsw.r.g.fl.q...mv..s.ekvpikfg.irrskvrgl.tk.kdg..sffqcskiwirdklnln----
CATHB1_NIPNI  tataa.rspwlrgvnw.r.sifl.q...mv..s.etvptalg.tgkp.ladifar.kah..affqrskiwirdklnlkk.ka
CATHB1_PSEHU  matsd.ksrwl...tw.q.s.fl.g....v..s.kkapavfgqs.ksk.kdffgkvke.fqgffqcgriwirdklnlka.k.
CATHB1_AMAVI  matta.kshwf..vsw.r.ytfl.q...mv..s.ekmpitlgsirksgvrnlfgr.ke.fkgffqcskiwirdklnlkk.ks
CATHB1_APTFO  mataa.rsrwl.vviw.q.s.fl.q....v..s.ek----lg.tqts.lmrlfarl.ehfggffqcgkiwirdkx-------
logo
```

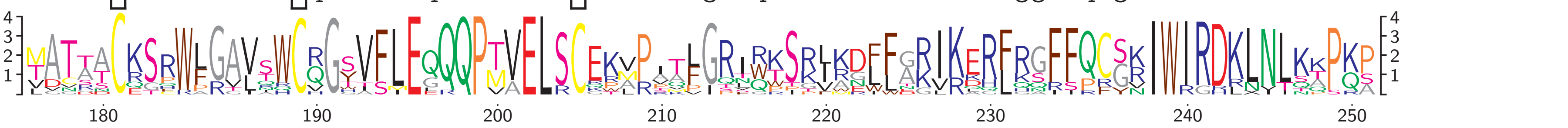

Supplement: Additional file 3: — Sequence alignments of avian β-denfensins and cathelicidins. The ruler is based on residue positions of chicken sequences. Dots represent identical residues in comparison with the chicken sequence in the top; dashes are gaps or missing data. Functionally significant conserved residues are boxed. Plus and minus signs on top of the alignment indicate sites under positive and negative selection, respectively. Letters ‘a’ and ‘b’ above the CATHB1 alignment indicate two pairs of amino acid residues with evidence of intra-molecular co-evolution. A sequence logo was generated at the bottom of the alignment to show the frequency of certain amino acid substitution at a position, indicated by the height of the letter [66]. The labelled alignments and sequence logo were produced with TEXshade [67]. (PDF 1538 kb) [file 12862_2015_465_MOESM3_ESM.pdf]
