# Supplementary material for: Evolution of the avian β-defensin and cathelicidin genes
Source: BMC Evol Biol. 2015 Sep 15;15:188. doi: 10.1186/s12862-015-0465-3 (PMC4571063; doi:10.1186/s12862-015-0465-3)
Supplement: Additional file 7: — Reconstructed avian and primate ancestral cathelicidin sequences and inferred charge changes between nodes. (PDF 299 kb) [file 12862_2015_465_MOESM7_ESM.pdf]

Reconstructed ancestral avian cathelicidin sequences

```
1. CATHB1_GALGA     MGRMWASEVLLLLLL--GSSRAVTPGLDVSTAPGLDGSIPPGLDGSIPPGLDGSVSPGLDGSVSPGLDGSVSPGLDGSASPGLDGSTPAGRDGTITPKLEGTITPKQDGSISPSWPWRWPITYLDAILAAVRLLNQRISGPCILRLREAQPRPGWVGTLQRRREVSFLVEDGPC-PPGVDCRSCE---PGALQHCVGTVSIE-QQPTAELRCRPLRPQPIRNWWIRIWEWLNGIRKRLR---QRSPFYVRGHLNVTSTPQP
2. CATHB1_MELGA     MSRMWPSEVLLLLLLLLGSSRAVTPGLDVSAVPGLDGSIPPGLDGSVPSGLDGSVPSGLDGSVPPGLDGSIPPGLDGSIPPGLDGSITPKLDGSITPKLDGPITPKQYGSISPSWPWRWS-TYLDAILAAVRLLNQRISGPCTLRLRAAQPQPGWVGTLKRRREVSFVVEDAPC-PPGVDCRSCE---LGALQRCVGTVSME-QQPTAELRCGPLRVQPIRNWWTRIREWWDGIRKRLR---QRSPFYVRGRLNITSTPQP
3. CATHB1_CHAVO     MRPWQAVSPLLLLLLGLGLAKATT------------------------------------------------PASDGS-----------------TPGLAG--------SIPP---GLLAVSYGDAVSATVELLNARAVSPYVLRLREAQDQPGWSGDLRNRQELSFTVEETTCRAPGTATAACKSRWLGAVSWCRGYVFLEQQQPVVELSCERARSTLGRIQTSRLKDFFAKIKERFRGFFQCGKIWIRDKLNLT-KPQP
4. CATHB1_COLLI     MGLCRAVSPLLLLL---VLAGATT-----------------------------------------------PGTPDGS-----------------TLGTDGS-TPGVAGSVSPSPWGCWAVTYEDVVAAAVELLNAKAIVPNVLRLQQLRPR---AEDLQQRQELSFTVEET-CRSPGTVTTACKSCWFGTLRWCQGWVFLEQQQPVVELSCQWVPPMVGRIRPSPLRDLLARIRDQLRNIIPCGNIWIRDKLNLQ-PPKP
5. CATHB1_CUCCA     MRPCPAVLLLLLLV---GLSRATT------------------------------------------------PTPDGS-----------------TPALVGS-TREENGSISPAGPPLWAVSYEDIISAVLKLLNTRAVSPYVLRLREVHPQPGWLRDLRRRQELSFSVEETSCPAPGVATDTCKTFWFGAVSQCRGHVFLEEQQPTVELSCEKVPITFGRFRPSTFKDFFERIKERLRSFFRCGKIWIRDKLNLQ-TSKP
6. CATHB1_FALPE     MRPCRAV-PLLLLL---GLARATM------------------------------------------------PGPEGST-VGLEGS-TRRPEGS-TLGLDG--------SILPSAPGLWAVSYEDAVSAAVELLNTRPVSPYVLRLRDTQPWPGWAVDLQHQQELSFTVEETSCRAP-LAVTTCQGRWPRAVAWCRGSVFLELRQPTAELSCERVPRTFGRLQTSRLAGFFARIKERFRGFFQCSRIWIRDKLNLK-QPQA
7. CATHB1_MELUN     MRPCQVL-PLLLLL---GLARATT------------------------------------------------PGPDGS-----------------TPGSAG--------SIPVSVPGLWTVSYEDAVLAAVELLNTRVVTPYVLRLQEAQPRPGWPGDLQHPQELSFTVEETSCRAPGMGTAACKSQWFGAVSWCHGYVSLEQQQPMVELSCEKMPVALGPIRKSGIKNLFGRIKERFKGFFQCSNIWIRDKLNLK-KPRS
8. CATHB1_PSEHU     MGPCRALSPLLLLL---GLAGAST------------------------------------------------PEPHGS-----------------TAGQNGTTLG-WEGT-APSSSL----SYGAVVAAAVELLNARAVSPYVLRLREAQPRPGWPSDLQSRQELSFTLEETTCRAPGMATSDCKSRWLGALTWCQGSVFLEGQQPTVELSCKKAPAVFGQSWKSKIKDFFGKVKERFQGFFQCGRIWIRDKLNLK-APKP
9. CATHB1_AMAVI     MRLCQVL-PLLLLLL--GLARATT------------------------------------------------PGPDGS-----------------TLGSAE--------SIPVSLPGLWTVSYEDAVSAAVELLNTRVVTLYVLRLREAQPRPGWPGDLQRPQELSFTVEETSCRAPGMATTACKSHWFGAVSWCRGYTFLEQQQPMVELSCEKMPITLGSIRKSGVRNLFGRIKERFKGFFQCSKIWIRDKLNLK-KPKS
10. CATHB1_APTFO    --------PLLLLL---GLAGATT------------------------------------------------PGPDGS-----------------TLGLTE--------SVPPSPTGLWAVSYEDAVSAAVERLNARAVSPYVLRLREAQPRPGWPGDLQHPQELSFIVEETRCRAPGMATAACRSRWLGVVIWCQGSVFLEQQQPTVELSCEK----LGRTQTSRLMRLFARLREHFGGFFQCGKIWIRDKX--------
11. CATHB1_FICAL    MGPRRA--PQLLLLLALGLAGAST------------------------------------------------PGPGGS-----------------TAAGDG---REGT---SPSSPGAL--SYGDVVAAAVALLNARAVSPYVLRLREAQPRPGWPSDLQGRQELSFTLEETTCRTPGTANGTCRSRWLGVVTWCQGSVFLEGQQPTVELSCEKAPATLGRIWKSKIKDFFGKVKLRFQSFFQCGRIWIRDRLNIK-APKP
12. CATHB1_NIPNI    ------------------------------------------------------------------------PGSDGS-----------------TPGLAG--------SVPPSPP--WVVSYEDAVSAAVELLNMRAVSPYVLRLWEVQHQPGWPWDLQNRRELSFAVEETSCRAPGTATAACRSPWLRGVNWCRGSIFLEQQQPMVELSCETVPTALGRTGKPRLADIFARIKAHLRAFFQRSKIWIRDKLNLK-KPKA
Ancestral
 25. (7 . 9)       MRPCQVL-PLLLLLL--GLARATT------------------------------------------------PGPDGS-----------------TPGSAE--------SIPVSVPGLWTVSYEDAVSAAVELLNTRVVTPYVLRLREAQPRPGWPGDLQHPQELSFTVEETSCRAPGMATAACKSHWFGAVSWCRGYVFLEQQQPMVELSCEKMPITLGPIRKSGIKNLFGRIKERFKGFFQCSKIWIRDKLNLK-KPKS
 26. (25 . 10)     MRPCQAV-PLLLLLL--GLARATT------------------------------------------------PGPDGS-----------------TPGLAE--------SIPPSPPGLWAVSYEDAVSAAVELLNARAVSPYVLRLREAQPRPGWPGDLQHPQELSFTVEETSCRAPGMATAACKSRWLGAVSWCRGSVFLEQQQPMVELSCEKVPTTLGRIQTSRLKNLFARIKERFRGFFQCGKIWIRDKLNLK-KPKP
 27. (26 . 12)     MRPCQAV-PLLLLLL--GLARATT------------------------------------------------PGPDGS-----------------TPGLAG--------SIPPSPPGLWAVSYEDAVSAAVELLNARAVSPYVLRLREAQPRPGWPGDLQNRQELSFTVEETSCRAPGMATAACKSRWLGAVSWCRGSVFLEQQQPMVELSCEKVPTTLGRIQTSRLKDFFARIKERFRGFFQCGKIWIRDKLNLK-KPKP
 28. (27 . 3)      MRPCQAVSPLLLLLLGLGLARATT------------------------------------------------PGPDGS-----------------TPGLAG--------SIPPSPPGLWAVSYEDAVSAAVELLNARAVSPYVLRLREAQPRPGWPGDLQHRQELSFTVEETSCRAPGMATAACKSRWLGAVSWCRGSVFLEQQQPMVELSCEKVPTTLGRIQTSRLKDFFARIKERFRGFFQCGKIWIRDKLNLK-KPKP
 29. (28 . 6)      MRPCRAVSPLLLLLLGLGLARATT------------------------------------------------PGPDGST-VGLEGS-TRRPEGS-TPGLDG--------SIPPSPPGLWAVSYEDAVSAAVELLNARAVSPYVLRLREAQPRPGWPGDLQHRQELSFTVEETSCRAPGMATATCKSRWLGAVSWCRGSVFLEQQQPTVELSCERVPTTFGRIQTSRLKDFFARIKERFRGFFQCGKIWIRDKLNLK-KPKP
 30. (8 . 11)      MGPCRALSPLLLLLLALGLAGAST------------------------------------------------PGPDGS-----------------TAGQDGTTLRESEGT-SPSSPGAW--SYGDVVAAAVELLNARAVSPYVLRLREAQPRPGWPSDLQSRQELSFTLEETTCRAPGMATSTCKSRWLGAVTWCQGSVFLEGQQPTVELSCEKAPATFGRIWKSKIKDFFGKVKERFQGFFQCGRIWIRDKLNLK-APKP
 31. (29 . 30)     MRPCRAVSPLLLLLLGLGLARATT------------------------------------------------PGPDGST-VGLEGS-TRRPEGS-TPGLDGSTTREENGSISPSPPGLWAVSYEDVVSAAVELLNARAVSPYVLRLREAQPRPGWPGDLQHRQELSFTVEETSCRAPGMATATCKSRWLGAVSWCRGSVFLEQQQPTVELSCERVPTTFGRIRTSRLKDFFARIKERFRGFFQCGKIWIRDKLNLK-TPKP
 32. (31 . 5)      MRPCRAVSPLLLLLLGLGLARATT------------------------------------------------PGPDGST-VGLEGS-TRRPEGS-TPGLDGSTTREENGSISPSPPGLWAVSYEDVVSAAVELLNARAVSPYVLRLREAQPRPGWPGDLQHRQELSFTVEETSCRAPGMATATCKSRWLGAVSWCRGSVFLEQQQPTVELSCERVPTTFGRIRTSRLKDFFARIKERFRGFFQCGKIWIRDKLNLQ-TPKP
 33. (32 . 4)      MRPCRAVSPLLLLLLGLGLARATT-----------------------------------------------PPGPDGST-VGLEGS-TRRPEGS-TPGLDGSTTREENGSISPSPPGLWAVSYEDVVSAAVELLNARAVSPYVLRLREAQPRPGWPGDLQHRQELSFTVEETSCRAPGMATATCKSRWLGAVSWCRGSVFLEQQQPTVELSCERVPTTFGRIRPSRLKDFFARIKERFRGFFQCGKIWIRDKLNLQ-TPKP
 34. (1 . 2)       MSRMWASEVLLLLLLLLGSSRAVTPGLDVSAVPGLDGSIPPGLDGSVPSGLDGSVPSGLDGSVPPGLDGSIPPGLDGSIPPGLDGSITPKRDGSITPKLDGPITPKQNGSISPSWPWRWSITYLDAILAAVRLLNQRISGPCILRLREAQPRPGWVGTLQRRREVSFVVEDAPC-PPGVDCRSCE---LGALQRCVGTVSME-QQPTAELRCGPLRVQPIRNWWTRIREWWDGIRKRLR---QRSPFYVRGRLNITSTPQP
 35. (33 . 34)     MRPCWAVSPLLLLLLALGSARATT-----------------------------------------------PPGPDGST-PGLEGS-TRRPEGS-TPGLDGSITPEQNGSISPSPPGLWAVTYEDAVSAAVELLNQRAVSPYVLRLREAQPRPGWPGDLQHRQELSFTVEETSCRPPGMATATCKSRWLGAVSWCRGSVFLEQQQPTVELSCERVPPTLVRIWWSRLKDWWARIRERLRGFFQCGKIWIRDKLNLQ-TPKP

13. CATHL1_GALGA    MLSCWVLLLALLGGACALPAP--LGYSQALAQAVDSYNQRPEVQNAFRLLSADPEPGPNVQLSSLHNLNFTIMETRCQARSGAQLDSCEFKEDGLVKDCAAPVVLQGGRAVLDVTCVDSMADPVRVKR-VWPLV---IRTVIAGYNLYRAIKKK----
14. CATHL3_GALGA    MLSCWVLLLALLGGACALPAP--LGYSQALAQAVDSYNQRPEVQNAFRLLSADPEPGPNVQLSSLHNLNFTIMETRCQARSGAQLDSCEFKEDGLVKDCAAPVVLQGGRAVLDVTCVDSMADPVRVKR-FWPLVPVAINTVAAGINLYKAIRRK----
15. CATHL3_AMAVI    MPSSWALVLVVLGGACALPAPAPLAYTQALAQAIESYNQRPEVQNAFRLLSADPEPAPNIQLSSLQRLNFTIMETQCPARSRIRPDACEFKEDGLVKDCSAPVPQLGG-SVLGITCIDSTTDPVRVKR-FWPLLVTAIRTVAAGVGIFKSFKG-----
16. CATHL3_COLLI    MAGCWVLVLALLGGACALPAP--LAYTQALAQAVDSYNQRPEVPNAFRLLSADPEPTQNVQLSSLRRLNFTIMETRCPARSPAQLDNCDFKEDGLIKDCSAPVPQRGGL-VLDVTCVNSTVDPVRVKR-FWPLVPVAINTVAAGINLYKAIKRK----
17. CATHL2_GALGA    MLSCWVLLLALLGGVCALPAP--LSYPQALIQAVDSYNQRPEVQNAFRLLSADPEPGPGVDLSTLRALNFTIMETECTPSARLPVDDCDFKENGVIRDCSGPVSVLQDTPEINLRCRDASSDPVLVQRGRFGRFLRKIRRFRPKVTITIQ--GSARFG
18. CATHL2_AMAVI    MPSSWALVLVVLGGACALPAPAPLAYTQALAQAVESYNQRPDVQNAFRLLSADPEPAPGLELTTLRALNFSIMETECSPSSRVNPEDCDFKENGVIRECSGPVQFLQSSPEIDLSCNDASSNPVLVQRGRFGRFLGKIRHFRPRIKFYAKAGVSVRVG
19. CATHL2_COLLI    MAGCWVLVLALLGGACALPAP--LAYTQALAQAVDSYNQRPEVPNAFRLLSADPEPAPGVELSSLRLLNFTMMETECTPSARVNPDDCDFKENGVIKECSGPVQFGQSSPEIDLHCTDASSDPVLIQRGRFGRFLGRIRRFRPRINFDIRARGSIRLG
20. CATHL2_FALCH    MLSSWVLVLVVLGGACALPAPSPLAYTQALAQAVDSYNQRPEVQNVFRLLSADPEPAPGVELSVLRGLNFTIMETECTAGTRVNPDDCDFKEHGVIKECSGPVQFLQSSPEIDLRCSDASSNPVLIQRGRFGRFLSRIRHFRPRISFDVRARGSIRLG
21. CATHL2_FALPE    MLSSWVLVLVMLGGACALPAPFPLAYTQALAQAVDSYNQRPEVQNVFRLLSADPEPAPGVELSVLRGLNFTIMETECTAGTRVNPDDCDFKEHGVIKECSGPVQFLQSSPEIDLHCSDASSNPVLIQRGRFGRFLSRIRRFRPRISFDVHARGSIHLG
22. CATHL2_MELGA    MASCWVLVLALLGGVCALPAP--LSYPQALTQAVDSYNQRPEVQNAFRLLSADPEPGPGVDLSTLRALNFTIMETECLPRAPMPINDCDFKENGVIRDCSGPVTILQDTPEINLRCRDASSDPVLVQRGRFGRFLSKFRRFRPRVTITIQ--GSARFG
23. CATHL2_MELUN    MPSSWALVLVVLGGACALPAPAPLAYTQALAQAVESYNQRPEVQNSFRLLSADPEPAPGVDLTMLRVLNFSMMETECSPSSRMNPEDCDFKENGVIRECSGPVQFLQTAPEIDLNCNDASSNPVLVQRGRFGRFLGKIRRFRPKIRISGRADLTIRVG
24. CATHL2_PSEHU    MASSWVLLLAVLGGACALPAPAPLAYIQVLSQAVDSYNQRPEVKNAFRLLSAEPEPAPGVELSSLRGLNFSLMETDCAASARISPDDCDFKENGAIRECSGTVQLLQGSPEINLSCFDASSDPVLIQRGRFGRFLGKIRHFRPRVKFDIRLKGSVGLG
Ancestral
 36. (13 . 14)     MLSCWVLLLALLGGACALPAP--LGYSQALAQAVDSYNQRPEVQNAFRLLSADPEPGPNVQLSSLHNLNFTIMETRCQARSGAQLDSCEFKEDGLVKDCAAPVVLQGGRAVLDVTCVDSMADPVRVKR-FWPLVPVAINTVAAGINLYKAIKRK----
 37. (36 . 16)     MASCWVLVLALLGGACALPAP--LAYTQALAQAVDSYNQRPEVQNAFRLLSADPEPAPNVQLSSLRRLNFTIMETRCPARSPAQLDNCEFKEDGLIKDCSAPVPQQGGRAVLDVTCVDSTVDPVRVKR-FWPLVPVAINTVAAGINLYKAIKRK----
 38. (37 . 15)     MPSCWVLVLAVLGGACALPAPAPLAYTQALAQAVDSYNQRPEVQNAFRLLSADPEPAPNIQLSSLRRLNFTIMETQCPARSRIRPDNCEFKEDGLVKDCSAPVPQLGGRSVLDITCIDSTTDPVRVKR-FWPLLVTAIRTVAAGVNIYKSIKGK----
 39. (17 . 22)     MASCWVLVLALLGGVCALPAP--LSYPQALTQAVDSYNQRPEVQNAFRLLSADPEPGPGVDLSTLRALNFTIMETECTPSARMPVDDCDFKENGVIRDCSGPVSILQDTPEINLRCRDASSDPVLVQRGRFGRFLSKIRRFRPRVTITIQ--GSARFG
 41. (20 . 21)     MLSSWVLVLVVLGGACALPAPSPLAYTQALAQAVDSYNQRPEVQNVFRLLSADPEPAPGVELSVLRGLNFTIMETECTAGTRVNPDDCDFKEHGVIKECSGPVQFLQSSPEIDLHCSDASSNPVLIQRGRFGRFLSRIRRFRPRISFDVRARGSIRLG
 40. (18 . 23)     MPSSWALVLVVLGGACALPAPAPLAYTQALAQAVESYNQRPEVQNAFRLLSADPEPAPGVELTTLRALNFSIMETECSPSSRVNPEDCDFKENGVIRECSGPVQFLQSSPEIDLSCNDASSNPVLVQRGRFGRFLGKIRRFRPRIKFNARAGVSIRVG
 42. (19 . 41)     MASCWVLVLAVLGGACALPAPAPLAYTQALAQAVDSYNQRPEVQNAFRLLSADPEPAPGVELSTLRALNFTIMETECTPSARVNPDDCDFKENGVIKECSGPVQFLQSSPEIDLHCSDASSNPVLIQRGRFGRFLGRIRRFRPRINFDIRARGSIRLG
 43. (40 . 42)     MASCWVLVLAVLGGACALPAPAPLAYTQALAQAVDSYNQRPEVQNAFRLLSADPEPAPGVELSTLRALNFTIMETECTPSARVNPDDCDFKENGVIRECSGPVQFLQSSPEIDLSCSDASSNPVLIQRGRFGRFLGKIRRFRPRINFDIRARGSIRLG
 44. (24 . 43)     MASCWVLVLAVLGGACALPAPAPLAYTQALAQAVDSYNQRPEVQNAFRLLSADPEPAPGVELSTLRALNFTIMETECTPSARVNPDDCDFKENGVIRECSGPVQLLQGSPEIDLSCSDASSDPVLIQRGRFGRFLGKIRRFRPRVKFDIRLKGSIRLG
 45. (39 . 44)     MASCWVLVLAVLGGACALPAPAPLAYTQALAQAVDSYNQRPEVQNAFRLLSADPEPAPGVELSTLRALNFTIMETECTPSARINPDDCDFKENGVIRECSGPVQLLQGSPEIDLSCSDASSDPVLVQRGRFGRFLGKIRRFRPRVNIDIRIKGSIRLG
 46. (38 . 45)     MASCWVLVLAVLGGACALPAPAPLAYTQALAQAVDSYNQRPEVQNAFRLLSADPEPAPGVELSTLRALNFTIMETECTPSARINPDDCDFKENGVIRECSGPVQLLQGSPEIDLSCSDASSDPVLVQRGRFGRFLGKIRRFRPRVNIDIRIKGSIRLG
```

Reconstructed ancestral primate cathelicidin sequences

```
1. CAMP_HOMSA      MGTMKTQRDGHSLGRWSLVLLLLGLVMPLAIIAQVLSYKEAVLRAIDGINQRSSDANLYRLLDLDPRPTMDGDPDTPKPVSFTVKETVCPRTTQQSPEDCDFKKDGLVKRCMGTVTLNQARGSFDISCDKDNKRFALLGDFFRKSKEKIGKEFKRIVQRIKDFLRNLVPRTES
2. CAMP_SAGOE      ---MKTQRDGPSLGRWSLLLLLLGLTMPLAVIGRVLSYQEAVLRAVDGLNQRSSDANLYRLLNLDPRPTMDGDPDTPKPVSFTVKETVCPRTIQRSPEECDFKEDGLVKWCVGTVTLNQAKDSFDISCDKDKRKVARLGGILRKAGEKIGGGLKKIGQKIKDFFGKLAPRTES
3. CAMP_POGPY      ---MKTQMDGHSLGRWSLVLLLLGLVMPLAIVAQVLSYKEAVLHAIDGINQRSSDANLYRILDLDPSLTMDGDPDTPKPVSFTVKETVCPRRTQQSPEDCDFKKDGLVKRCVGTVTLNQARGSFDISCDKDNRRFALLGDFFRKAREKIGEEFKRIVQRIKDFLRNLVPRTES
4. CAMP_PAPPA      ---MKTQRDSPSLGRWSLVLLLLGLVMPLAIVAQVLSYQEAVLRAIDGINQRSSDANLYRLLDLDPRPTMDGDPDTPKPVSFTVKETVCPRTTQKSPEDCDFKEDGLVKRCVGTVILNQARDSFDISCDKDNRRFARLGNFFRKVKEKIGGGLKKVGQKIKDFLGNLVPRTAS
5. CAMP_PREOB      ---MKTQRHGPSLGRWSLVLLLLGLVMPLAIVAQVLSYQEAVLRAIDGINQRSSDANLYRLLDLDPRPTMDGDPDTPKPVSFTVKETVCPRTTQKSPQDCDFKEDGLVKRCVGTVTLNQARDSFDISCDKDNRRFARLGNFFRKAKKKIGRGLKKIGQKIKDFLGNLVPRTES
6. CAMP_PRECR      ---MKTQRHGPSLGRWSLVLLLLGLVMPLAIVAQVLSYQEAVLRALDGINQRSSDANLYRLLDLDPRPTMDGDPDTPKPVSFTVKETVCPRTTQKSPQDCDFKEDGLVKRCVGTVILNQARDSFDISCDKDNRRFARLGNFFRKAKEKIGRGLKKIGQKIKDFWGNLVPRTES
7. CAMP_MACTO      ---MKTQRDSPSLGRWSLVLLLLGLVMPLAIVAQVLSYQEAVLRAIDGINQRSSDANLYRLLDLDPRPTMDGDPDTPKPVSFTVKETVCPRTTQKSPEDCDFKEDGLVKRCVGTVILNQARDSFDISCDKDNRRSARLGNFFRKVKEKIGGGLKKVGQKIKDFLGNLVPRTAS
8. CAMP_HYLMO      ---MKTQRDGHSLGGWSLMLLLLGLLMPLAIVAQVLSYKEAVLRAIDGINQRSSDANLYRLLDLDPRPTMDGDPDTPKPVSFTVKETVCPRTTQQSPEDCDFKKDGLVKRCVGTVTLNQARDSFDISCDKDNRRFASLGNFFRKARKKIGEEFKRIVQRIKDFLQHLIPRTEA
9. CAMP_OMALE      ---MDTQRDSPSLGRWSLVLLLLGLVMPLAIVAQVLSYQEAVLRAIDGINQRSSDANLYRLLDLDPRPTMDGDPDTPKPVSFTVKETVCPRTTQQSPEDCDFKKDGLVKRCMGTVTLNQARDSFDISCDKDNRRFALLGNFFRKAREKIGKEFKRIVQRIKDFLQHLVPRTEA
10. CAMP_OMACO     ---MKTQRDSPSLGRWSLVLLLLGLVMPLAIVAQVLSYQEAVLRAIDGINQRSSDANLYRLLDLDPRPTMDGDPDTPKPVSFTVKETVCPRTTQQSPEDCDFKKDGLVKRCMGTVTLNQARDSFDISCDKDNRRFALPGNFFRKAREKIGKEFKRIVQRIKDFLQHLVPRTEA
11. CAMP_OMAGA     ---MKTQRDSPSLGRWSLVLLLLGLVMPLAIVAQVLSYQEAVLRAIDGINQRSSDANLYRLLDLDPRPTMDGDPDTPKPVSFTVKETVCPRTTQQSPEDCDFKKDGLVKRCVGTVILNQARDSFDISCDKDNRRFALPGNFFRKAREKIGKEFKRIVQRIKDFLQHLVPRTEA
12. CAMP_CEBCA     ---MKTQRDGPSLGRWSLVLLLLGLTMPLAITAQVLSYQEAVLRAVDGLNQRSLDANLYRLLNLDPRPTLDGDPDTPKPVSFTVKETVCPRTIQRSPEECDFKKDGLVKRCVGTVILNQARDSFDISCDKDERKVARLGGFLQKAREKIARGFKKIGQKINDFLGKLAPRTEA
13. CAMP_CERAE     ---MKTQRDGPSLGRWSLVLLLLGLVMPLAIVAQVLSYQEAVLRAIEGINQRSSDANLYRLLDLDPRPTMDGDPDTPKPVSFTVKETVCPRTTQKSPEDCDFKEDGLVKRCVGTVTLNQARDSFDISCDKDNRRFARLGKFFRKVKKKIGGGLKKIGQKIKDFLGNLVPRTAS
14. CAMP_ATEFU     ---MNTQWDSPSLGRWSLVLLLLGLVMPLAIVAQVLSYQEAVLRAIDGINQRSSDANLYRLLDLDPRPTMDGDPDTPKPVSFTVKETVCPRTIQRSPEECDFREDGLVKWCVGTVTLNQAKDSFDISCDKDKRKVAQLGDVLQKAGEKIVRGLKNIGQRIKDFFGKLTPRTES
15. CAMP_PANTR     ---MKTQRDGHSLGRWSLVLLLLGLVMPLAIVAQVLSYKEAVLRAIDGINQRSSDANLYRLLDLDPRPTMDGDPDTPKPVSFTVKETVCPRTTQQSPEDCDFKKDGLVKRCMGTVTLNQARGSFDISCDKDNKRFALLGDFFRKSKEKIGKEFKRIVQRIKDFLRNLVPRTES
16. CAMP_MACMU     ---MKTQRNGPSLGRWSLVLLLLGLVMPLAIVAQVLSYQEAVLRAIDGINQRSSDANLYRLLDLDPRPTMDGDPDTPKPVSFTVKETVCPRTTQKSPEDCDFKEDGLVKRCVGTVILNQARDSFDISCDKDNRRSARLGNFFRKVKEKIGGGLKKVGQKIKDFLGNLVPRTAS
17. CAMP_SAIBO     MGTMKTQRDGPSLGQWS-LLLLLGLTMPLAVIARVLSYQEAVLRAVDGLNQRSSDANLYRLLNLDPRPTVDGDPDTPKPVSFTVKETVCPRTIQRSPEECDFKMDGLVKWCVGTVTLNQAEDSFDISCDKDKRTVARLGSILQKAGEKIGGGLKKIGQKIKDFFGKLAPRTEA
18. CAMP_RHIRO     MGTMKTQRHGPSLGRWSLVLLLLGLVMPLATIAQVLSYQEAVLRAIDGINQRSSDANFYRLLDLDPRPTMDGDPDTPKPVSFTVKETVCPRTTQKSPQDCDFKEDGLVKRCVGTVTLNQARDSFDISCDKDNRRFARLGNFFRKAKEKIGGGLKKIGQKIKDFLGNLVPRTES
19. CAMP_PONAB     METMNTQMDGHSLGRWSLVLLLLGLVMPLAIVAQVLSYKEAVLHAIDGINQRSSDANLYRLLDLDPSLTMDGDPDTPKPVSFTVKETVCPRRTQQSPEDCDFKKDGLVKRCVGTVTLNQARGSFDISCDKDNRRFALLGDFFREAREKIGEEFKRIVQRIKDFLRNLVPRTES
20. CAMP_PAPAN     MGTMKTQRDGPSLGRWSLVLLLLGLVMPLAIVAQVLSYQEAVLRAIDGINQRSSDANLYRLLDLDPRPTMDGDPDTPKPVSFTVKETVCPRTTQKSPEDCDFKEDGLVKRCVGTVILNQARDSFDISCDKDNR-FARLGNFFRKVKKKIGGGLKKVGQKIKDFLGNLVPRTAS
21. CAMP_PANPA     MGTMKTQRDGHSLGRWSLVLLLLGLVMPLAIVAQVLSYKEAVLRAIDGINQRSSDANLYRLLDLDPRPTMDGDPDTPKPVSFTVKETVCPRTTQQSPEDCDFKKDGLVKRCMGTVTLNQARGSFDISCDKDNKRFALLGDFFRKSKEKIGKEFKRIVQRIKDFLRNLVPRTES
22. CAMP_CALJA     MGTMKTQRDGPSLGRWSLVLLLLGLTMPLAVTGRILSYQEAVLHAVDGLNQRSLDANLYRLLNLDPRPTMDGDPDTPKPVSFTVKETVCPRTIQRSPEECDFKEDGLVKWCVGTVTLNQAKDSFDISCDKDKRNVARLGDILQKAREKIEGGLKKLVQKIKDFFGKFAPRTES
23. CAMP_TARSY     ---METQRDGSFLGRWSLVLLLLGLLMPLATTAQTLSYQEAVLRAVDGFNEQSSEPNLYRLLHQDLQPTEDEDPDTPKDVSFTVKETVCPRTSQQPLEQCDFKENGLVKQCVGTVTLDQARGSFDLNCDENTRRPARLGRFFRNIGRRIRDKFRNIRRNLGNFLQNLVPRTES
24. CAMP_CHLSA     MGTMKTQRDGPSLGRWSLVLLLLGLVMPLAIVAQVLSYQEAVLRAIEGINQRSSDANLYRLLDLDPRPTMDGDPDTPKPVSFTVKETVCPRTTQKSPEDCDFKEDGLVKRCVGTVTLNQARDSFDISCDKDNRRFARLGKFFRKVKKKIGGGLKKIGQKIKDFLGNLVPRTAS
25. CAMP_MACFA     MGTMKTQRDSPSLGRWSLVLLLLGLVMPLAIVAQVLSYQEAVLRAIDGINQRSSDANLYRLLDLDPRPTMDGDPDTPKPVSFTVKETVCPRTTQKSPEDCDFKEDGLVKRCVGTVILNQARDSFDISCDKDNRRSARLGNFFRKVKEKIGGGLKKVGQKIKDFLGNLVPRTAS
26. CAMP_NOMLE     MGTMKTQRDGHSLGGWSLMLLLLGLLMPLAIVAQVLSYKEAVLRAIDGINQRSSDANLYRLLDLDPRPTMDGDPDTPKPVSFTVKETVCPRTTQQSPEDCDFKKDGLVKRCMGTVTLNQARDSFDISCDKDNRRFALLGNFFRKAREKIGKEFKRIVQRIKDFLQHLVPRTEA
27. CAMP_GORGO     MGTMKTQRDGHSLGWWSLVLLLLGLVMPLAIIAQVLSYKEAVLRAIDGINQRSSDANLYRLLDLDPRPTMDGDPDTPKPVSFTVKETVCPRTTQQSPEDCDFKKDGLVKRCMGTVTLNQARGSFDISCDKDNKRFALLGDFFRKAKEKIGKESKRIVQRIKDFLRNLVPRTES
28. CAMP_OTOGA     ---METQRDGSFLGWWSLVLLLLGLVTPLAT-AQALSYKEAVLRAVDGFNQRSSEANLYRLLDLEQQSKGDDDPDTPKPVNFLVKETVCSRTTQQPPEQCDFKEDGLVKRCVGTVTLDQIRGSFDITCDENTKKAARLGGFLRRGVEEFGRKLENIGRKIKEFFQNLAPRMES
```

Ancestral

```
29. (15 . 21)     MGTMKTQRDGHSLGRWSLVLLLLGLVMPLAIVAQVLSYKEAVLRAIDGINQRSSDANLYRLLDLDPRPTMDGDPDTPKPVSFTVKETVCPRTTQQSPEDCDFKKDGLVKRCMGTVTLNQARGSFDISCDKDNKRFALLGDFFRKSKEKIGKEFKRIVQRIKDFLRNLVPRTES
30. (29 . 1)      MGTMKTQRDGHSLGRWSLVLLLLGLVMPLAIIAQVLSYKEAVLRAIDGINQRSSDANLYRLLDLDPRPTMDGDPDTPKPVSFTVKETVCPRTTQQSPEDCDFKKDGLVKRCMGTVTLNQARGSFDISCDKDNKRFALLGDFFRKSKEKIGKEFKRIVQRIKDFLRNLVPRTES
31. (30 . 27)     MGTMKTQRDGHSLGRWSLVLLLLGLVMPLAIIAQVLSYKEAVLRAIDGINQRSSDANLYRLLDLDPRPTMDGDPDTPKPVSFTVKETVCPRTTQQSPEDCDFKKDGLVKRCMGTVTLNQARGSFDISCDKDNKRFALLGDFFRKAKEKIGKEFKRIVQRIKDFLRNLVPRTES
32. (3 . 19)      METMKTQMDGHSLGRWSLVLLLLGLVMPLAIVAQVLSYKEAVLHAIDGINQRSSDANLYRLLDLDPSLTMDGDPDTPKPVSFTVKETVCPRRTQQSPEDCDFKKDGLVKRCVGTVTLNQARGSFDISCDKDNRRFALLGDFFRKAREKIGEEFKRIVQRIKDFLRNLVPRTES
33. (31 . 32)     MGTMKTQRDGHSLGRWSLVLLLLGLVMPLAIVAQVLSYKEAVLRAIDGINQRSSDANLYRLLDLDPRPTMDGDPDTPKPVSFTVKETVCPRTTQQSPEDCDFKKDGLVKRCMGTVTLNQARGSFDISCDKDNRRFALLGDFFRKAREKIGKEFKRIVQRIKDFLRNLVPRTES
34. (8 . 26)      MGTMKTQRDGHSLGGWSLMLLLLGLLMPLAIVAQVLSYKEAVLRAIDGINQRSSDANLYRLLDLDPRPTMDGDPDTPKPVSFTVKETVCPRTTQQSPEDCDFKKDGLVKRCMGTVTLNQARDSFDISCDKDNRRFALLGNFFRKAREKIGKEFKRIVQRIKDFLQHLVPRTEA
35. (33 . 34)     MGTMKTQRDGHSLGRWSLVLLLLGLVMPLAIVAQVLSYKEAVLRAIDGINQRSSDANLYRLLDLDPRPTMDGDPDTPKPVSFTVKETVCPRTTQQSPEDCDFKKDGLVKRCMGTVTLNQARDSFDISCDKDNRRFALLGNFFRKAREKIGKEFKRIVQRIKDFLQHLVPRTEA
36. (10 . 11)     ---MKTQRDSPSLGRWSLVLLLLGLVMPLAIVAQVLSYQEAVLRAIDGINQRSSDANLYRLLDLDPRPTMDGDPDTPKPVSFTVKETVCPRTTQQSPEDCDFKKDGLVKRCMGTVTLNQARDSFDISCDKDNRRFALPGNFFRKAREKIGKEFKRIVQRIKDFLQHLVPRTEA
37. (9 . 36)      ---MKTQRDSPSLGRWSLVLLLLGLVMPLAIVAQVLSYQEAVLRAIDGINQRSSDANLYRLLDLDPRPTMDGDPDTPKPVSFTVKETVCPRTTQQSPEDCDFKKDGLVKRCMGTVTLNQARDSFDISCDKDNRRFALLGNFFRKAREKIGKEFKRIVQRIKDFLQHLVPRTEA
38. (35 . 37)     MGTMKTQRDGPSLGRWSLVLLLLGLVMPLAIVAQVLSYQEAVLRAIDGINQRSSDANLYRLLDLDPRPTMDGDPDTPKPVSFTVKETVCPRTTQQSPEDCDFKKDGLVKRCMGTVTLNQARDSFDISCDKDNRRFALLGNFFRKAREKIGKEFKRIVQRIKDFLQHLVPRTEA
39. (5 . 6)       ---MKTQRHGPSLGRWSLVLLLLGLVMPLAIVAQVLSYQEAVLRAIDGINQRSSDANLYRLLDLDPRPTMDGDPDTPKPVSFTVKETVCPRTTQKSPQDCDFKEDGLVKRCVGTVTLNQARDSFDISCDKDNRRFARLGNFFRKAKEKIGRGLKKIGQKIKDFLGNLVPRTES
40. (39 . 18)     MGTMKTQRHGPSLGRWSLVLLLLGLVMPLAIVAQVLSYQEAVLRAIDGINQRSSDANLYRLLDLDPRPTMDGDPDTPKPVSFTVKETVCPRTTQKSPQDCDFKEDGLVKRCVGTVTLNQARDSFDISCDKDNRRFARLGNFFRKAKEKIGRGLKKIGQKIKDFLGNLVPRTES
41. (13 . 24)     MGTMKTQRDGPSLGRWSLVLLLLGLVMPLAIVAQVLSYQEAVLRAIEGINQRSSDANLYRLLDLDPRPTMDGDPDTPKPVSFTVKETVCPRTTQKSPEDCDFKEDGLVKRCVGTVTLNQARDSFDISCDKDNRRFARLGKFFRKVKKKIGGGLKKIGQKIKDFLGNLVPRTAS
42. (7 . 25)      MGTMKTQRDSPSLGRWSLVLLLLGLVMPLAIVAQVLSYQEAVLRAIDGINQRSSDANLYRLLDLDPRPTMDGDPDTPKPVSFTVKETVCPRTTQKSPEDCDFKEDGLVKRCVGTVILNQARDSFDISCDKDNRRSARLGNFFRKVKEKIGGGLKKVGQKIKDFLGNLVPRTAS
43. (16 . 42)     MGTMKTQRDSPSLGRWSLVLLLLGLVMPLAIVAQVLSYQEAVLRAIDGINQRSSDANLYRLLDLDPRPTMDGDPDTPKPVSFTVKETVCPRTTQKSPEDCDFKEDGLVKRCVGTVILNQARDSFDISCDKDNRRSARLGNFFRKVKEKIGGGLKKVGQKIKDFLGNLVPRTAS
44. (4 . 43)      MGTMKTQRDSPSLGRWSLVLLLLGLVMPLAIVAQVLSYQEAVLRAIDGINQRSSDANLYRLLDLDPRPTMDGDPDTPKPVSFTVKETVCPRTTQKSPEDCDFKEDGLVKRCVGTVILNQARDSFDISCDKDNRRFARLGNFFRKVKEKIGGGLKKVGQKIKDFLGNLVPRTAS
45. (20 . 44)     MGTMKTQRDGPSLGRWSLVLLLLGLVMPLAIVAQVLSYQEAVLRAIDGINQRSSDANLYRLLDLDPRPTMDGDPDTPKPVSFTVKETVCPRTTQKSPEDCDFKEDGLVKRCVGTVILNQARDSFDISCDKDNRRFARLGNFFRKVKKKIGGGLKKVGQKIKDFLGNLVPRTAS
46. (41 . 45)     MGTMKTQRDGPSLGRWSLVLLLLGLVMPLAIVAQVLSYQEAVLRAIDGINQRSSDANLYRLLDLDPRPTMDGDPDTPKPVSFTVKETVCPRTTQKSPEDCDFKEDGLVKRCVGTVTLNQARDSFDISCDKDNRRFARLGNFFRKVKKKIGGGLKKIGQKIKDFLGNLVPRTAS
47. (40 . 46)     MGTMKTQRDGPSLGRWSLVLLLLGLVMPLAIVAQVLSYQEAVLRAIDGINQRSSDANLYRLLDLDPRPTMDGDPDTPKPVSFTVKETVCPRTTQKSPEDCDFKEDGLVKRCVGTVTLNQARDSFDISCDKDNRRFARLGNFFRKAKEKIGRGLKKIGQKIKDFLGNLVPRTES
48. (38 . 47)     MGTMKTQRDGPSLGRWSLVLLLLGLVMPLAIVAQVLSYQEAVLRAIDGINQRSSDANLYRLLDLDPRPTMDGDPDTPKPVSFTVKETVCPRTTQKSPEDCDFKEDGLVKRCVGTVTLNQARDSFDISCDKDNRRFARLGNFFRKAKEKIGRGLKKIGQKIKDFLGNLVPRTES
49. (2 . 22)      MGTMKTQRDGPSLGRWSLLLLLLGLTMPLAVIGRVLSYQEAVLRAVDGLNQRSSDANLYRLLNLDPRPTMDGDPDTPKPVSFTVKETVCPRTIQRSPEECDFKEDGLVKWCVGTVTLNQAKDSFDISCDKDKRKVARLGDILQKAGEKIGGGLKKIGQKIKDFFGKLAPRTES
50. (17 . 49)     MGTMKTQRDGPSLGRWSLLLLLLGLTMPLAVIARVLSYQEAVLRAVDGLNQRSSDANLYRLLNLDPRPTMDGDPDTPKPVSFTVKETVCPRTIQRSPEECDFKEDGLVKWCVGTVTLNQAKDSFDISCDKDKRKVARLGDILQKAGEKIGGGLKKIGQKIKDFFGKLAPRTES
51. (14 . 50)     MGTMKTQRDGPSLGRWSLVLLLLGLTMPLAIVAQVLSYQEAVLRAVDGLNQRSSDANLYRLLDLDPRPTMDGDPDTPKPVSFTVKETVCPRTIQRSPEECDFKEDGLVKWCVGTVTLNQAKDSFDISCDKDKRKVARLGDILQKAGEKIGRGLKKIGQKIKDFFGKLAPRTES
52. (12 . 51)     MGTMKTQRDGPSLGRWSLVLLLLGLTMPLAIVAQVLSYQEAVLRAVDGLNQRSSDANLYRLLDLDPRPTMDGDPDTPKPVSFTVKETVCPRTIQRSPEECDFKEDGLVKRCVGTVTLNQARDSFDISCDKDKRKVARLGDFLQKAREKIGRGLKKIGQKIKDFLGKLAPRTES
53. (48 . 52)     MGTMKTQRDGPSLGRWSLVLLLLGLVMPLAIVAQVLSYQEAVLRAIDGINQRSSDANLYRLLDLDPRPTMDGDPDTPKPVSFTVKETVCPRTTQQSPEECDFKEDGLVKRCVGTVTLNQARDSFDISCDKDNRRFARLGNFFRKAREKIGRGLKKIGQKIKDFLGNLVPRTES
54. (23 . 28)     ---METQRDGSFLGRWSLVLLLLGLVMPLATVAQALSYQEAVLRAVDGFNQRSSEANLYRLLDLDQQPTMDDDPDTPKPVSFTVKETVCPRTTQQPPEQCDFKEDGLVKRCVGTVTLDQARGSFDISCDENTRRAARLGGFFRKAGEKIGRKLKNIGRKIKDFLQNLVPRTES
```

Charge changes between nodes

Aves			
From node	To node	Propiece	Mature peptide
25	7	-1.00	-1.00
25	9	+0.91	+0.00
26	25	-0.91	-0.00
26	10	+1.00	-4.91
27	26	-1.91	+1.00
27	12	-1.91	+1.13
28	27	-0.09	0
28	3	+0.91	-2.00
29	28	+1.00	0
29	6	+0.00	-2.00
30	8	+3.09	-1.00
30	11	+3.00	+1.00
31	29	+1.00	-0.00
31	30	-0.10	-1.00
32	31	0	+1.00
32	5	+1.09	-1.00
33	32	0	0
33	4	-0.19	-3.00
34	1	-1.91	-0.91
34	2	-0.00	0
35	33	+0.00	+1.00
35	34	+1.82	+0.04
36	13	0	+1.00
36	14	0	+0.00
37	36	-1.91	0
37	16	+1.00	0
38	37	-1.00	0
38	15	-1.00	-1.00
39	17	0	+1.00
39	22	+1.00	0
40	18	-0.18	+0.09
40	23	-0.18	-0.00
41	20	+1.09	-1.91
41	21	+0.18	-2.82
42	19	-1.00	0
42	41	-0.09	+1.00
43	40	+0.18	+0.00
43	42	+0.09	+0.00
44	24	+2.00	-1.91
44	43	+1.00	-1.00
45	39	+2.00	-1.00
45	44	0	+1.00
46	38	+6.99	-3.00
46	45	0	0

Primates			
From node	To node	Propiece	Mature peptide
29	15	0	0
29	21	0	0
30	29	0	0
30	1	0	0
31	30	0	0
31	27	0	0
32	3	0	0
32	19	0	-2.00
33	31	-0.00	-0.00
33	32	-0.91	-2.00
34	8	0	0
34	26	0	0
35	33	+1.00	-0.09
35	34	0	0
36	10	0	0
36	11	0	0
37	9	0	0
37	36	0	0
38	35	+1.00	0
38	37	0	0
39	5	0	+2.00
39	6	0	0
40	39	0	0
40	18	0	-1.00
41	13	0	0
41	24	0	0
42	7	0	0
42	25	0	0
43	16	0	0
43	42	0	0
44	4	0	0
44	43	0	0
45	20	-1.00	0
45	44	0	-2.00
46	41	+0.00	+1.00
46	45	0	0
47	40	+1.00	0
47	46	0	+2.00
48	38	+1.00	-1.91
48	47	0	0
49	2	0	+2.00
49	22	-1.91	+0.00
50	17	-2.00	+1.00
50	49	0	0
51	14	+0.00	-2.00
51	50	+2.00	-1.00
52	12	+1.00	-0.00
52	51	-1.00	-1.00
53	48	+1.00	-0.00
53	52	+2.00	-1.00
54	23	-2.91	+1.00
54	28	+2.00	-3.99
