# Supplementary figures and images for: Evolution of the avian β-defensin and cathelicidin genes
Source: BMC Evol Biol. 2015 Sep 15;15:188. doi: 10.1186/s12862-015-0465-3 (PMC4571063; doi:10.1186/s12862-015-0465-3)

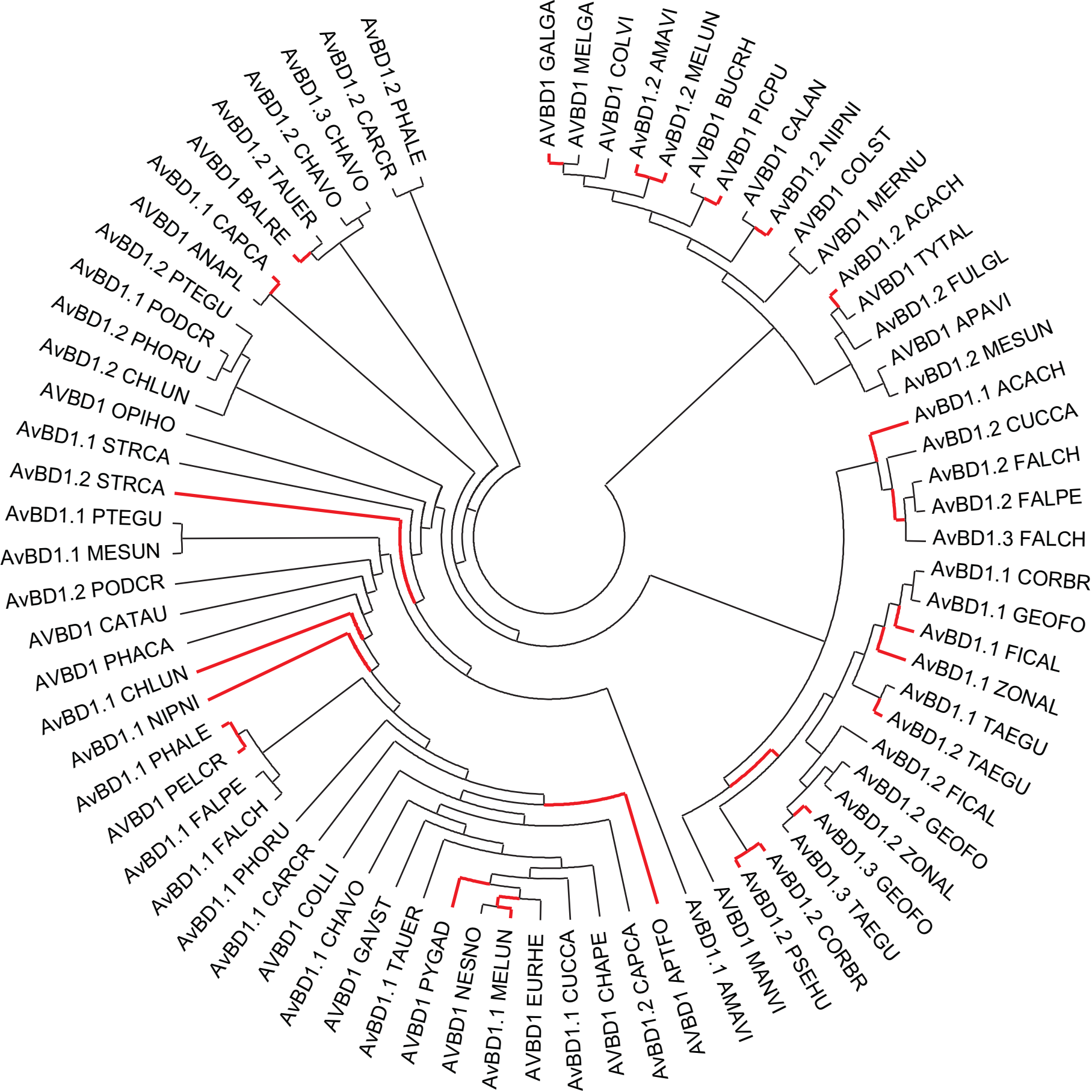

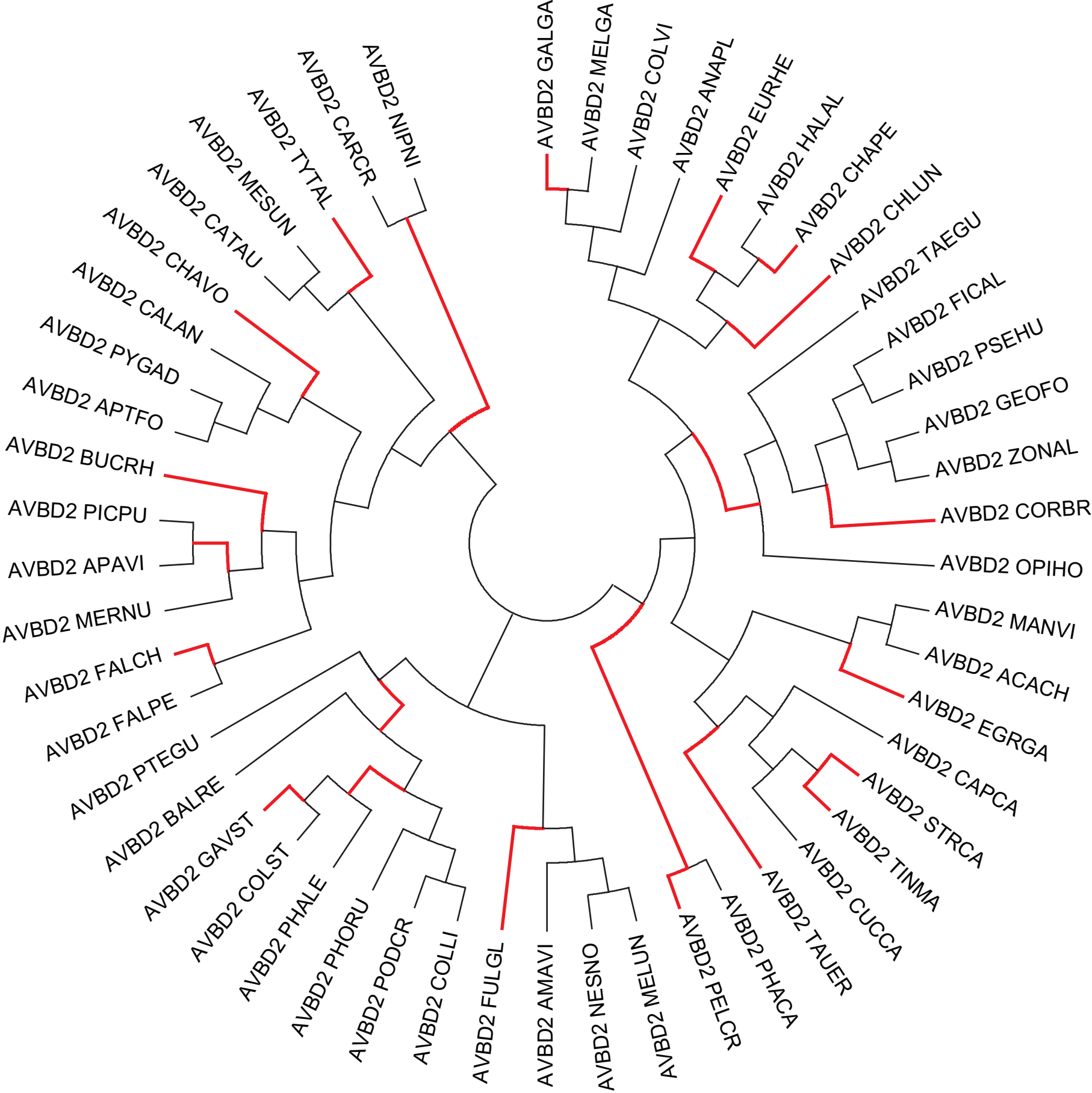

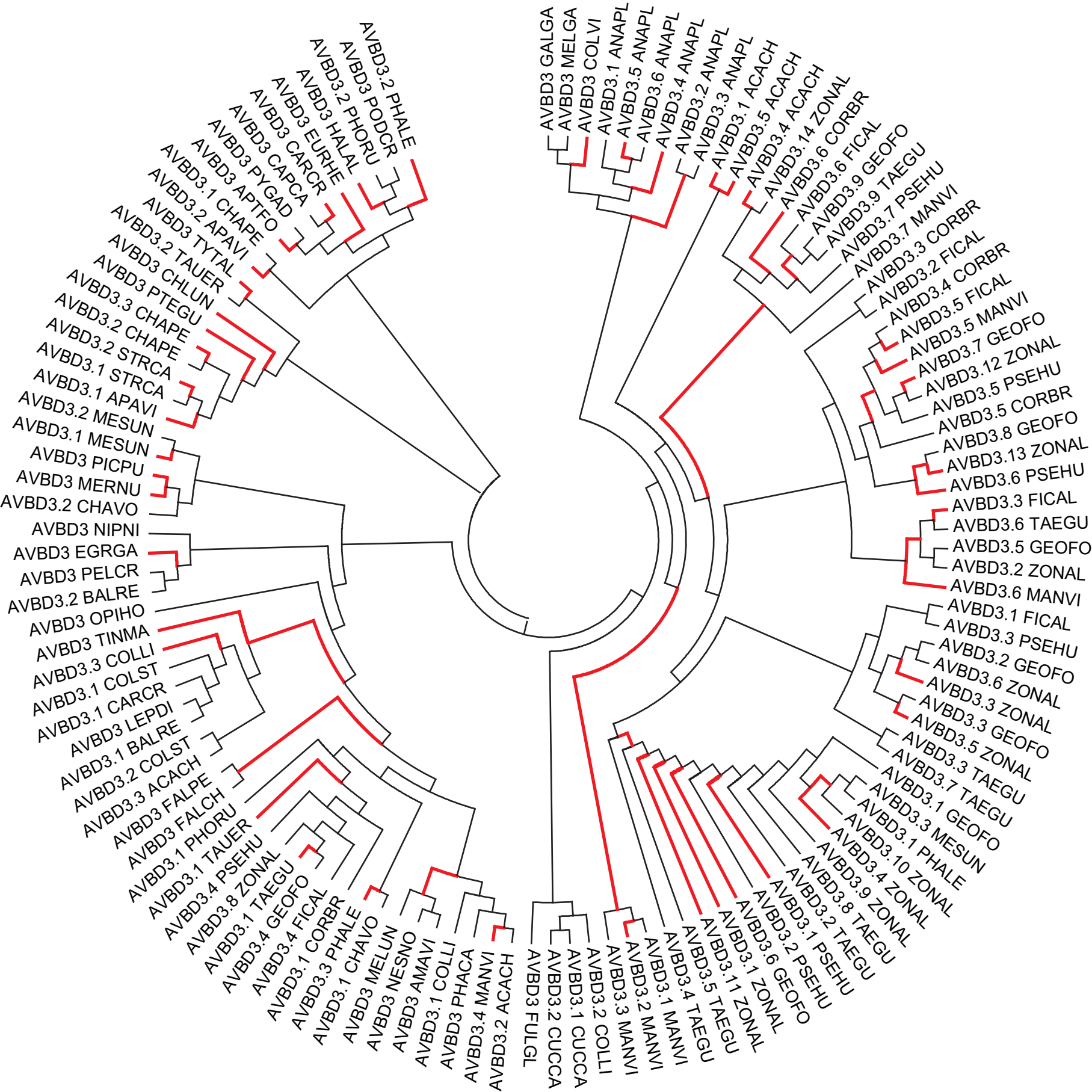

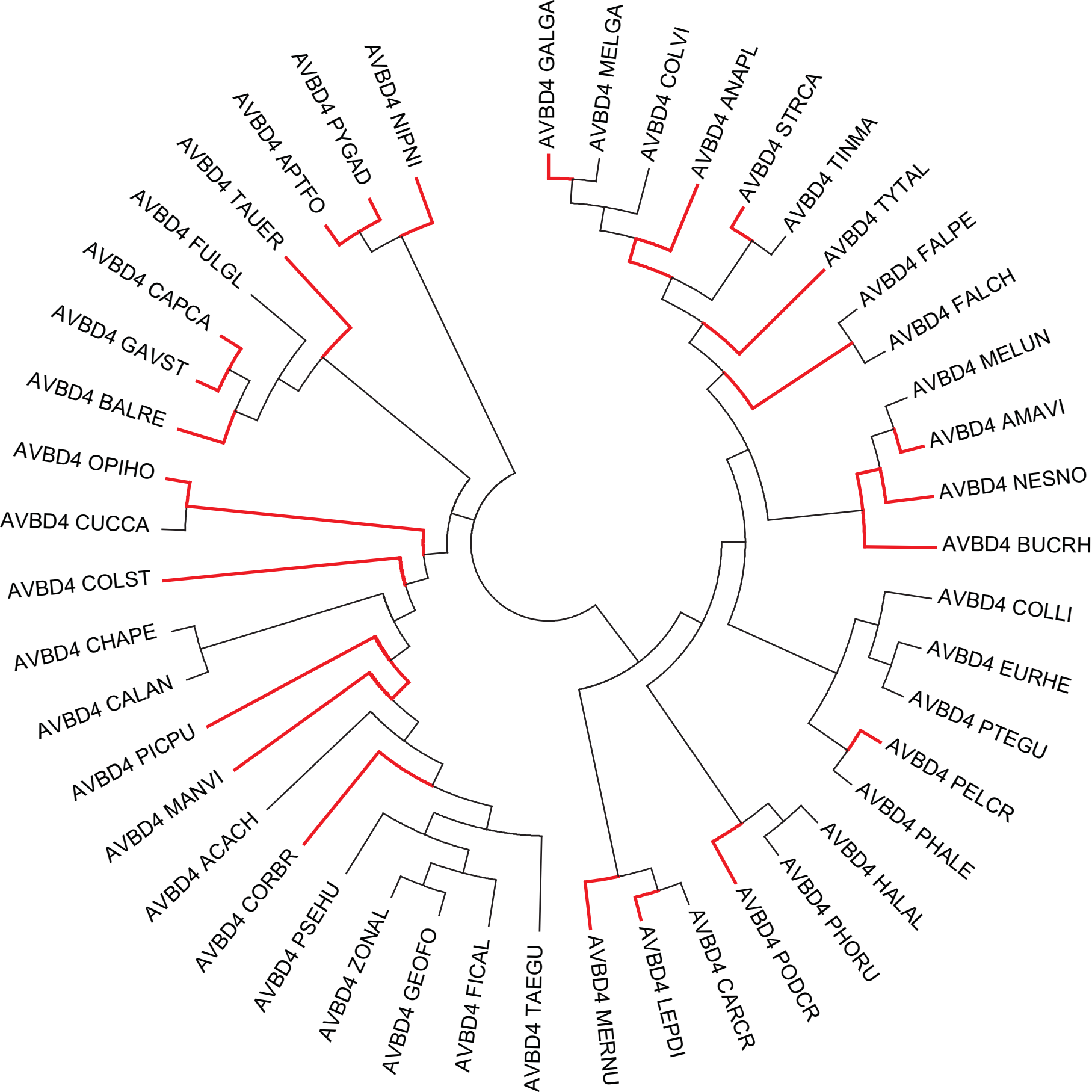

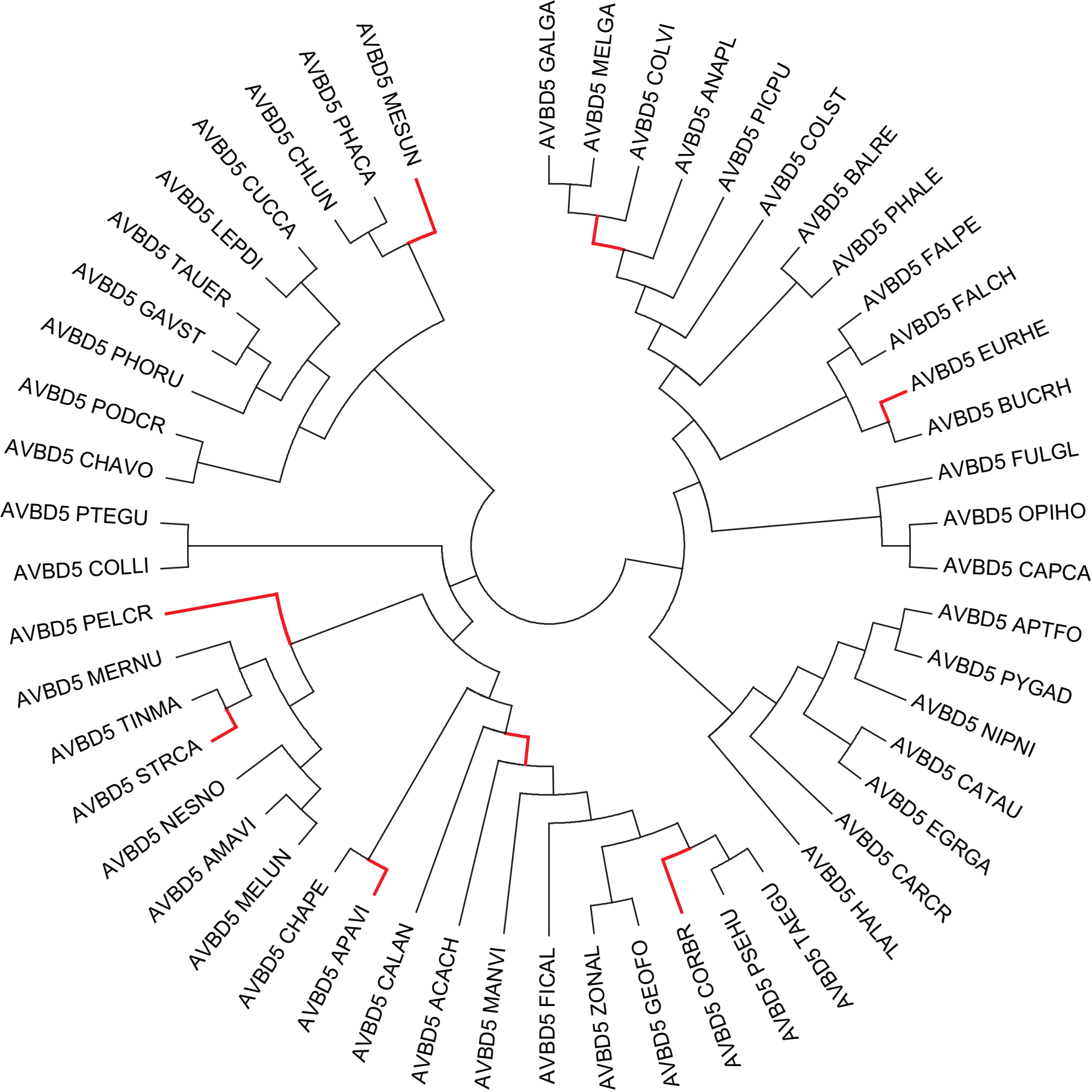

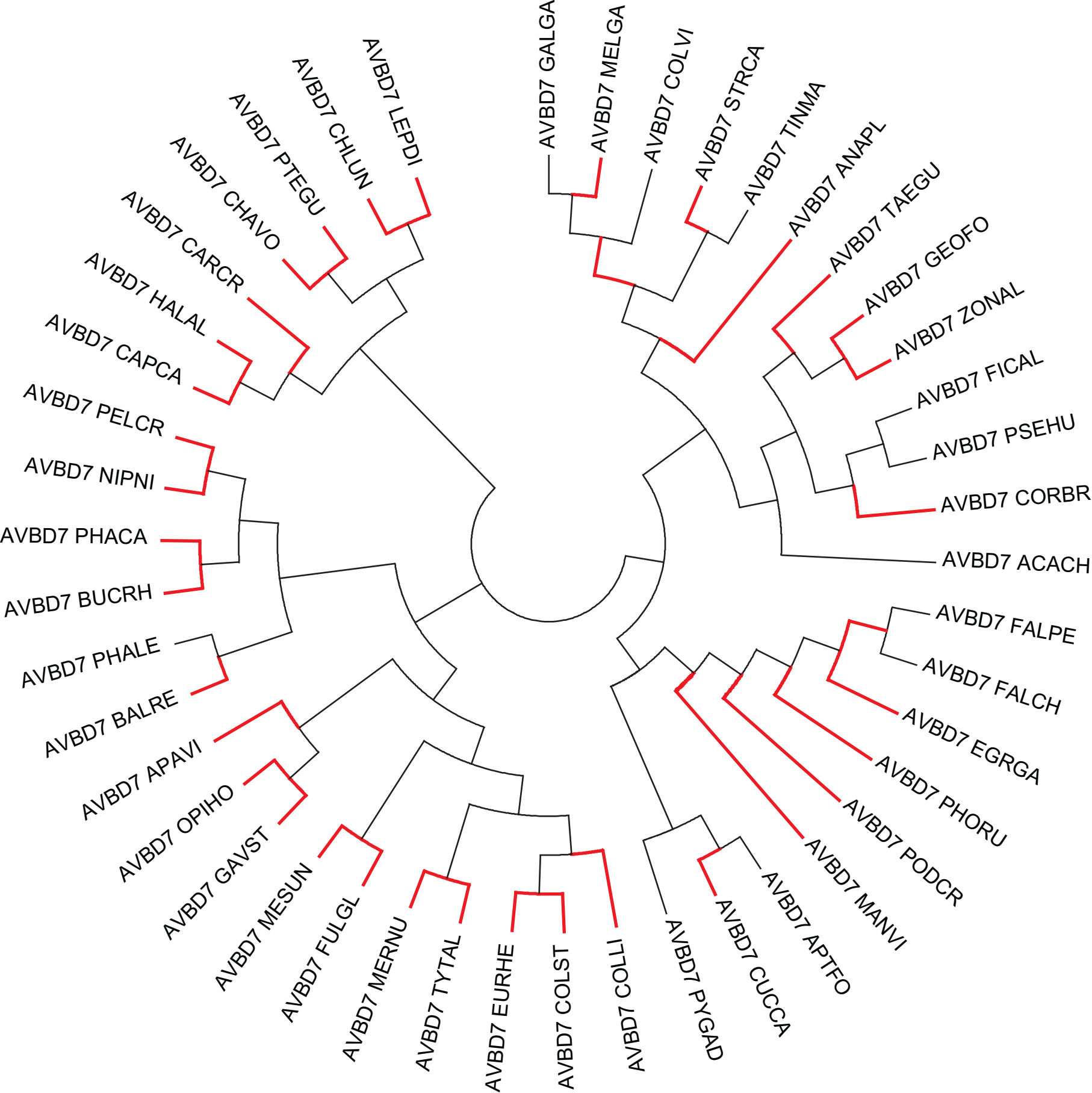

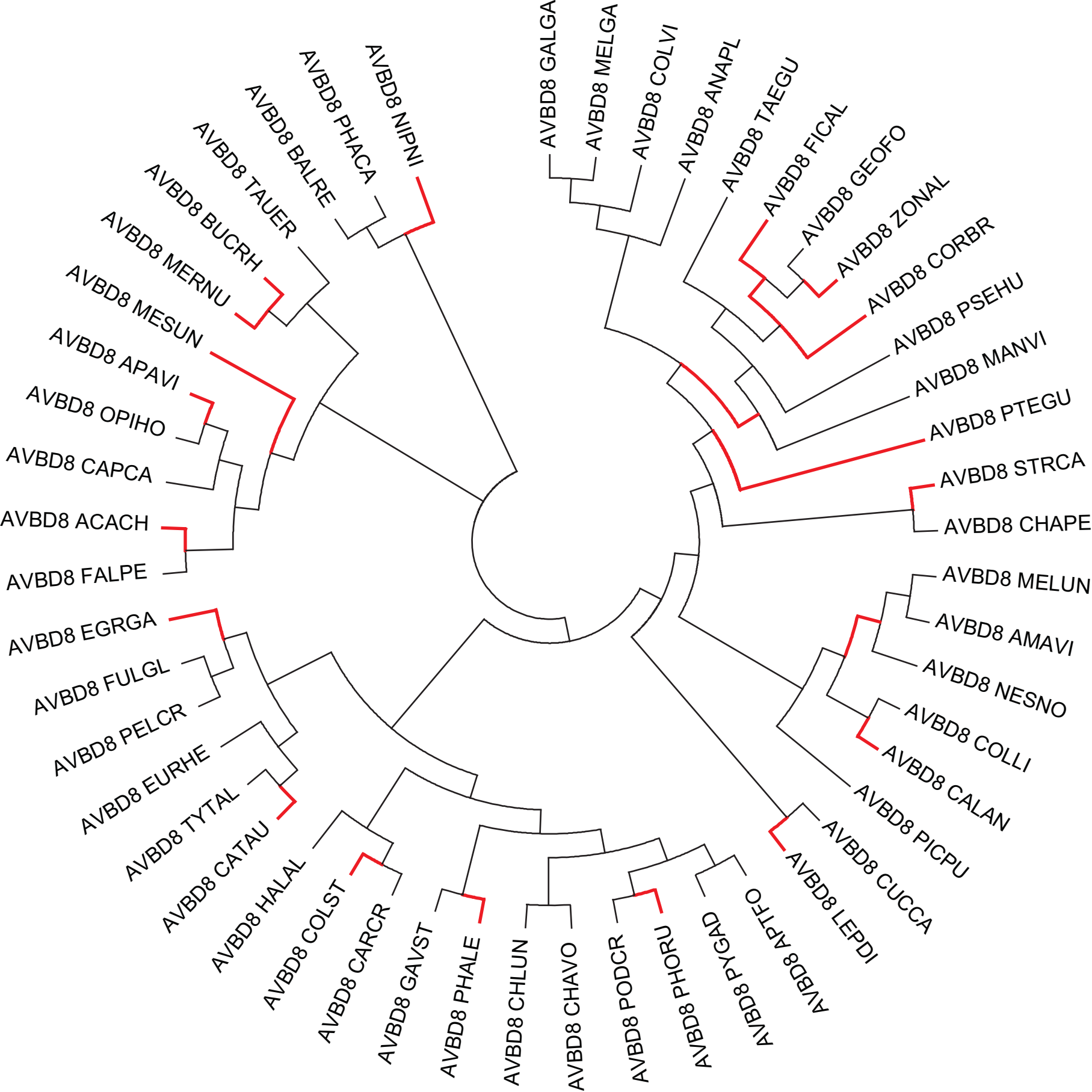

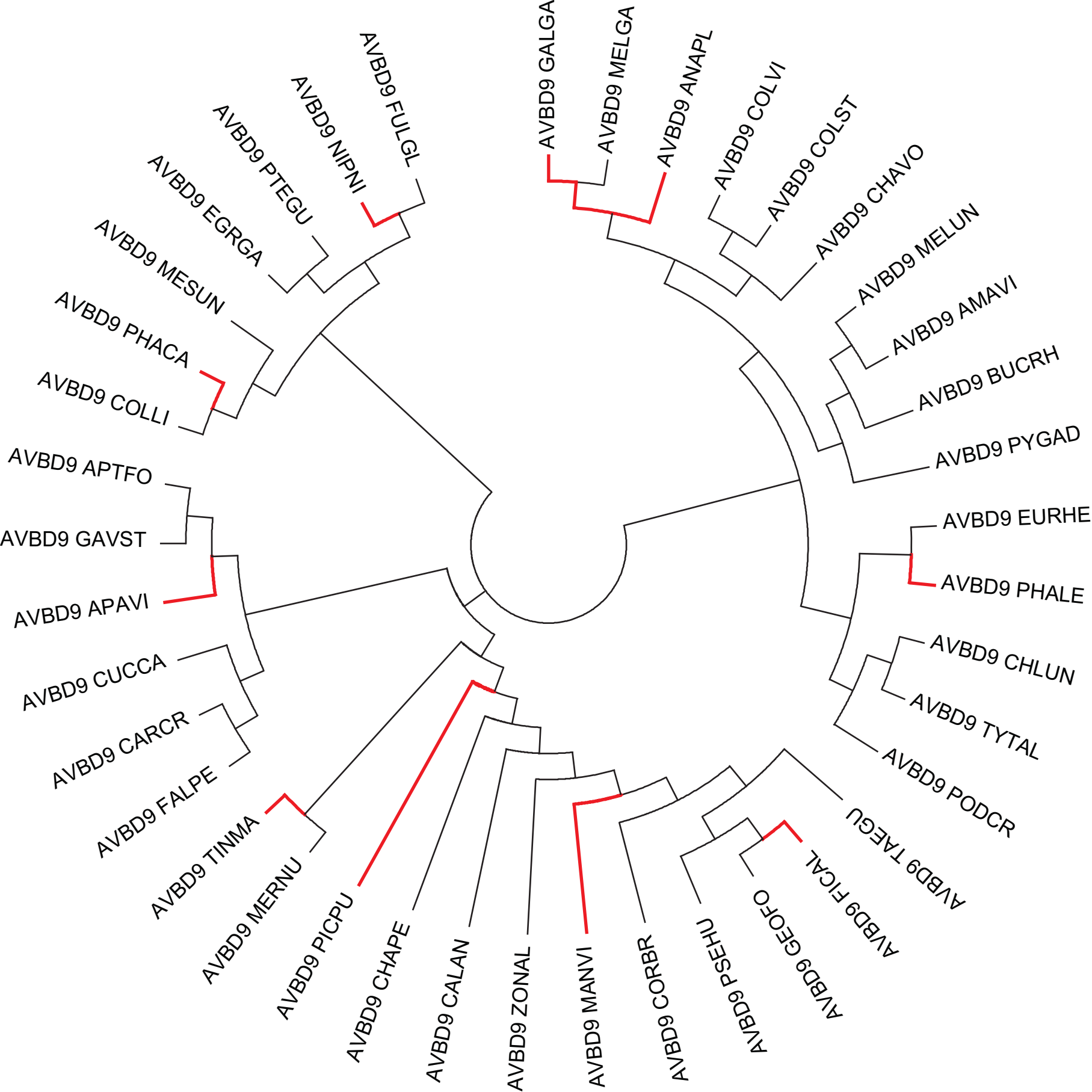

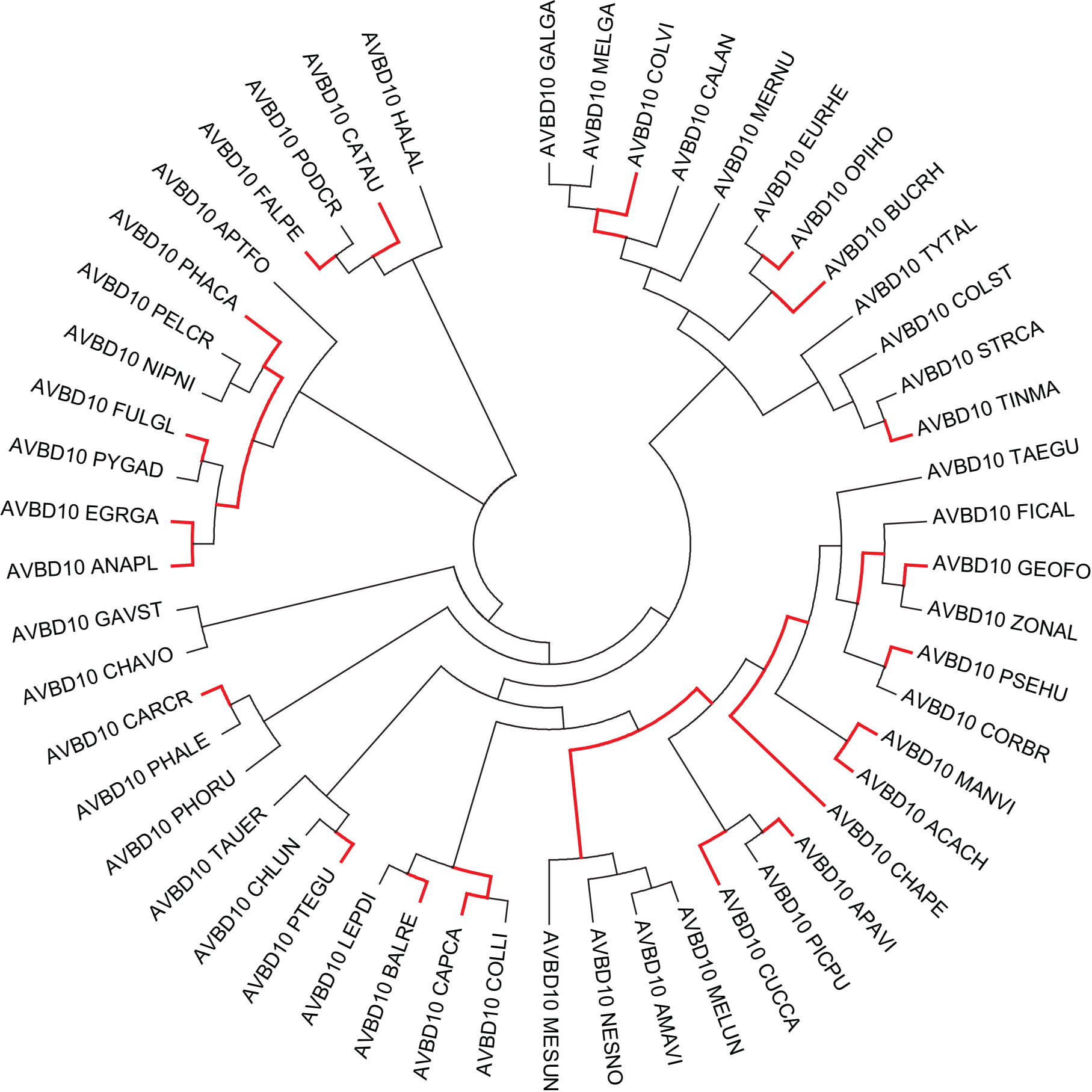

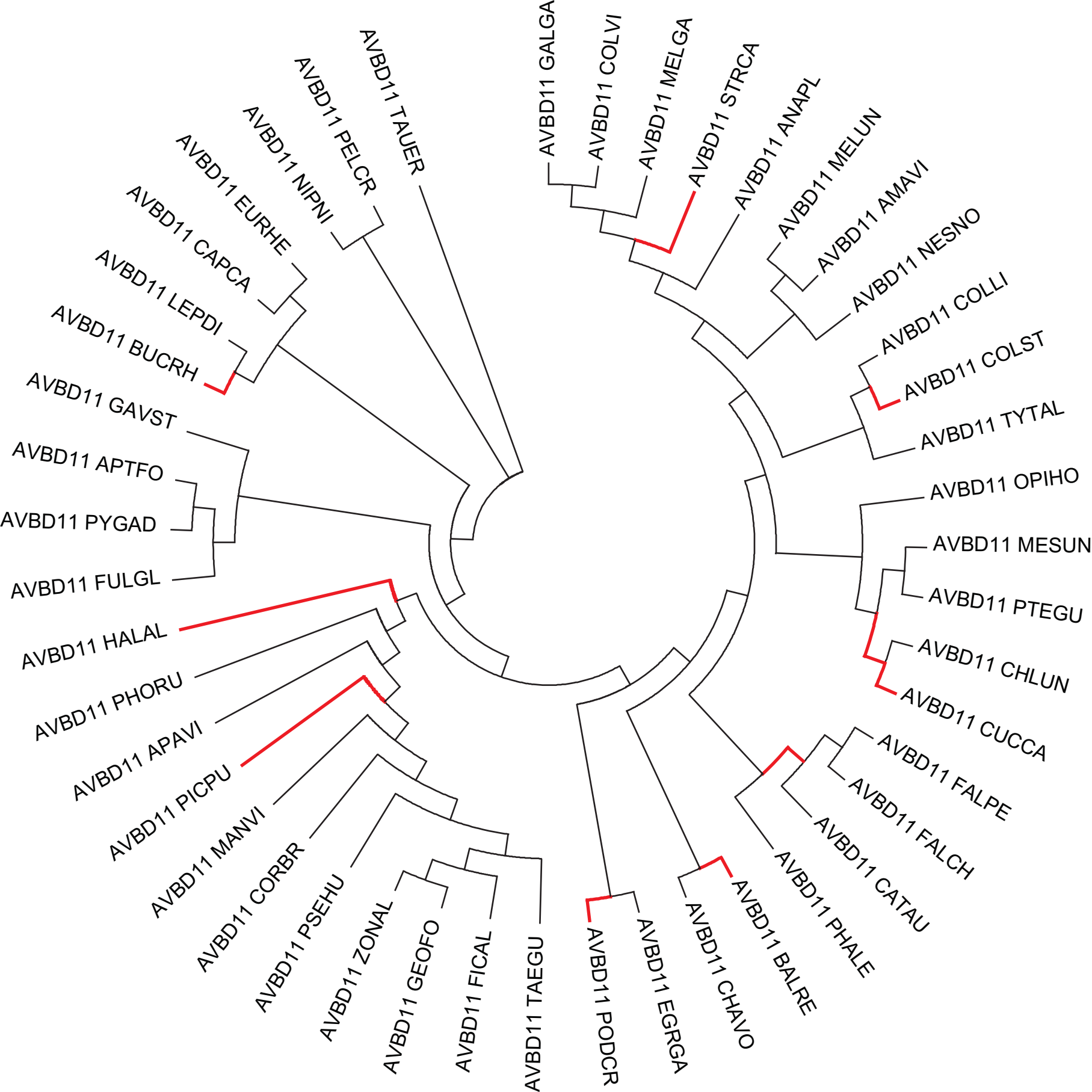

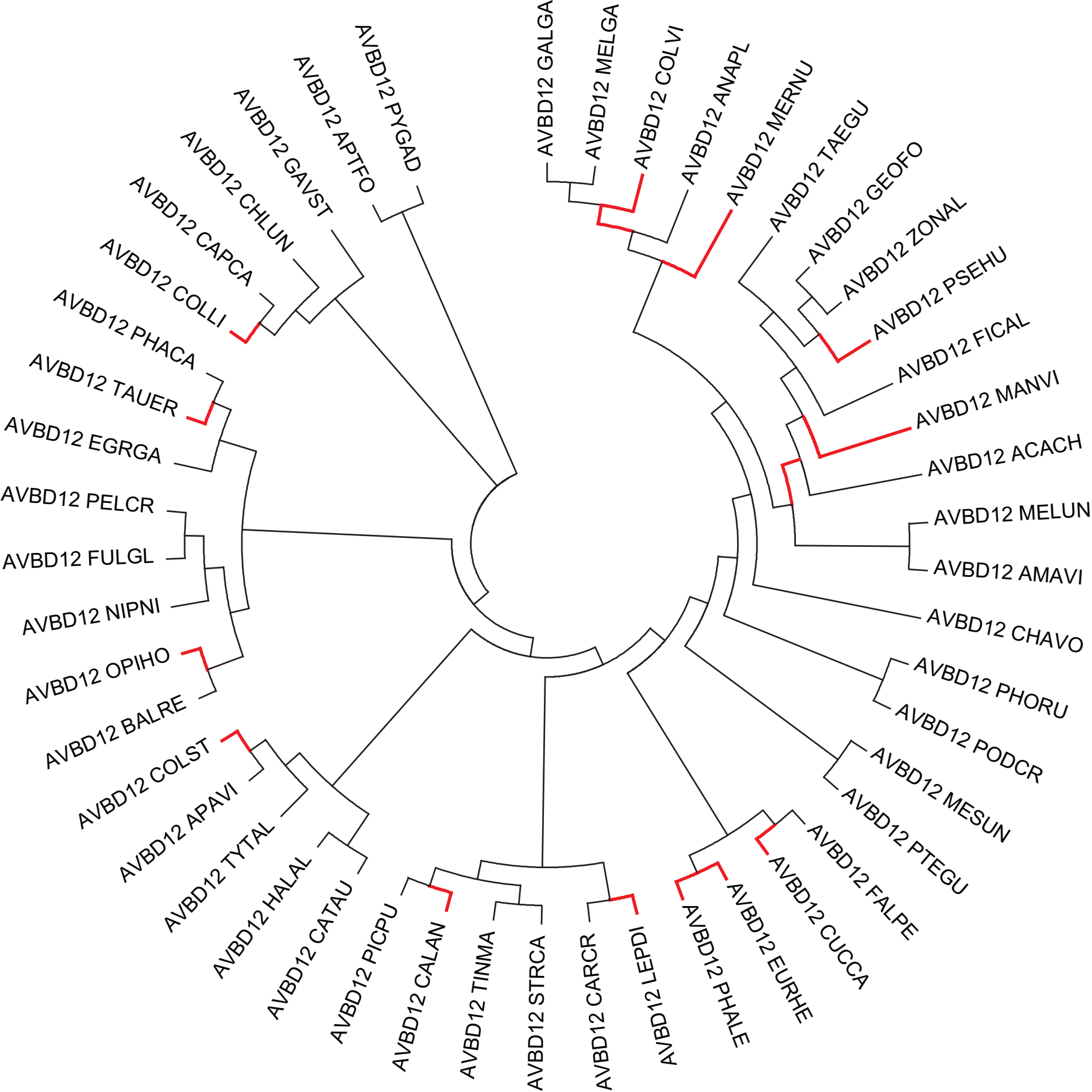

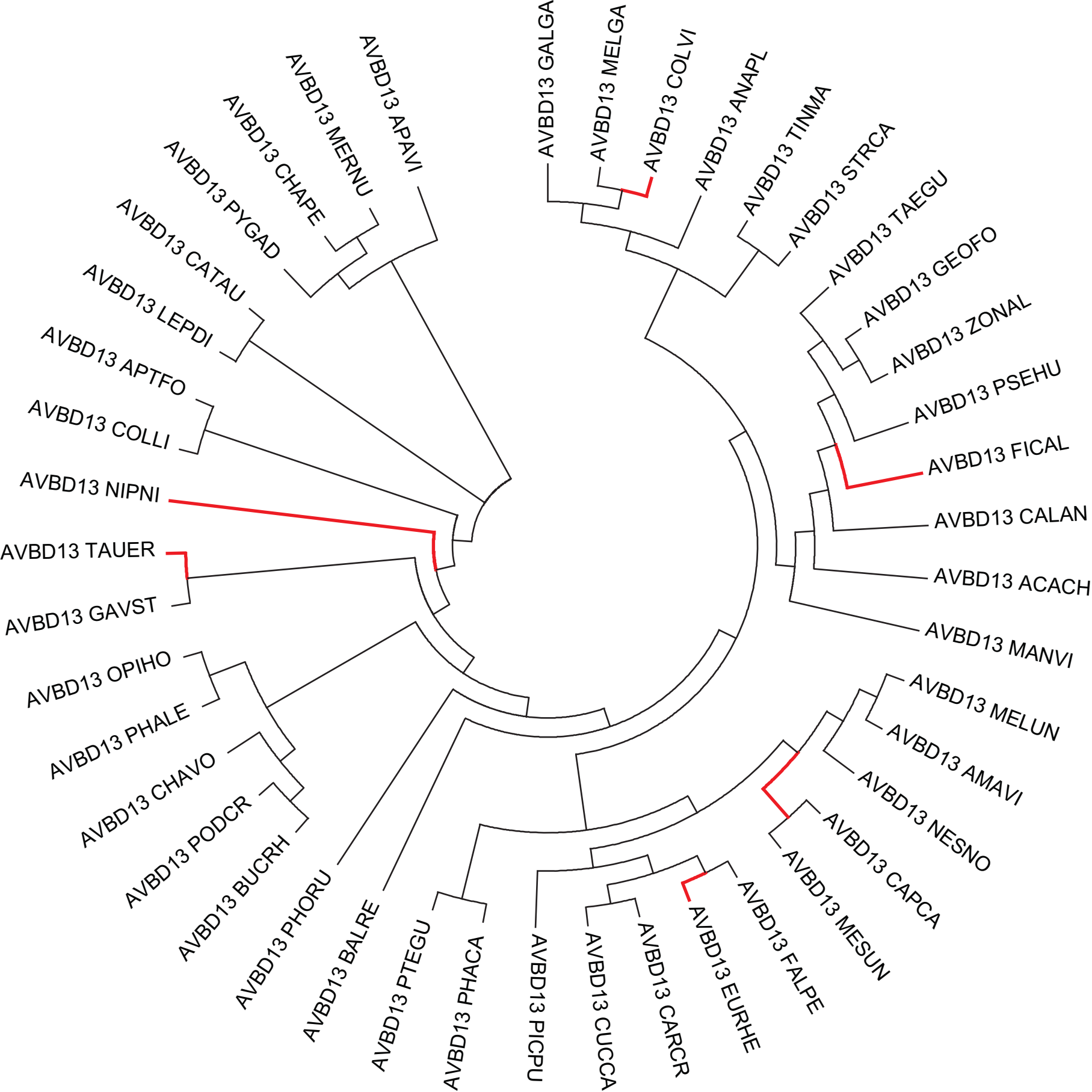

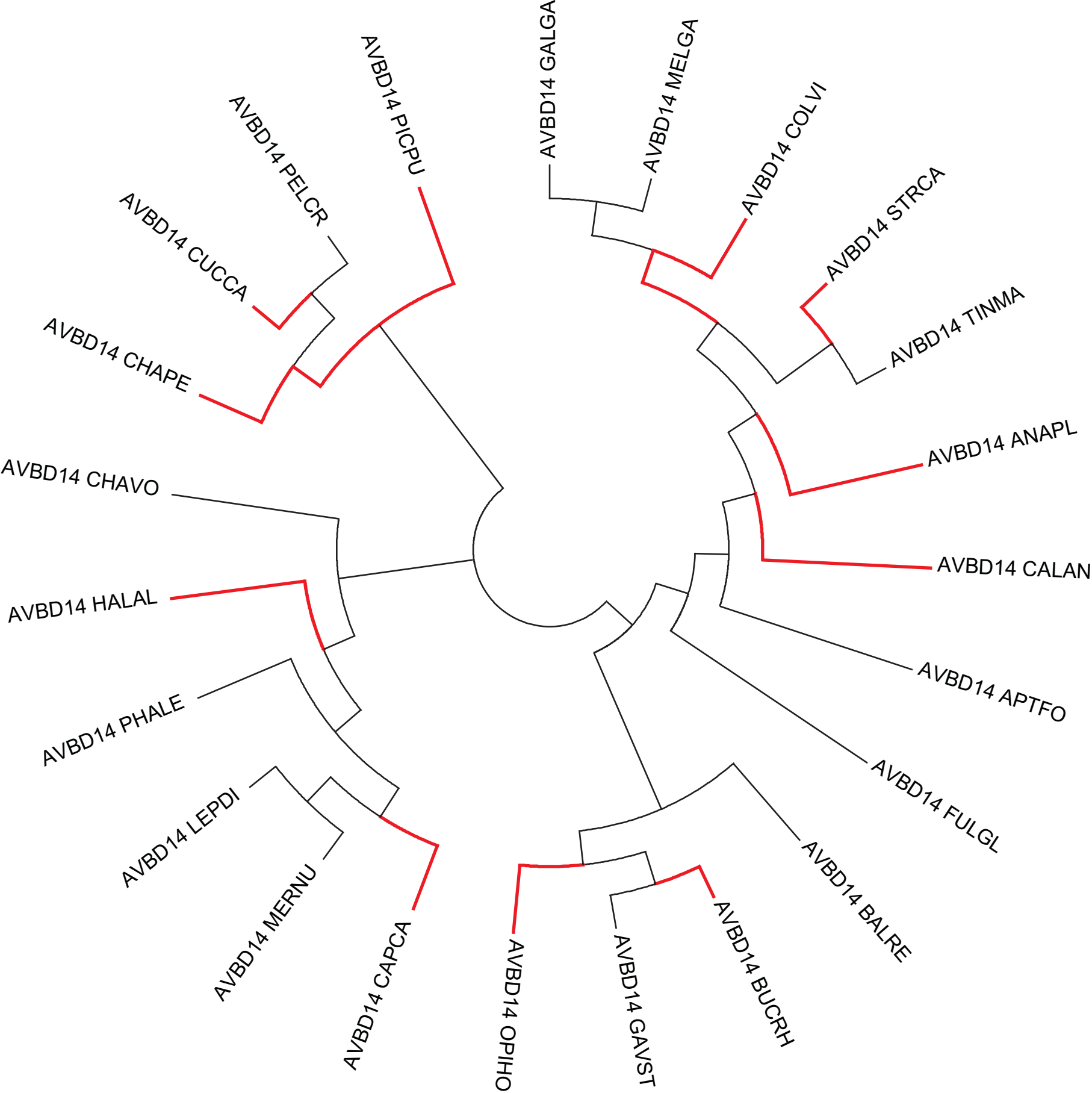

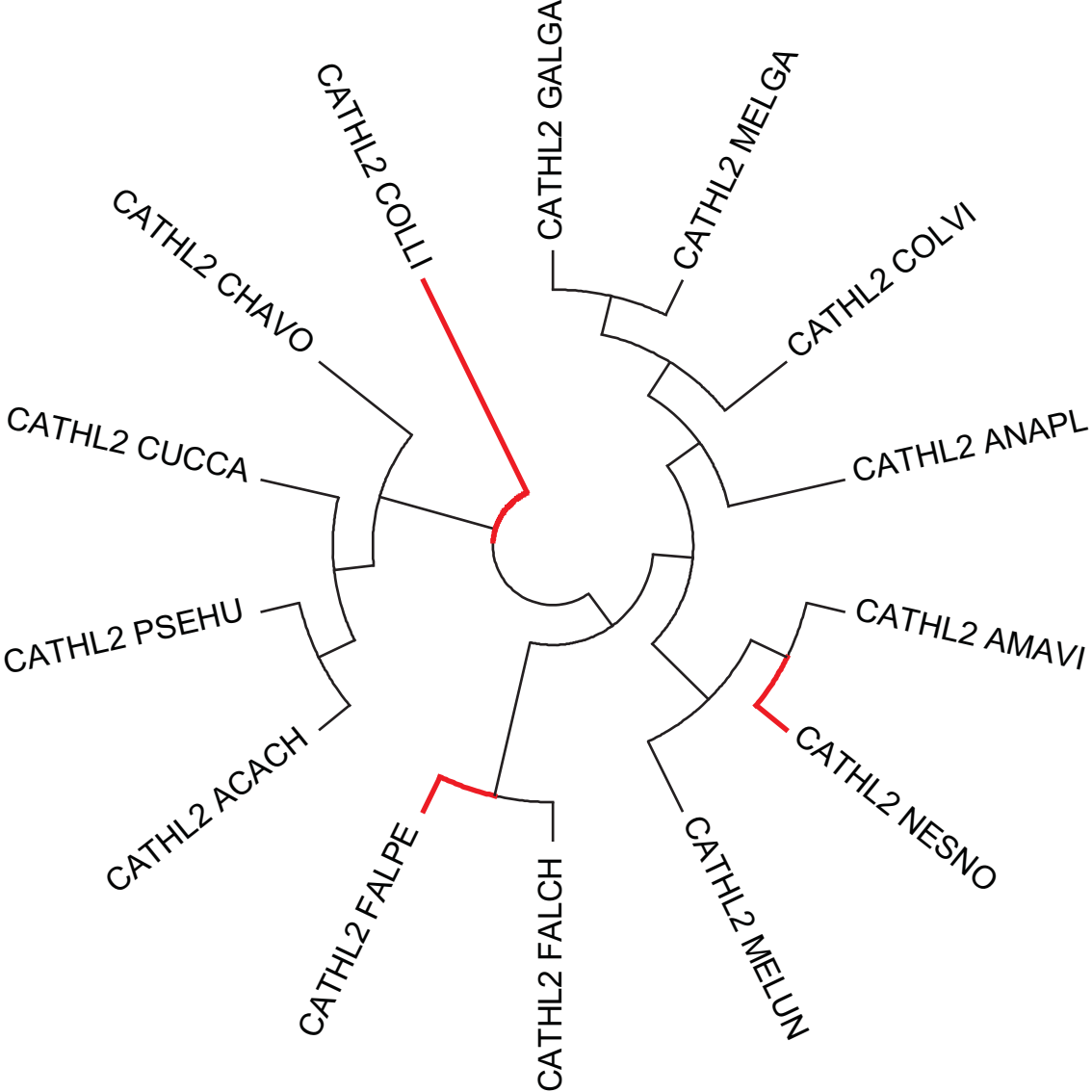

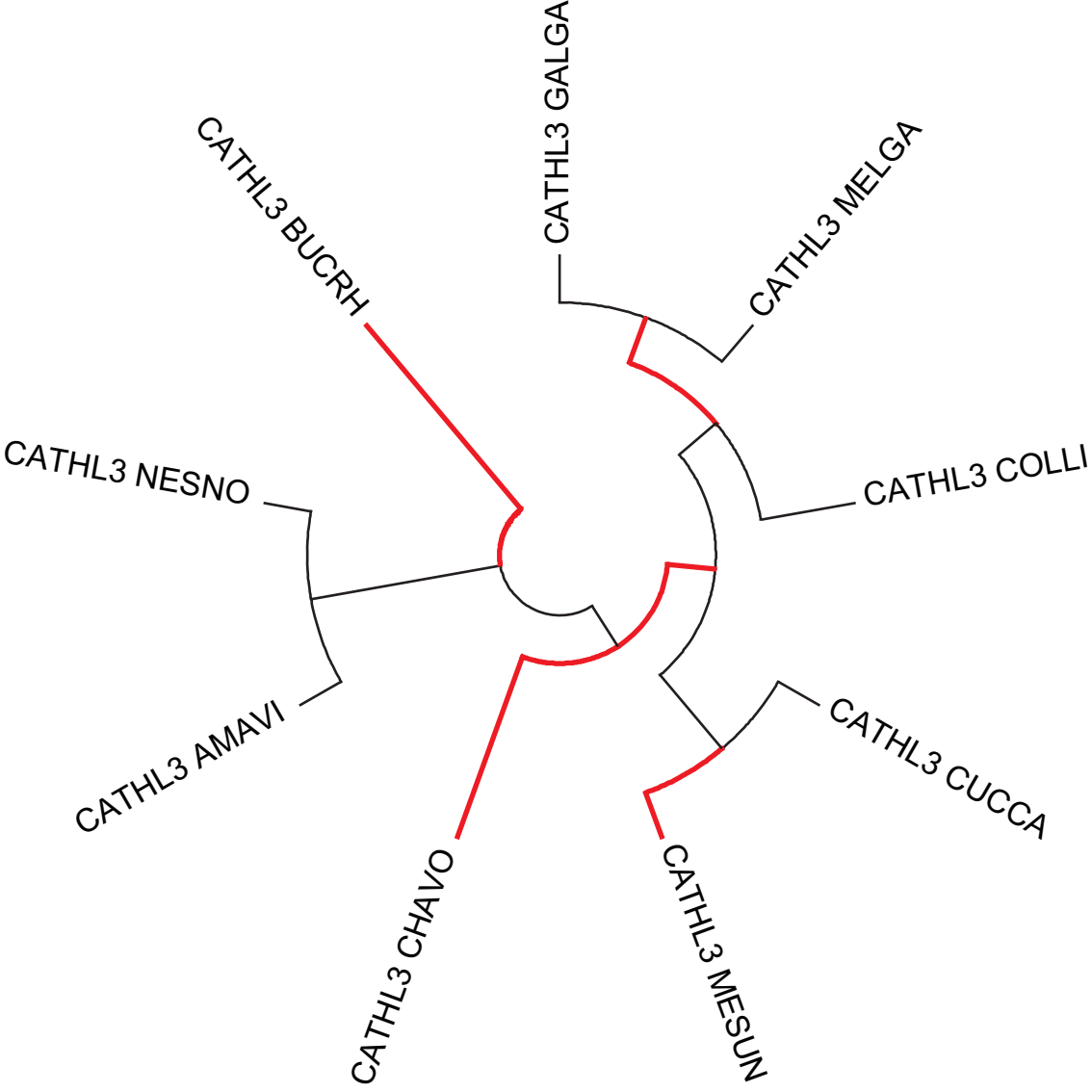

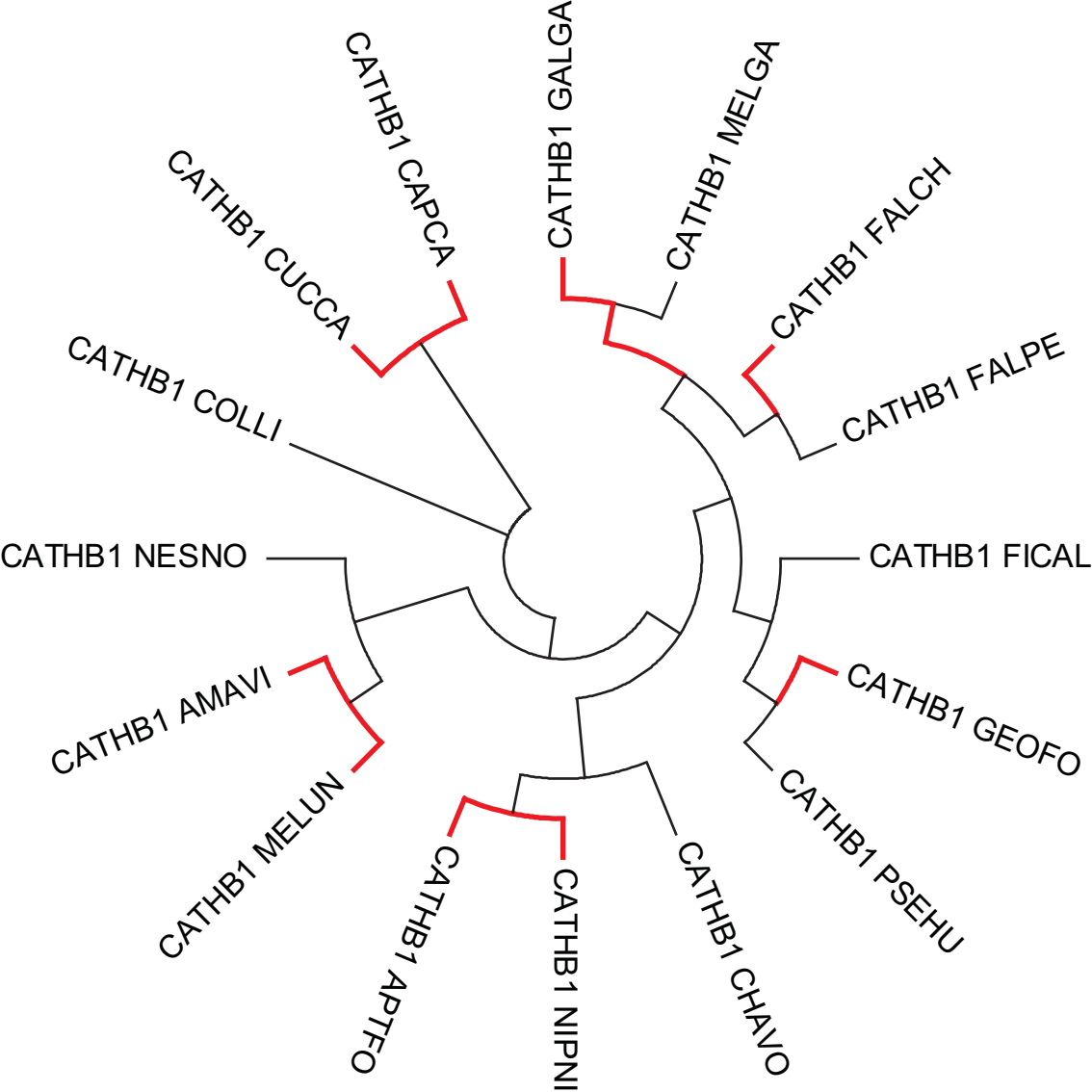

Supplement: Additional file 8: — Phylogenetic trees of avian β-defensin and cathelicidin genes. Branches with evidence suggesting episodic diversifying selection are highlighted in red. (PDF 1444 kb) [file 12862_2015_465_MOESM8_ESM.pdf]
